# Multiomics analysis identifies oxidative phosphorylation as a cancer vulnerability arising from myristoylation inhibition

**DOI:** 10.1186/s12967-024-05150-6

**Published:** 2024-05-07

**Authors:** Erwan Beauchamp, Jay M. Gamma, Christopher R. Cromwell, Eman W. Moussa, Rony Pain, Morris A. Kostiuk, Claudia Acevedo-Morantes, Aishwarya Iyer, Megan Yap, Krista M. Vincent, Lynne M. Postovit, Olivier Julien, Basil P. Hubbard, John R. Mackey, Luc G. Berthiaume

**Affiliations:** 1 Pacylex Pharmaceuticals Inc., Edmonton, AB Canada; 2 Department of Medicine and Pathology, Faculty of Medicine and Dentistry, University of Alberta, Edmonton, AB Canada; 3 Department of Pharmacology, Faculty of Medicine and Dentistry, University of Alberta, Edmonton, AB Canada; 4 Department of Biochemistry, Faculty of Medicine and Dentistry, University of Alberta, Edmonton, AB Canada; 5 Department of Cell Biology, Faculty of Medicine and Dentistry, University of Alberta, Edmonton, AB Canada; 6 Department of Oncology, Faculty of Medicine and Dentistry, University of Alberta, Edmonton, AB Canada

**Keywords:** N-myristoylation, N-myristoyltransferase, NMT inhibitor (NMTI), PCLX-001 (zelenirstat), Cancer, Complex I, NDUFAF4, Oxidative phosphorylation, Respiration

## Abstract

**Background:**

In humans, two ubiquitously expressed N-myristoyltransferases, NMT1 and NMT2, catalyze myristate transfer to proteins to facilitate membrane targeting and signaling. We investigated the expression of *NMT*s in numerous cancers and found that *NMT2* levels are dysregulated by epigenetic suppression, particularly so in hematologic malignancies. This suggests that pharmacological inhibition of the remaining NMT1 could allow for the selective killing of these cells, sparing normal cells with both NMTs.

**Methods and results:**

Transcriptomic analysis of 1200 NMT inhibitor (NMTI)-treated cancer cell lines revealed that NMTI sensitivity relates not only to *NMT2* loss or *NMT1* dependency, but also correlates with a myristoylation inhibition sensitivity signature comprising 54 genes (MISS-54) enriched in hematologic cancers as well as testis, brain, lung, ovary, and colon cancers. Because non-myristoylated proteins are degraded by a glycine-specific N-degron, differential proteomics revealed the major impact of abrogating *NMT1* genetically using CRISPR/Cas9 in cancer cells was surprisingly to reduce mitochondrial respiratory complex I proteins rather than cell signaling proteins, some of which were also reduced, albeit to a lesser extent. Cancer cell treatments with the first-in-class NMTI PCLX-001 (zelenirstat), which is undergoing human phase 1/2a trials in advanced lymphoma and solid tumors, recapitulated these effects. The most downregulated myristoylated mitochondrial protein was NDUFAF4, a complex I assembly factor. Knockout of *NDUFAF4* or in vitro cell treatment with zelenirstat resulted in loss of complex I, oxidative phosphorylation and respiration, which impacted metabolomes.

**Conclusions:**

Targeting of both, oxidative phosphorylation and cell signaling partly explains the lethal effects of zelenirstat in select cancer types. While the prognostic value of the sensitivity score MISS-54 remains to be validated in patients, our findings continue to warrant the clinical development of zelenirstat as cancer treatment.

**Supplementary Information:**

The online version contains supplementary material available at 10.1186/s12967-024-05150-6.

## Introduction

### N-myristoyltransferase inhibitors (NMTIs) in cancer

N-myristoylation is an essential [1] protein modification by the fourteen-carbon fatty acid myristate. N-myristoylation occurs on a newly exposed N-terminal glycine residue, either co-translationally [2] following the removal of the N-terminal initiator methionine residue or post-translationally during apoptosis after caspase cleavage [3]. By facilitating the movement of proteins from the cytosol to membranes, myristoylation plays key roles in cellular homeostasis, impacting not only protein-membrane interactions but also protein–protein interactions, lipid raft targeting, vesicular transport, signal transduction, and apoptosis regulation [4–8]. This process is catalyzed by two N-myristoyltransferases [9] (NMT1 and NMT2). In humans, over 200 proteins [10] are myristoylated at the N-terminal glycine residues. The fact that many proto-oncogenic proteins [e.g., such as Src-family kinases (SFKs) [11] and c-Abl [12]] depend on their myristoylation for full functionality and that non-myristoylated v-Src failed to transform cells [13] originally suggested that NMTs could be potential cancer targets [11, 14].

Until recently, hydroxy-myristate, Tris-DBA [15], B13 [16], and desloratidine [17] were the only chemical tools available to study myristoylation, but all of these compounds have been shown to be either weak or non-specific NMT inhibitors (NMTIs) [18]. Recently, two drugs and their families of NMTIs have been formally validated as potent and specific inhibitors: IMP-1088 (an indazole methanamine) and DDD85646 (a pyrazole sulfonamide) [18]. Given that proteins with exposed N-terminal glycine residues are degraded by a glycine-specific N-degron [19], NMTIs could thus promote the proteolytic removal of proto-oncogenic proteins and other growth-promoting proteins, and support their potential as cancer therapeutic agents.

PCLX-001 (also known as zelenirstat) is an investigational first-in-class small-molecule pan-NMTI, originally known as DDD86481 [20], which was licensed from the University of Dundee by Pacylex Pharmaceuticals, Inc. It is an analog of the above validated NMTI DDD85646 (also known as PCLX-002 or ICL1100013). Zelenirstat is undergoing human clinical trial evaluation for the treatment of advanced lymphoma and solid malignancies [21] (https://clinicaltrials.gov/ct2/show/NCT04836195).

### Using NMTIs to treat cancer: from hematologic cancers to solid tumors

The potential of PCLX-001 against hematologic cancers was revealed by the results of three independent viability screens performed on 300 cancer cell lines of multiple origins [22]. While PCLX-001 is highly effective at killing cancer cell lines originating from solid tumors, it is particularly effective at killing hematologic cancer cell lines in vitro and in vivo [22, 23]. The selective killing of lymphoma cells, a type of hematologic cancer cells, was further validated in numerous cell lines (CDX) and patient-derived (PDX) xenografts [22]. Mechanistically, PCLX-001 promoted the degradation of multiple SFKs, leading to the loss of survival signals downstream of the B-cell receptor (BCR) and death in B-cell lymphoma cells; however, the large number of NMT substrates permitted other potential contributory mechanisms of action.

Databases analyses revealed that *NMT2* expression levels varied significantly more than those of *NMT1*, and that hematologic cancer cell lines comprised the vast majority of the lowest *NMT2* expressing cells [22]. Since most hematologic cancer cells are *NMT2*-deficient, we originally hypothesized that by targeting the remaining NMT1 in NMT2-deficient hematologic cells, PCLX-001 could selectively kill these cells in a manner reminiscent of synthetic lethality [24], thereby sparing normal human cells with two functional NMTs.

Herein, we sought to develop a better understanding of what makes cancer cells sensitive to the action of the first-in-class NMTI PCLX-001 to identify future indications most likely to respond therapeutically and to investigate the mechanism of action of this potential new cancer drug. First, we evaluated how the *NMT* expression landscape affects the response of common cancer cells to NMTIs. Because NMT2 loss, which we now demonstrate to occur via epigenetic suppression, could not explain by itself the sensitivity of all types of cancer cells to PCLX-001, we hypothesized that other genes were involved. We have thus developed a gene signature to predict future cancer indications most likely to respond therapeutically to this new class of cancer drug. MISS-54, a collection of 54 genes enriched in NMTI sensitive cells, predicts that several solid tumor, including testis, lung, brain, ovarian, and colon cancers, could potentially benefit from NMTI therapy in addition to hematologic cancers including acute myeloid leukemia, AML, and lymphomas. Mechanistically, we found that CRISPR/Cas9 *NMT* KOs and PCLX-001 treatment drastically affect cellular proteomes, metabolomes, and transcriptomes, with a surprisingly dominant impact on the mitochondrial proteome and metabolism. More precisely, we show that genetic or pharmacologic abrogation of myristoylation mainly reduced the levels of mitochondrial respiratory complex I proteins rather than just cell signaling proteins. Furthermore, the knockout of the most downregulated myristoylated mitochondrial protein NDUFAF4, a complex I assembly factor, or treatment with PCLX-001 in vitro, resulted in loss of complex I, oxidative phosphorylation and respiration. The dual action of PCLX-001 targeting oxidative phosphorylation and cell signaling thus explains in part its toxicity in select cancer cells. While the prognostic value of the sensitivity score MISS-54 remains to be validated in patients to delineate future indications for PCLX-001 and possibly identify patients who could benefit from NMTI treatment, our findings warrant the continued clinical development of PCLX-001/zelenirstat as a cancer treatment.

## Results

### *NMT2* expression levels vary greatly in cancer, especially in hematologic cancers, and correlate with *NMT1* dependency and poor prognosis in lymphoma patients

**Fig. 1:**
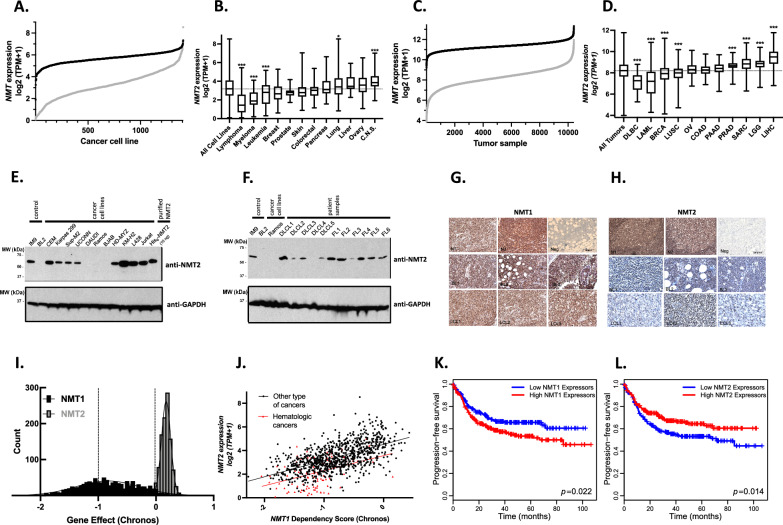
*NMT2* levels, but not *NMT1* levels, are decreased in numerous hematologic cancer cell lines and tumors. *NMT1 (*black) and *NMT2* (gray) transcript counts in cancer cell lines (**A**) (data extracted from Depmap 22Q4) and tumors (**C**) (data extracted from TCGA). *NMT2* mRNA expression sorted by cancer cell line (**B**) and tumor origin (**D**) (min-to-max box plot; one-way ANOVA comparison to all samples; ***p < 0.0001). NMT2 protein levels in a collection of local lymphocytic cell lines and solid lymphomas. NMT2 levels, assessed by western blot of 35 μg of cell lysate proteins in immortalized “normal” human B-cell line IM9, neoplastic B-cell lymphoma cell lines, leukemic T-cell lines (**E**), and lysates of various types of human solid lymphomas (**F**). Immunohistochemical staining for NMT1 (**G**) and NMT2 (**H**) in normal lymph nodes, Burkitt’s lymphoma (BL), and diffuse large B-cell lymphoma (DLBCL). Upper row: N1 and N2, normal lymph nodes; Neg, negative control in which primary antibody was omitted. Middle row: Three cases of BL (BL1–3). Lower row: Three cases of DLBCL (LCL1–3). Scale bars: 75 μ. Dependency scores (median non-essential KO effect is 0 and median essential KO effect is −1) obtained after *NMT* CRISPR knockout in 1078 cancer cell lines (**I**) *NMT1* dependency was significantly correlated with *NMT2* expression (*r* = 0.600; *p* < 0.0001) (***J***). Progression-free survival (PFS) Kaplan–Meier analysis of 470 DLBCL patients (GSE31312) with high (red) versus low (blue) *NMT1* (**K**) and *NMT2* (**L**) expression (high and low populations are calculated based on median expression). The *P* value was determined using the log-rank test

To explore the importance of N-myristoylation in cancer, we analyzed the expression of *NMTs* in CCLE and TCGA datasets (Fig. 1A, B). *NMT2* mRNA levels varied considerably (7 log2(TPM + 1)) in cell lines and tumors in comparison to *NMT1* mRNA levels (3 log2(TPM + 1)). *NMT2* expression was significantly lower in hematologic cancers (cell lines and tumors) in comparison to cancers of other origins (Fig. 1C, D, Additional file 1: Fig. S1). Because RNA expression does not always reflect protein levels [25], we investigated NMT protein levels in hematologic cancer cell lines and tumors using western blotting in a collection of randomly gathered hematologic cell lines, as well as in diffuse large B-cell lymphoma (DLBCL) and follicular lymphoma (FL) tumor lysates (Fig. 1E, F). Similarly, immunohistochemistry (IHC) was performed on randomly selected Burkitt’s lymphoma (BL) and DLBCL tumors (Fig. 1G, H). We found the loss of NMT2 protein was most evident in BL cell lines Daudi, Ramos and BJAB. In DLBCL and FL tumors, NMT2 levels varied greatly (Fig. 1F) in comparison to the NMT1 protein levels, which remained fairly constant (Additional file 1: Fig. S2). These observations were corroborated at the RNA level in TCGA database. Indeed, compared to normal tissues, *NMT2* expression was significantly decreased in multiple tumor types (9/11) and metastatic tissues, including breast, lung, colon, and ovarian cancers (Additional file 1: Figs. S3, S4), whereas *NMT1* expression was significantly increased in multiple tumor types (8/11) (Additional file 1: Fig. S3), and metastases (Additional file 1: Fig. S4). Although *NMT1* and *NMT2* mRNA expression levels were loosely correlated in cell lines (CCLE data; Additional file 1: Fig. S5A; *r* = 0.235, *p* < 0.0001), their protein levels were strongly correlated (Additional file 1: Fig. S5B; *r* = 0.720, *p* < 0.0001). Therefore, cells with low levels of *NMT2* are likely to have lower levels of *NMT1*.

To further characterize the importance of *NMTs* in cancer, we investigated the Dependency map [26], which aims to identify cellular vulnerabilities using CRISPR/Cas9 mediated loss-of-function screens in over a thousand human cancer cell lines. A gene effect score (Chronos) [26] based on a proliferation dynamic model post-CRISPR/Cas9 gene knockout, which accounts for copy number and other biases, was developed to evaluate the dependency on pairs of genes. The median gene effect score for non-essential genes was set to 0 while the median gene effect score for essential genes is -1. CRISPR/Cas9 KOs in 1078 cancer cell lines revealed that while *NMT2* (average gene effect score = 0.171 ± 0.003) is a non-essential gene, *NMT1* can be characterized as an essential gene in cancer cells with an average gene effect score of 0.855 ± 0.015 (Fig. 1I, J). Hematologic cancers had a higher dependency on *NMT1* (average score of -1.033 ± 0.037) than other cancer types (Additional file 1: Fig. S6). Bladder, head and neck, and colorectal cancers also exhibited higher than median *NMT1* dependency scores, with kidney cancer being the least affected by *NMT1* KO (Additional file 1: Fig. S7). Interestingly, *NMT1* dependency was significantly correlated with *NMT2* expression in all cancer cell lines (Fig. 1J; *r* = 0.600; p < 0.0001).

Finally, we evaluated the impact of *NMTs* expression on progression-free survival of DLBCL patients using publicly available datasets GSE31312 [27] and TCGA database. We found that low levels of *NMT2* or high levels of *NMT1* mRNAs were associated with poor prognosis for DLBCL patients (Fig. 1K, L, Additional file 2: Table S1). Both univariate and multivariate analyses of high versus low *NMT2* mRNA expression showed that *NMT2* mRNA levels were an independent prognostic marker for progression-free survival in the GSE31312 cohort (hazard ratio HR = 0.70 and 0.71 in univariate and multivariate analyses, respectively, Additional file 2: Table S1). The expression of *NMT2* was independent of baseline clinicopathological features (age, sex, DLBCL subtypes, and LDH; Additional file 2: Table S2). Thus, *NMT2* loss is common in lymphomas and associated with more aggressive DLBCL. Further analysis of TCGA database showed that the loss of *NMT2* is also associated with poorer patient survival in acute myeloid leukemia (AML), glioma, kidney, and ovarian cancers (Additional file 1: Fig. S8), validating the importance of *NMT2* in oncogenesis and patient outcome.

### Epigenetic silencing of *NMT2* in lymphoma cells and tumors

Since lymphoma cells and tumors showed the highest prevalence of *NMT2* loss (Fig. 1A–D), we investigated the mechanisms involved in this cancer type. To evaluate whether this loss occurred post-translationally, we treated NMT2-deficient cells with MG-132 at concentrations known to inhibit the proteasome; however, no clear recovery of NMT2 loss was observed after treatments (data not shown). We also analyzed TCGA dataset and observed that both *NMT1* and *NMT2* were only mutated at low frequencies suggesting that another mechanism was involved (data not shown).

**Fig. 2:**
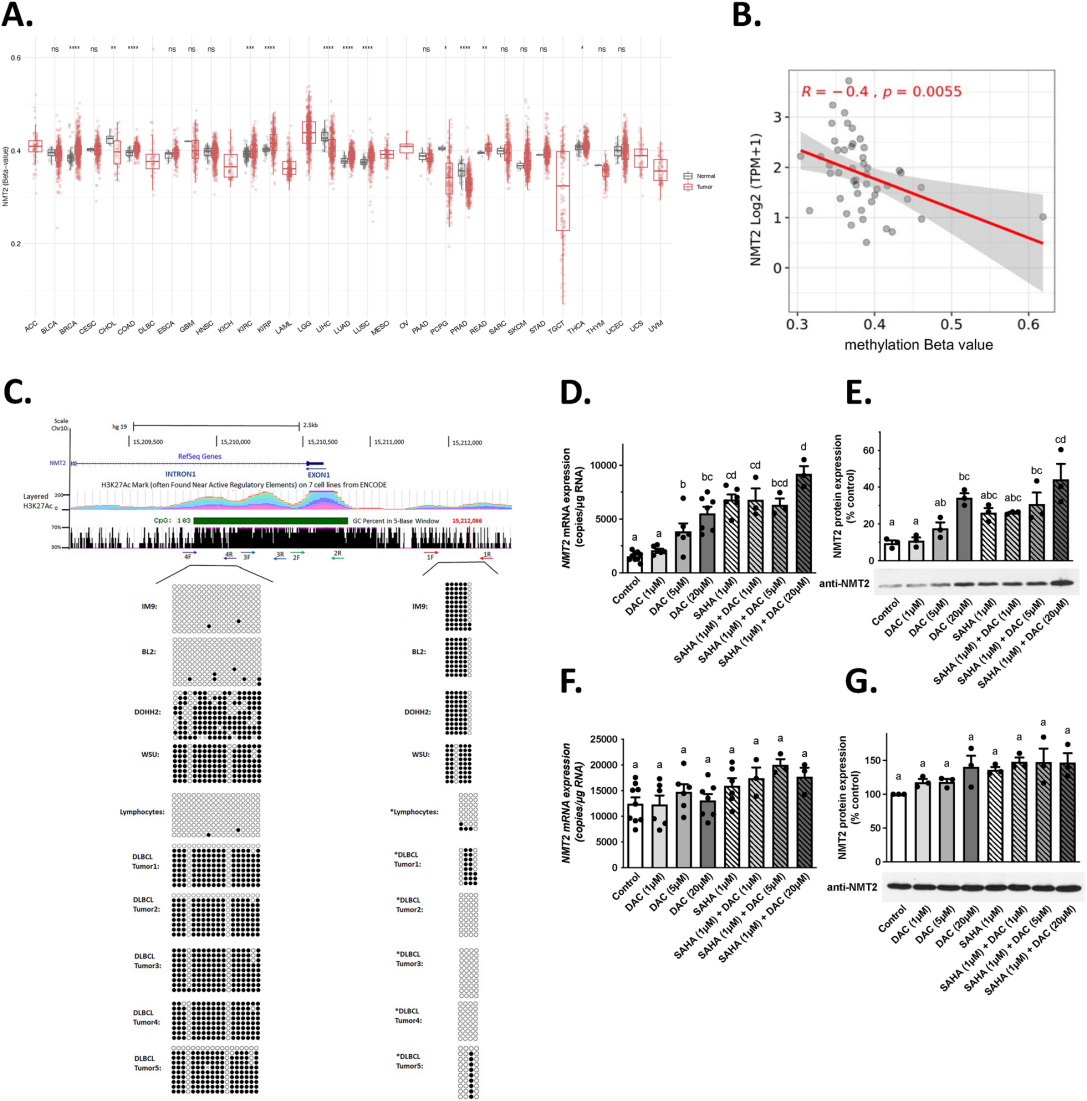
The *NMT2* locus is methylated on CpG islands in lymphoma cell lines and tumors. Aggregation methylation beta values at the *NMT2* locus in tumors and normal tissues showed increased methylation in breast, colon, kidney, and lung tumors compared to those in associated normal tissues (**A**). Aggregation methylation beta values at the *NMT2* locus in tumors correlate with *NMT2* expression in DLBCL (**B**). Structure of the *NMT2* genomic region and bisulfite sequencing results for various lymphocytic cell lines and five DLBCL patient tumors used in this study (**C**). All corresponding DLBCL tumors contained only residual levels of NMT2 by IHC (data not shown). The number of *NMT2* mRNA copies after acute 24 h treatments with DAC in the presence or absence of SAHA (1 μM) in BL2 (**D**) and IM9 (**F**). NMT2 protein levels normalized to GAPDH in BL2 (**E**) are shown as the percentage of normalized NMT2 levels in IM9 (**G**) in the corresponding treatments, as assessed by western blotting values are mean ± s.e.m. of three independent experiments. One representative western blot from three independent experiments is shown. Samples sharing letters are not statistically different (one-way ANOVA)

A common mechanism of epigenetic silencing involves histone deacetylation and DNA methylation at the CpG islands. Interestingly, when interrogating TCGA database, we found that the methylation statuses of both *NMT*s were highly regulated (Fig. 2A, Additional file 1: Fig. S9A). Aggregation methylation at the *NMT2* locus in tumors and normal tissues showed increased methylation in breast, colon, kidney, and lung tumors compared to that in the associated normal tissues (Additional file 1: Fig. S9B–E) and was significantly inversely correlated with its expression in DLBCL (Fig. 2B). In silico analyses revealed a CpG island overlapping the 5′ promoter region and the first intron of *NMT2* (Fig. 2C). Bisulfite sequencing [28] of chromosomal DNA from lymphoma cell lines and immortalized IM9 B cells confirmed that these *NMT2* loci were methylated (dark spheres) in DOHH2 and WSU-DLCL2 lymphoma cells, but not in BL2, benign IM9 cells, and lymphocytes (open circles) (Fig. 2C). To validate the methylation status of the cell lines, we used *DAPK1* as a control and found that it was highly methylated only in malignant B lymphocytes, as expected [29] (Additional file 1: Fig. S10). The CpG methylation pattern observed in lymphoma cell lines was also recapitulated in five out of five randomly selected DLBCL tumors (Fig. 2C). In patient samples, single CpG methylation at position 15,212,066 (cg02268561 beta values) showed significant inverse correlation with *NMT2* expression in DLBCL, acute myeloid leukemia (AML), and breast cancers (*p* < 0.05) and occurred in two of the five DLBCL tumors tested (Fig. 2C, Additional file 1: Fig. S9). Thus, this single methylation site could have prognostic value as it can correlate with *NMT2* expression (Patent application WO2017011907A1).

To confirm the link between epigenetic modifications and *NMT2* expression, rapidly growing lymphoma cells were treated with high concentrations of deoxyazacytidine (DAC) to inhibit DNA methylation and/or suberoylanilide hydroxamic acid (SAHA) to inhibit histone deacetylation [30] for 24 h (Fig. 2D–G). Each inhibitor resulted in time- and concentration-dependent recovery of *NMT2* mRNA levels in lymphoma cell lines, but not in the normal immortalized B cell line IM9. Combined treatment with DAC and SAHA restored *the NMT2* transcript levels in BL2 cells (Fig. 2D) as well as in DOHH2 and WSU-DLCL2 cells (Additional file 1: Fig. S11C-E) to levels similar to those observed in the untransformed IM9 cells (Fig. 2F). Paradoxically, DAC and SAHA decreased *NMT1* expression (S11A, B, D, and F), whereas *NMT2* expression increased (Fig. 2D, E) suggesting that a feedback mechanism exists between the relative expressions of these genes. In BL2 cells, the combined treatment which increased *NMT2* mRNA resulted in a fivefold increase in NMT2 protein levels, achieving 40% of those observed in untreated IM9 cells (Fig. 2E, G). However, in DOHH2 and WSU-DLCL2 cells, NMT2 was not detected by western blotting despite a significant increase in *NMT2* mRNA levels upon treatment with DAC and/or SAHA (data not shown).

### Identification of a myristoylation inhibition sensitivity signature gene set (MISS-54) from cell lines treated with myristoylation inhibitors PCLX-001 and PCLX-002

After describing the absence of *NMT2* in most hematologic cancer cell lines and tumors, and their high sensitivity towards PCLX-001, we hypothesized that NMTI could exploit this loss by targeting the remaining NMT1 in an approach reminiscent of synthetic lethality [24]. However, *NMT2* (or *NMT1*) expression levels alone did not significantly correlate with PCLX-001 EC_50_/IC_50_s in the 300 tested cell lines (Additional file 1: Fig. S12), although a weak correlation was observed for PCLX-002 EC_50_s. No significant correlation was further observed specifically for hematologic cancer cell lines (Additional file 1: Fig. S12). The expression of hundreds of genes showed stronger correlations with IC_50_s (Additional file 1: Fig. S13) than both *NMT1* and *NMT2* invalidating our initial hypothesis. Overall, although our data showed that *NMT2* loss is an important component of PCLX-001 sensitivity, cancer cell dependence on *NMT1* for survival was even more important, particularly in *NMT2*-deficient cancer cells.

**Fig. 3:**
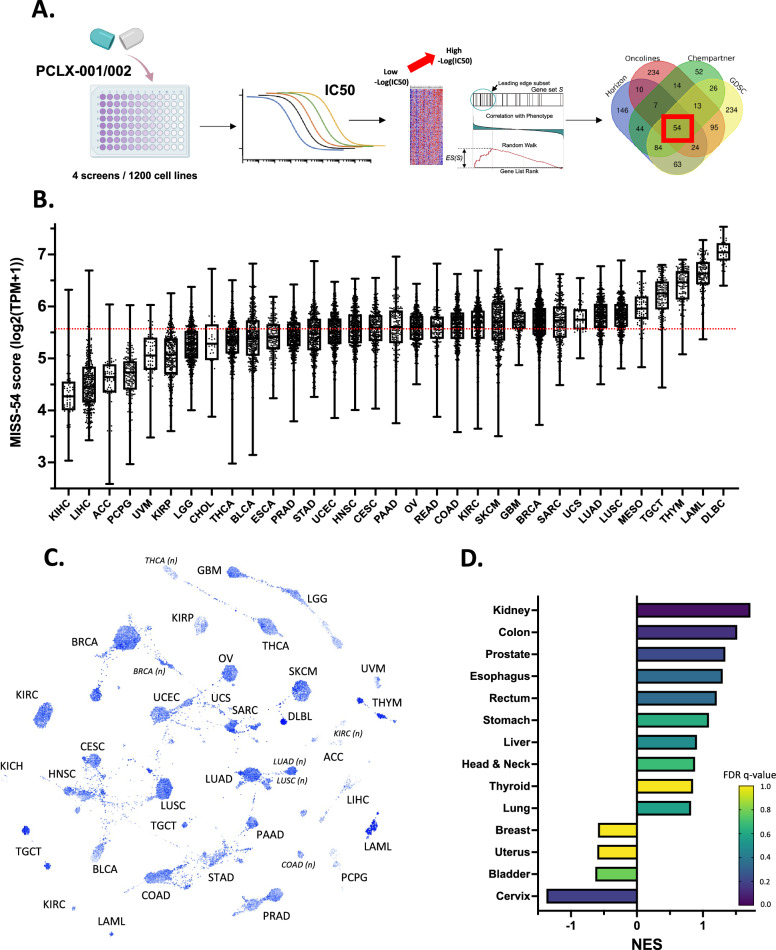
Identification of cancers susceptible to be sensitive to PCLX-001 or PCLX-002 NMTI treatment. Simplified strategy leading to the establishment of a myristoylation inhibition sensitivity signature made of 54 genes (MISS-54) (**A**) MISS-54 score in tumors sorted by cancer origin (data extracted from TCGA) (**B**). MISS-54 score (blue color scale; the darker the blue, the more sensitive the tumor) in 11,070 tissue samples (tumor and normal tissues) clustered for their total individual mRNA expression [105] (**C**). MISS-54 Normalized Enrichment Score (NES) in tumors RNA-seq expression versus their associated normal tissue (**D**)

To properly use PCLX-001 in precision medicine, we sought to identify biomarkers (other than *NMT2*) that could prospectively reflect treatment sensitivity. The sensitivity of cancer cells to PCLX-001 and PCLX-002 was evaluated in over 1200 cancer cell lines using four different screens [22, 31] (Additional file 2: Table S3). To define a myristoylation inhibition sensitivity signature gene set, we performed Hallmark Gene Set Enrichment Analysis (GSEA) to identify leading-edge genes enriched in cell lines that were the most sensitive to myristoylation inhibitors compared to the most resistant cells (Fig. 3A, Additional file 1: Fig. S14A). We used −log_10_(IC_50_) as a continuous phenotype vector for the GSEA. The Venn diagram intersection of the resulting independently identified leading-edge genes from the four screens comprised 54 genes (Additional file 2: Table S4), and defined a myristoylation inhibition sensitivity signature, MISS-54.

Multiple hallmark pathways, including allograft rejection, MYC and E2F targets, DNA repair, and inflammatory response, were enriched in myristoylation inhibitor-sensitive cells in the four screens (Additional file 1: Fig. S14B). Our previous report revealed that while hematologic cancer cell lines are extremely sensitive to myristoylation inhibition, the realm of sensitive cell lines is clearly not limited to hematologic cancers [22] (Additional file 2: Table S3). These results suggest that MISS-54 can be used to evaluate the potential sensitivity of cancer cell lines of all origins to NMTIs in silico. When we calculated the MISS-54 score in tumors from TCGA database, we found that the MISS-54 score was particularly high in DLBCL, AML, thymus, testis, lung, thymus, uterus, cervix, lung, colon, breast, and brain cancers, whereas kidney, liver, and adrenal gland cancers had below-average MISS-54 scores (median = 5.576 log2(TPM + 1)) (Fig. 3B). Cluster analysis of the various cancer-specific MISS-54 scores further revealed the landscape of tumors with high MISS-54 scores predicted to be sensitive to myristoylation inhibitor treatment (Fig. 3C, Additional file 1: Fig. S15A; darker blue indicates higher the myristoylation inhibition sensitivity score). Analysis of the MISS-54 gene set in normal and cancer tissues included in TCGA dataset revealed that MISS-54 scores were significantly higher in most cancer types than in their cognate normal tissues (Additional file 1: Fig. S15B). Kidney, colon, head and neck, and gastric cancers were predicted to have the highest differential MISS-54 score. These observations were aligned with the GTEx database, which showed a lower median score (median = 5.258 log2(TPM + 1)) for normal tissues compared with tumors (Additional file 1: Fig. S15C). Vital organs, including the liver, heart, kidney, and brain, are predicted to exhibit limited sensitivity towards NMTIs. Moreover, 72% of the GTEx normal tissues had a MISS-54 score below the median observed for tumors present in TCGA. To remove any potential bias when comparing these two datasets, we performed GSEA on normalized and corrected TCGA tumor RNA-seq expression (TCGA) data and compared the obtained scores to their cognate normal tissues (GTEx) [32]. The MISS-54 score was significantly enriched in multiple tumors compared to their cognate normal tissues, especially in colon, esophagus, lung, kidney, colon, and prostate cancers (Fig. 3D). Interrogating the CCLE database for MISS-54 also confirmed that cell lines from lymphoid and myeloid lineages had high MISS-54 scores (Additional file 1: Fig. S15D). The clinicopathological characteristics of the predicted NMTI-sensitive patients with a high MISS-54 score were female, smoking, and tumors with a high histological grade (Additional file 1: Fig. S16). Furthermore, genes included in MISS-54 were associated with poorer prognosis (overall survival and progression-free survival) in the liver, gliomas, adrenal glands, leukemia, kidney, glioma, ovarian, skin, and other cancers (Additional file 1: Fig. S17).

### *NMT* KOs markedly increase sensitivity to PCLX-001

**Fig. 4:**
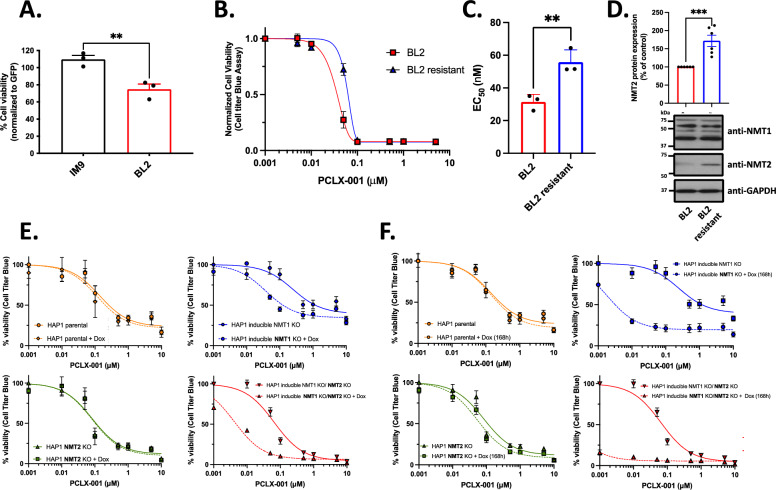
*NMT2* re-expression is toxic to NMT2-deficient cancer cells and *NMT* KO sensitizes cells to PCLX-001 treatment. NMT2-deficient cells BL2 viability after expressing *NMT2* compared to NMT2-normal IM9 cells (*p* = 0.0095) (**A**). BL2 cancer cells were grown for 6 months with increasing concentrations of PCLX-001 (from 2 to 20 nM final) to selectively isolate resistant cells. Resistant cell viability curves after treatment with increasing amounts of PCLX-001 for 96 h (*n* = 3). (**B**) Corresponding histogram of the calculated EC_50_ (*p* = 0.04); (**C**) Quantification of NMT2 protein level in BL2 resistant cells (**D**) (*p* < 0.001; ratios normalized to BL2 normal NMT2 protein level, one western blot representative of four independent experiments is shown). Viability of inducible *NMT1* KO HAP1 cells and *NMT2* KO/inducible *NMT1* KO HAP1 cells incubated with or without doxycycline (1 μg/mL) and with increasing concentration of PCLX-001 for 96 h (**E**), or pre-incubated with/without doxycycline (1 μg/mL) for 72 h then with increasing concentration of PCLX-001 for 96 h (**F**)

The highly prevalent loss of *NMT2* in various tumors suggests that it may have important functions in vivo. To gain cellular insights into *NMT2* function, we overexpressed *NMT2* in NMT2-normal IM9 and NMT2-deficient BL2 cells. The viability of co-expressing GFP-positive BL2 cells was significantly decreased by 32% (*p* = 0.0095), whereas it remained unchanged in GFP-positive NMT2-normal IM9 cells, suggesting a potential role of *NMT2* as a regulator of cell growth (Fig. 4A). To assess whether the loss of *NMT2* expression could be reversed, we grew BL2 cells in media containing increasing concentrations of PCLX-001, ranging from 2 to 20 nM, over a 6-month period [33]. By doing so, we developed PCLX-001 resistant BL2 cells with a twofold higher PCLX-001 EC_50_ of 56 nM (*p* = 0.04), which was associated with a 72% increase in NMT2 protein levels (Fig. 4B–D; similar results were obtained with the DLBCL DOHH2 cell line in Additional file 1: Fig. S18).

As reported previously [1] and shown in Fig. 1I, *NMT1* is an essential gene in both normal and cancerous cells. To investigate the role of *NMT1*, we bypassed the essential dependency of *NMT1* on cells by creating a doxycycline-inducible *NMT1* knockout system that regulates the expression of *NMT1* gRNA in near-haploid chronic myeloid leukemia (CML) HAP1 cells constitutively expressing Cas9 [34]. The validated constitutive *NMT2* KO HAP1 cells were obtained commercially. To evaluate the roles of *NMT1* and *NMT2* simultaneously, we used a doxycycline-inducible *NMT1* knockout system in *NMT2* KO HAP1 cells stably expressing Cas9. The desired indels were confirmed using DNA sequencing (Additional file 1: Fig. S19A). Effective *NMT1* KO and *NMT2* KO were confirmed using western blotting (Additional file 1: Fig. S19B). NMT1 was not detected in doxycycline-inducible cells after 72 h of incubation, whereas NMT2 was not detected in constitutive *NMT2* KO HAP1 cells.

We subjected our engineered and parental cells to increasing concentrations of PCLX-001 for 96 h in the presence or absence of doxycycline to ablate *NMT1* expression and monitored cell viability, as previously described [22] (Fig. 4E, Additional file 1: Fig. S20A). Doxycycline treatment had no effect on the viability of parental and *NMT2* KO HAP1 cells. Doxycycline treatment leading to *NMT1* ablation resulted in a sixfold reduction of the PCLX-001 IC_50_ to 38.15 nM vs 244.9 nM for non-induced cells. *NMT2* KO HAP1 cells also showed increased sensitivity (IC_50_ = 83.16 nM) compared to parental cells (IC_50_ = 137.5 nM). Doxycycline-induced double *NMT1/NMT2* KO HAP1 cells were the most sensitive to PCLX-001 treatment (IC_50_ = 4.13 nM), and their viability started to decrease rapidly after 96 h of doxycycline treatment, concomitant with our ability to detect NMT1 (Additional file 1: Fig. S19). To circumvent the latency required for gRNA^NMT1^ to fully abrogate *NMT1* expression, *NMT1* KO HAP1 cells were pretreated for 72 h with doxycycline prior to PCLX-001 treatment for 96 h (168 h with doxycycline) (Fig. 4F, Additional file 1: Fig. S20B). Doxycycline-mediated ablation of *NMT1* expression reduced PCLX-001 IC_50_ to 1.03 nM; a 250-fold increase in sensitivity. The effect of *NMT1* KO was even more significant in *NMT2* KO HAP1 cells (IC_50_ = 54 nM) after 168 h of doxycycline treatment, as the induced double *NMT* KO cell viability dropped to 16% without PCLX-001 treatment, demonstrating that *NMT1* is more important for the viability of *NMT2* deficient cells (Fig. 4F). Our results confirm the requirement of *NMT1* for cell survival, and genetically validated the pharmacological targeting of *NMT*s with an NMTI for cancer therapy, especially in cancers with a highly prevalent loss of *NMT2*.

### Genetic or pharmacological inhibition of myristoylation reduces mitochondrial respiratory complex I proteins and OXPHOS, in addition to reducing cell signaling

**Fig. 5:**
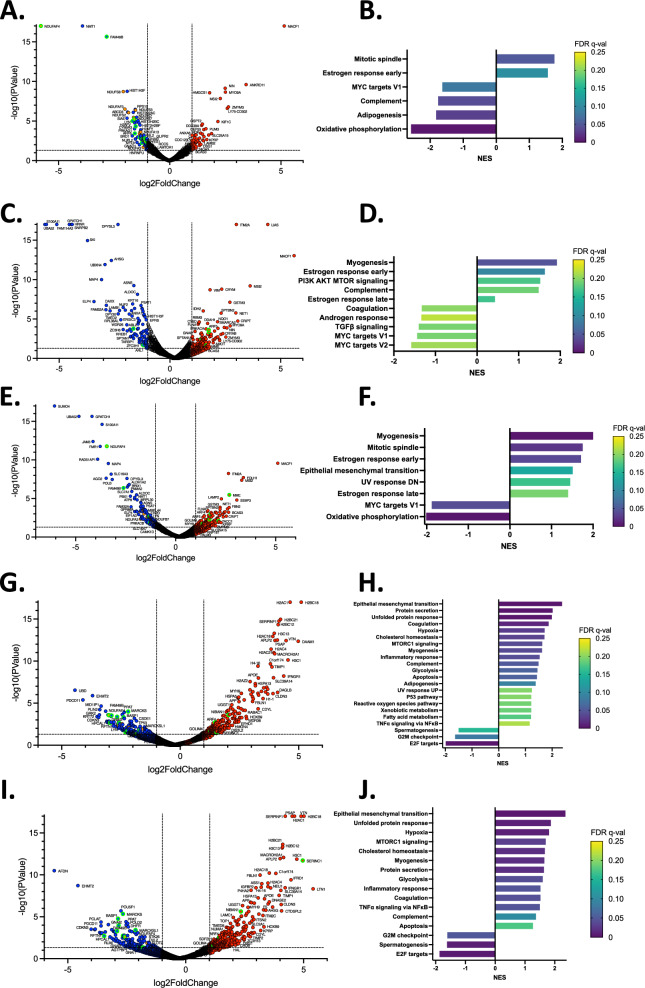
Genetic and pharmacologic myristoylation inhibition drastically changes the proteomes of HAP1 cells. Differential proteomic analysis of inducible *NMT1* KO HAP1 cells treated with doxycycline (96 h) (myristoylated proteins bordered in green and complex I proteins shown in orange) (**A**), *NMT2* constitutive KO (**C**), and inducible *NMT1* KO/*NMT2* KO (**E**) with the Hallmark Gene Set Enrichment Analysis of the proteins decreased and increased (False Discovery Rate qVal < 0.25) (**B-D-F**). Proteomic analysis of inducible parental HAP1 cells treated with PCLX-001 at 0.5 (**G**) or 5 μM (**I**) for 72 h (myristoylated proteins bordered in green) with associated Hallmark Gene Set Enrichment Analysis of the proteins decreased and increased in HAP1 cells (False Discovery Rate qVal < 0.25) (**H–J**)

Since proteins exposing an un-myristoylated N-terminal glycine residue are degraded [19], we performed differential proteomics in HAP1 cells deficient in *NMT1*, *NMT2*, or both, to identify myristoylated and non-myristoylated proteins affected by the loss of either or both *NMT genes* in our engineered *NMT* KO cells. When we compared the proteomes of doxycycline-induced *NMT1* KO HAP1 cells with those of non-induced cells, we identified 108 significantly downregulated proteins (Fig. 5A). Surprisingly, very few signaling proteins, other than Lyn SFK, were downregulated in an *NMT1* specific manner. Rather, the major effect of *NMT1* KO was on the mitochondrial proteins. Specifically, 36 mitochondrial proteins were downregulated, 20 of which belonged to mitochondrial complex I. Of these, the myristoylated mitochondrial complex I assembly factor protein NADH dehydrogenase 1 alpha subcomplex assembly factor 4 (NDUFAF4) was the most downregulated (Fig. 5A, top left corner), followed by myristoylated complex I-associated protein NDUFB7. Enrichment analysis also confirmed that oxidative phosphorylation (OXPHOS) was the pathway most affected by *NMT1* KO, along with adipogenesis, the complement system, and the MYC signaling pathway (Fig. 5B). *NMT2* KO mainly decreased the levels of proteins involved in the MYC signaling pathway (Fig. 5C, D), but had no effect on OXPHOS, suggesting that the effects on respiratory complex I and OXPHOS were specific to *NMT1*. The pathways affected by the double KO were a combination of the two individual *NMT* KOs with both OXPHOS and the MYC pathway downregulated (Fig. 5E, F). Both *NMT1* and *NMT2* KOs caused an increase in numerous protein levels. 75 proteins were upregulated in *NMT1* KO cells and were enriched in the mitotic spindle and estrogen response pathways, whereas 181 proteins were upregulated in *NMT2* KO cells and were enriched in myogenesis, estrogen response, and PI3K/AKT/MTOR signaling pathways.

Proteomic analysis of HAP1 parental cells, whose myristoylation was pharmacologically inhibited by PCLX-001, indicated that these cells responded differently than cells subjected to double genetic ablation of *NMT* expression (Fig. 5G–J, Additional file 1: Fig. S21). While the most similar downregulated proteins between the two datasets were myristoylated proteins (*e.g.* NDUFAF4, Lyn, and FAM49B), only a few non-myristoylated proteins were downregulated or upregulated in both datasets (Additional file 1: Fig. S21C, S22). This might be explained by incomplete *NMT1* KO. Enrichment analysis revealed that proteins involved in the response to cellular stress were significantly increased, including unfolded protein response, hypoxia, protein secretion, and apoptosis in cells treated with PCLX-001 (Fig. 5H, J). Intriguingly, seven of the top 12 upregulated genes in PCLX-001 treated non-induced or parental cells (Fig. 5G, I), are histone variants. However, this has yet to be explained. In addition, it is interesting to note that enrichment analysis revealed that the expression of glycolysis genes, including the GLUT1 main glucose transporter (*SLC2A1* gene) (Fig. 5I), increased in response to PCLX-001, perhaps as a consequence of diminished OXPHOS in PCLX-001 treated HAP1 cells.

Next, we examined the transcriptomes of these cells using RNA-Seq. Our analyses confirmed that *NMT1* KO predominantly affected the OXPHOS and MYC signaling pathways (Additional file 1: Fig. S22). Although the RNAs of the OXPHOS and MYC signaling pathways were upregulated in *NMT1* KO cells, the corresponding protein levels were not (Fig. 5A, Additional file 1: Fig. S22), perhaps reflecting a yet to be identified homeostatic compensatory mechanism that links the increased proteolytic degradation of the altered proteins to their increased transcription. The RNAs from *NMT2* KO and *NMT1/NMT2* double KO HAP1 cells were enriched in interferon alpha and gamma responses while those from HAP1 cells treated with PCLX-001 at 0.5 or 5.0 μM were both enriched in TNFα signaling via NFκB, protein secretion and unfolded protein response. Altogether, our data suggest that in addition to impacting cell signaling at the plasma membrane, as seen in lymphoma cells [22], the major effects of *NMT* KOs or PCLX-001 treatment are to drastically reduce mitochondrial respiratory complex I proteins.

**Fig. 6:**
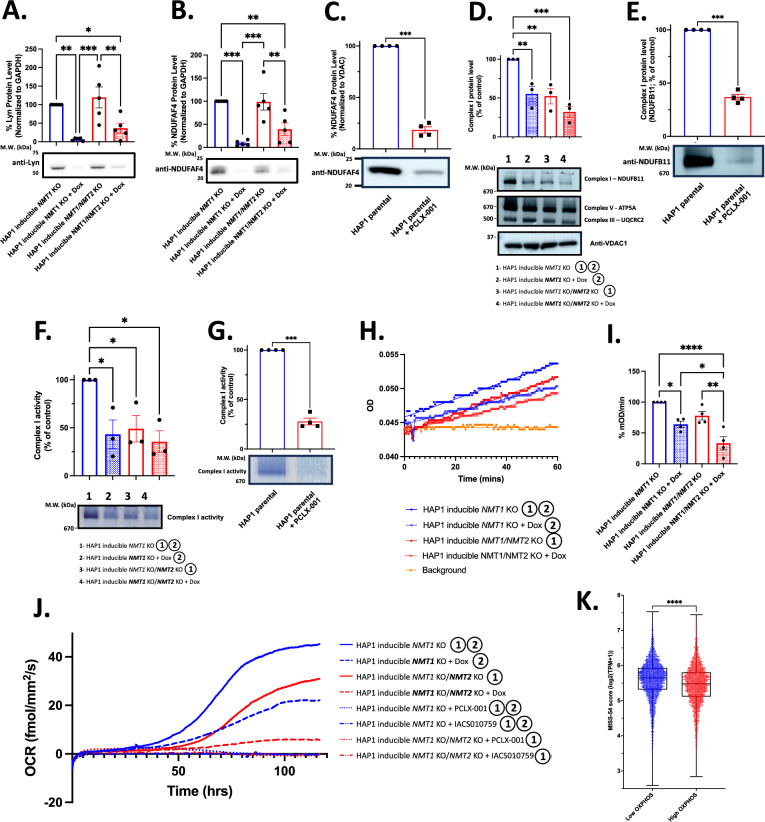
Doxycycline-induced *NMT1* KO and PCLX-001 treatment lead to complex I mis-assembly and degradation, reduced activity, and inhibition of cellular oxygen consumption rate. Western blot analysis of SFK member Lyn (**A**) and NDUFAF4 in HAP1 inducible *NMT1* KO and inducible *NMT1/NMT2* KO cells treated with or without doxycycline (**B**) (1 μg/ml) for 72 h or treated with PCLX-001 (1 μM) for 48 h (**C**). Complex I (NDUFB11), complex V (ATP5A), and complex III (UQCRC2) Blue Native PAGE [106], immunoblotting and quantification for HAP1 inducible *NMT1* KO and inducible *NMT1/NMT2* KO treated with or without doxycycline (1 μg/mL) for 72 h (**D**) or with PCLX-001 (1 μM) for 48 h (**E**). In gel complex I diaphorase activity assay of isolated mitochondria from HAP1 inducible *NMT1* KO and inducible *NMT1/NMT2* KO treated with/without doxycycline (1 μg/mL) for 72 h (F) or with PCLX-001 001 (1 μM) for 48 h (**G**). Microplate-based complex I diaphorase activity assay of total cell lysates from HAP1 inducible *NMT1* KO and inducible *NMT1/NMT2* KO cells treated with or without doxycycline (1 μg/mL) for 72 h (**H**) and quantification of four independent experiments (**I**). Total oxygen consumption of HAP1 inducible *NMT1* KO and inducible *NMT1/NMT2* KO treated with/without doxycycline (1 μg/mL), PCLX-001 (0.5 µM) or IACS10759 (1 µM) was measured over time using a Resipher cell culture monitor (**J**). MISS-54 scores of tumors with high and low OXPHOS scores (**K**) [36]

To confirm some of our proteomic data, we first demonstrated that both Lyn SFK and mitochondrial complex I assembly factor NDUFAF4 were both degraded in HAP1 cells, specifically in the absence of *NMT1* (Fig. 6A, B) or after PCLX-001 treatment (Fig. 6C) [22]. In addition, we confirmed that NDUFAF4 is required for complex I assembly and optimal activity in CRISPR/Cas9 *NDUFAF4* KO HAP1 cells (Additional file 1: Fig. S23). Therefore, we next investigated the impact of *NMT* KOs on the assembly of complex I in isolated mitochondria. *NMT1* KO, *NMT2* KO, and inducible *NMT1/NMT2* double KOs significantly decreased the amount of complex I visualized by native PAGE at approximately 950 kDa and as monitored by the reduction in NDUFB11 complex I-specific subunit level (Fig. 6D). This resulted in an overall decrease in in-gel native complex I activity (Fig. 6F), and decreased complex I-dependent diaphorase enzymatic activity in permeabilized cells (Fig. 6H, I). PCLX-001 treatment also resulted in a significant reduction in complex I assembly (Fig. 6E) and activity (Fig. 6G).

Finally, we confirmed the impact of the various *NMT* KOs and PCLX-001 on oxygen consumption, which is required for ATP production via OXPHOS in our cells. During *NMT1* KO induction with doxycycline, the oxygen consumption rate (OCR) of cells progressively decreased over time (Fig. 6J, Additional file 1: Fig. S24). As seen above, the impact of KOs was greater in double *NMT* KO than in *NMT1* KO > *NMT2* KO. Importantly, the treatment of doxycycline-induced or non-induced *NMT* KO HAP1 cells or parental cell lines with PCLX-001 resulted in a significant reduction in OCR within 48 h and a total loss of oxygen consumption within 72 h (Additional file 1: Fig. S24). As expected, oxygen consumption was also completely blocked in HAP1 cells treated with the complex I inhibitor IACS-010759 [35].

Since mitochondrial complex I is required for the proper electron transport system (ETS), OXPHOS, and thus energy production, we evaluated the impact of *NMT1* KO on the metabolomes of HAP1 cells and culture media. *NMT1* KO led to an enrichment in metabolites of the pentose-phosphate pathway, glycerolipids, valine, leucine, and isoleucine degradation, pyrimidine metabolism, glycolysis/gluconeogenesis, and pyruvate metabolism in HAP1 cells at day 3, with similar pathways affected at day 5 in both, cells (Additional file 1: Fig. S25), and culture media (Additional file 1: Fig. S26). Recent studies have investigated tumor metabolic reprogramming and the roles of OXPHOS and glycolysis in cancer cells [36]. Tumors were characterized by the expression of glycolytic and OXPHOS pathway genes. As myristoylation inhibition drastically affected OXPHOS and metabolism, we finally investigated whether the metabolic profiles of tumors could be related to potential NMTI sensitivity as assessed using MISS-54. Tumors with low OXPHOS scores had significantly higher MISS-54 scores than tumors relying on the highly expressed OXPHOS machinery, whereas no predicted sensitivity difference was found based on their glycolytic activity status (Fig. 6K, Additional file 1: Fig. S27).

## Discussion

### *NMT2* loss due to epigenetic suppression partially contributes to PCLX-001 sensitivity and is linked to poor prognosis

Prompted by the highly prevalent loss of *NMT2* expression in various cancers (Fig. 1), especially in hematologic cancers, we sought to target the remaining NMT1 with the potent pan-NMT inhibitor PCLX-001 in a manner reminiscent of synthetic lethality, thereby sparing normal cells with two NMTs. We now demonstrate that *NMT2* loss is linked to poorer prognosis in DLBCL and that *NMT2* suppression occurs via hypermethylation of a CpG island found at the 5’ end of the *NMT2* gene in hematologic and solid cancer tumors. When *NMT2* expression was restored in BL2 NMT2-deficient cell lines, we observed a decreased viability of transfected cells (Fig. 4A), suggesting *that NMT2* may have growth-inhibitory function. Consequently, the loss of *NMT2* could help drive tumor development.

Importantly, our analyses of DepMap dataset demonstrated that in the absence of *NMT1*, *NMT2* was found to be the most important survival factor (Fig. 1J) and the most influential parameter (41.7%) for *NMT1* dependency. The *NMT1* KO dependency and *NMT2* expression pair represents the 20th strongest interaction between the dependency of a given gene and the expression of a different gene within DepMap [26]. Furthermore, NMT2 levels positively correlated with NMT1 levels. Therefore, cancer cells low in NMT2 are likely to be low in NMT1 and possibly easier to eliminate pharmacologically because they have fewer enzymatic targets than normal cells. Overall, while *NMT2* loss initially appeared to be a significant component of NMTI sensitivity, cancer cell dependency on *NMT1* for survival is even more important (Fig. 4E, F), particularly in *NMT2*-deficient cancer cells (Fig. 1J). This suggests that other factors may explain the enhanced killing of select cancer cell types by PCLX-001, the first NMTI being tested in human clinical trial to evaluate its safety and tolerability in patients [21] (https://clinicaltrials.gov/ct2/show/NCT04836195) (see below).

### Development of a myristoylation inhibition sensitivity signature (MISS-54)

Since *NMT2* expression alone could not explain the spectrum of cancer cell vulnerability to NMTIs, we developed a 54 gene set named MISS-54 reflecting the expression profile of cells sensitive to PCLX-001 or PCLX-002 in comparison to resistant cells. These genes are found in multiple hallmark pathways (allograft rejection, MYC targets, E2F targets, etc.) that have been associated with cancer initiation and progression [37–39], especially in hematologic malignancies [40]. It is important to mention that we were unable to identify a gene set signature for resistant cell lines using ascending GSEA when the same methodology was applied (data not shown). This suggests that there is a promising potential for myristoylation inhibition therapy as there might be very limited resistance mechanisms available to bypass it as confirmed in Fig. 4D and Additional file 1: Fig. S18.

When MISS-54 was used to create a score based on RNA-Seq expression [41], we identified numerous cancers with tumors expression predicting their potential sensitivity to myristoylation inhibitors, including hematologic cancers DLBCL and AML, as expected from our preclinical data [22], solid tumors of the thymus (THYM), testis (TGCT), mesothelium (MESO), lung (LUSC and LUAD), uterus (UCS), breast (BRCA), brain (GBM), colon (COAD), rectum (READ), ovary (OV), pancreas (PAAD), cervix (CESC), and head and neck (HNSC) (Fig. 3B, C). Tumor clustering based on the expression of the 54 genes composing MISS-54 was only maintained for highly sensitive and resistant tumors, whereas the rest of the tumor distribution was more diffuse (Additional file 1: Fig. S28). This suggests that the predicted sensitivity is not exclusive to a specific type of cancer and is more reliant on common signaling and metabolic features. This also suggests that MISS-54 is not limited to a hematologic cancer signature, as cancers of other origins are also clustered.

A similar sensitivity trend was previously observed by Li et al., in which ICL1100013, also known as DDD85646 or PCLX-002, was predicted to be more efficient against DLBCL, HNSC, TGCT, and THYM [42]. In addition, high-scoring MISS-54 hematologic, testicular, and colon cancers were among the cancers most dependent on *NMT1* in DepMap (Additional file 1: Fig. S7) while kidney cancer was *NMT1*-independant and had the lowest MISS-54 score. These similarities are consistent with the sensitivity predictions resulting from MISS-54. As it stands, MISS-54 score is not predictive of patient response but a molecular tool to prioritize cancer indications likely to respond based on expression profiles. It also functions as a baseline for future development and validation of a predictive response score in combination with future clinical data.

Furthermore, because patients with lung, breast, uterine, or ovarian tumors have a significantly higher MISS-54 score, we found that smokers as well as females were predicted to be more responsive to PCLX-001 treatment (Additional file 1: Fig. S16). It is also worthy to notice that Epstein–Barr virus (EBV)-infected cells displayed elevated MISS-54 scores (Additional file 1: Fig. S15C), which may be due to transcriptional reprogramming leading to increased RNA synthesis, metabolism (mitochondrial activity), and cell division that occurs during infection as observed in human B-lymphocytes [43]. This observation warrants the investigation of the effects of PCLX-001 on EBV- and potentially other virus-transformed cells.

Interestingly, our Over-Representation Analysis (ORA; Additional file 1: Fig. S29) demonstrated that MISS-54 proteins are associated with ribosomes, sites of co-translational N-myristoylation, and membranes, where most of the newly myristoylated proteins are targeted. Notably, “establishment of protein localization to membrane” and “protein targeting” gene sets were also associated with MISS-54, further validating MISS-54 as a relevant set of markers of NMTIs sensitivity. This analysis also confirmed the over-representation of these 54 genes in lymphoma, leukemia as well as in infection and immune disorders (Additional file 1: Fig. S29D) and suggests a potential use for PCLX-001 in these indications.

The functions of proteins included in MISS-54 are mostly associated with RNA-related processes. RNA-binding proteins and ribosomal proteins (RP) (11 RP in MISS-54) have been associated with tumor progression [44] through their regulatory functions in the cell cycle, DNA repair, and apoptosis [45]. *MYC* is also a major regulator of ribosome biogenesis [46], and is included in MISS-54 (Additional file 2: Table S4). *MYC* is involved in the deregulation of most hallmarks of cancer (proliferation, differentiation, apoptosis, metabolism, immune surveillance, and angiogenesis) [38]. Recently, a gene signature that correlated with *MYC* expression and was significantly enriched in ribosome biogenesis and RNA metabolism genes was identified in *MYC*-driven cancer [47], further confirming the importance of ribosomal regulation in *MYC-specific* oncogenic properties. *MYC* also profoundly regulates cancer cell metabolism [48], mitochondrial biogenesis, and enzymes involved in glycolysis and OXPHOS [49]. *MYC* KO led to a rapid decrease in mitochondrial mass, structural integrity, and function, along with the degradation of respiratory complex I [50], suggesting a higher OXPHOS dependency in *MYC*-driven cancer cells. This observation is especially relevant to our study as PCLX-001 also disrupts respiratory complex I, thereby creating a new opportunity to kill cancer cells, particularly those with low OXPHOS machinery expression (as predicted in Fig. 6K) and *MYC*-driven cancers.

Interestingly, cancer cells that were low in *NMT2*, highly dependent on *NMT1,* or predicted to be sensitive to NMTI treatment (high MISS-54 score) showed high *MYC* expression (Additional file 1: Fig. S30). Since *MYC-driven* cancers [38] and high MISS-54 scored patients (which include high *MYC* expressors; Additional file 2: Table S4) have low therapeutic success and poorer survival (Additional file 1: Fig. S8), PCLX-001 treatment could potentially offer therapeutic benefits for these often heavily treated patients, although this will require to be validated in clinical trials.

### In addition to inhibiting cell signaling, *NMT1* KO and PCLX-001 inhibited mitochondrial complex I, OXPHOS, OCR, and altered cellular metabolism

Genetic ablation of *NMT1* or both *NMTs* significantly increased the sensitivity to PCLX-001, suggesting that cells containing less NMT are more vulnerable to NMT inhibition. Moreover, our data show that the ablation of both *NMTs* is lethal for cells, demonstrating that NMTs are *bona fide* cancer targets. This latter point was recently inferred by others who genetically interfered with *NMT* expression in tumors and demonstrated reduced liver and bladder tumor growth [17, 51].

When we knocked out *NMT1* in HAP1 cells or treated these cells with PCLX-001, we found that mitochondrial respiratory proteins were markedly downregulated (Fig. 5). The loss of myristoylation in these cells, either by genetic ablation or pharmacological inhibition of NMTs, resulted in the destruction of complex I, loss of OXPHOS, and abrogation of OCR (Fig. 6, Additional file 1: Fig. S24), which led to changes in metabolism as the cells attempted to adapt to the loss of OXPHOS capacity and reduced ATP production. Enrichment analysis confirmed that hypoxia [52] and glycolysis pathways were upregulated in response to PCLX-001 treatment. Loss of OXPHOS also resulted in an increase in glycerolipid and glycolytic/gluconeogenesis metabolites (Additional file 1: Figs. S25, S26). As a consequence of decreased OXPHOS, cells might adapt by reducing their use of fatty acids as β-oxidation fuel, and these could accumulate as glycerolipids in lipid droplets. Similarly, cells likely enhanced their use of glycolysis or gluconeogenesis to adapt to reduced OXPHOS. The metabolic switch from OXPHOS to glycolysis observed in most cancer cells is controlled by the metabolic sensor AMPK [53], which is myristoylated [54]. The association of AMPK with mitochondria is dependent on its β-subunit myristoylation [55] which is necessary for mitochondrial removal. In the presence of PCLX-001, tumor cells that are unable to switch from defective OXPHOS to glycolysis owing to non-functional AMPK could consequently readily die.

As shown previously [22, 56], PCLX-001 treatment leads to activation of the ER stress response pathway and apoptosis. Our proteomic analysis confirmed that several cellular stress response pathways, such as the unfolded protein response (UPR), reactive oxygen species, P53, hypoxia, and apoptosis pathways, were significantly enriched (Fig. 5H, J). UPR serves as an ER stress sensor that induces apoptosis following prolonged, acute, and irreversible stimuli [57]. The observed mis-assembly and degradation of complex I could thus contribute to additional stress, resulting in increased ROS [58] if complex I is only misassembled, or decreased ROS production in the event that complex I is completely degraded. This could lead to the possible transcriptional activation of genes participating in both the response to low oxygen levels (hypoxia) and the activation of the p53 pathway [59]; however, further investigation is required to fully understand the pathways involved.

Importantly, *NMT1* KO or PCLX-001 could also have other indirect effects on mitochondrial metabolism via inhibition of additional myristoylated mitochondrial proteins, including MIC19, MIC25, SAM50, and TOMM40 [60], which are responsible for mitochondrial protein import and cristae structure [61]. Notably, the cristae structure is critical for proper ETS function and OXPHOS [62]. Numerous reports have demonstrated the presence of SFKs in the mitochondria, where they have often been postulated to regulate OXPHOS [63–65]. Therefore, pharmacological loss of SFKs not only impairs their functions at the plasma membrane, but could also represent a novel way to detrimentally impact mitochondrial homeostasis, in particular OXPHOS, and increase cancer cell susceptibility to PCLX-001.

While our report newly demonstrates that one of the major effect of *NMT1* KO and PCLX-001 treatment is on mitochondrial proteins, we showed that NMT inhibition also profoundly affected numerous myristoylated signaling proteins in addition to SFKs, including oncogenes (ABL2 [66], MARCKS [67], BASP1 [68]), membrane lipid raft proteins including flotillin-2 [69] and raftlin [70], and proteins involved in vesicular transport (e.g., Arf1-6) [71] (Fig. 4). Importantly, while numerous SFK inhibitors have been developed for the treatment of hematologic [72, 73] and solid tumors [74–76], these often lead to target stabilization and accumulation of potentially hazardous proto-oncogenic proteins [77]. By preventing SFK myristoylation and promoting their proteolytic degradation (and that of other myristoylated signaling proteins) [19], PCLX-001 represents an alternative approach for targeting SFKs and signal transduction from RTKs at the source in a novel and quasi-permanent manner, thereby impacting cancer cell growth.

### PCLX-001 mediated loss of OXPHOS may target metastatic cells and cancer stem cells

Although the Warburg effect shows that most cancer cells preferentially use aerobic glycolysis to provide ATP and key metabolites [78], this effect is maintained by hypoxia-inducible factor-1α (HIF-1α) [79] which requires NAD+/NADH balance produced by complex I. This balance is also required for nucleic acid synthesis for DNA replication and cancer cell proliferation further highlighting the key role of complex I in these processes. Cancer cells are well known to reprogram their metabolism. Increasing evidence suggests cancer cells can switch between glycolysis and OXPHOS, a phenomenon denoted as cancer cell metabolic plasticity, and maintain both reprogrammed and oxidative metabolism [80, 81]. Such oxidative metabolism, including OXPHOS, is required by metastatic cancer cells [82]. Furthermore, these cells possess a distinct metabolic profile enriched in mitochondrial respiratory complex I proteins [83] (including NDUFAF4), making them possibly sensitive to OXPHOS inhibition. Similarly, cancer stem cells (CSCs) have been shown to have increased mitochondrial biogenesis [84] and use OXPHOS as their main source of energy in leukemia [85, 86] as well as breast and lung cancers [87, 88]. Several drugs targeting mitochondrial function, including the complex I inhibitor IACS-010759, have shown some promise in vitro for killing CSCs [35] and have been tested in clinical trials [89]. Therefore, while it may seem counterintuitive, complex I activity thus helps maintain this metabolic plasticity and its disruption may be detrimental to cancer cells, more so than normal cells. While additional studies are necessary, indirectly targeting complex I and/or OXPHOS with compounds such as PCLX-001/zelenirstat appears as an attractive new approach to possibly reduce metastasis and contribute to the eradication of CSC-mediated relapses [90, 91].

## Conclusion

**Fig. 7:**
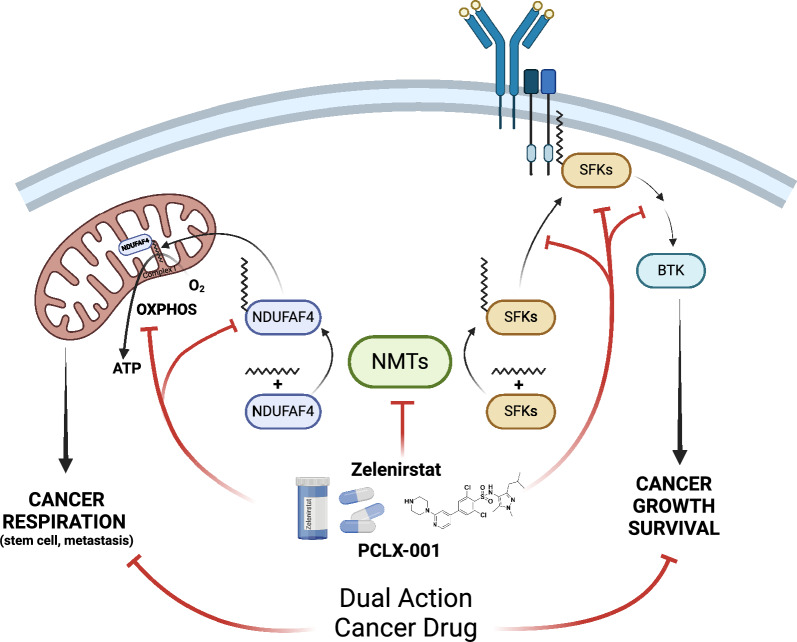
PCLX-001 is a dual-action drug that targets pro-survival signaling and OXPHOS/respiration in cancer cells. PCLX-001 (zelenirstat) is the first NMTI tested in humans. By inhibiting both NMTs, PCLX-001 not only induces the degradation of multiple substrates, including proto-oncogenic Src family kinases, essential for receptor tyrosine kinases (RTK) survival signaling [22] but also abrogates the function of NDUFAF4, an essential assembly factor for the respiratory complex I, by promoting its degradation. Loss of complex I activity leads to loss of OXPHOS and respiration of cancer cells. Since OXPHOS is required for metastasis and cancer stem cell survival, the two major causes of death and relapse from cancer, this makes PCLX-001/zelenirstat an attractive dual acting drug prospect for the treatment of cancers

Collectively, our epigenetic, proteomic, metabolomic, and transcriptomic analyses revealed that numerous factors contribute to the sensitivity of cancer cells to myristoylation inhibition, including the loss of *NMT2*, dependency on *NMT1*, and increased expression of a gene set encompassing 54 genes that we named MISS-54. This new expression signature will inform the development of PCLX-001 by suggesting new therapeutic indications for evaluation and perhaps lead to the identification of patients most likely to respond to PCLX-001 once clinically validated. Because of the number of potential direct and indirect targets of NMTIs (2 NMTs myristoylating over 200 proteins), an increased effort to reveal the mechanism(s) of action of this new family of drugs is required. Herein, we are now demonstrating that PCLX-001 (zelenirstat) is a dual-action drug that can efficiently and specifically target cancer cell by inhibiting their pro-survival signaling and abrogating mitochondrial respiration (see model Fig. 7). The safety, tolerability, cancer specificity and pharmacokinetics of PCLX-001/zelenirstat were evaluated in a phase 1 dose-escalation study in patients with relapsed/refractory B-cell lymphoma and advanced solid malignancies [21], which is now completed (Manuscript submitted for publication). Our phase 1 results and results herein warrant further clinical evaluation of PCLX-001/zelenirstat in hematologic and solid tumors.

## Materials and methods

### Antibodies and materials

Rabbit polyclonal anti-NMT1 (1:2000) was purchased from Proteintech (cat#11546-1-AP) and mouse monoclonal anti-NMT2 (1:2000) was purchased from BD Biosciences (clone 30; cat#611310). Rabbit anti-GAPDH (1:20,000) was obtained from the laboratory stock and is available through Eusera (www.eusera.com). Rabbit monoclonal anti-Lyn (clone C13F9, cat#2796) was purchased from Cell Signaling Technology (Danvers, MA, USA). Rabbit anti-Mcl-1 (cat#Ab32087), rabbit anti-VDAC1 (ab154856), rabbit NDUFB11 (cat#ab183716), and Total OXPHOS rodent cocktail (cat#ab110413) were purchased from Abcam (Cambridge, MA, USA). Rabbit anti NDUFAF4 (cat#A14345) was purchased from Abclonal (Woburn, MA, USA). Mouse monoclonal NMT1 (6F8D5) (1:500) and NMT2 (6C5E8) (1:1000) were developed and validated in our laboratory [23]. Unlike the commercial antibody from Proteintech, which detected the 4 NMT1 isoforms [9, 92], monoclonal 6F8D5 only detected a unique NMT1 band. SuperSignal West Pico PLUS Chemiluminescent substrate was purchased from Thermo Scientific (Rockford, IL, USA).

All chemicals were of the highest purity available and were purchased from Sigma-Aldrich unless otherwise indicated.

### Datasets

Cancer Cell Line Encyclopedia (CCLE) RNA-seq expression data and dependency scores were obtained from the Depmap [93] portal in February 2023 (22Q4). Microarray expression and clinical data were retrieved in April 2014 for patients with lymphoma profiled using the Gene Expression Omnibus (GEO) series, GSE31312.

TCGA data were prepared using R version 4.2.2. Data were downloaded directly from TCGA using TCGA Biolinks package [94] (ver. 2.26.0). Unstranded TPM counts were extracted for each of the 54 genes in the MISS-54 score for 11,274 tumors and normal tissues. Code, input data, example intermediates, final output, and a full list of packages, including versions, are available upon reasonable request.

### Cell culture

IM9 was purchased from ATCC. BL2 was a kind gift from Dr. Karl Riobowol (U. of Calgary), Dr. Jim Stone, and Dr. Robert Ingham of the University of Alberta. DOHH2 were purchased from DSMZ (Germany) Cell line identity was confirmed at The Genetic Analysis Facility, The Centre for Applied Genomics, The Hospital for Sick Children, Peter Gilgan Centre for Research and Learning, 686 Bay St., Toronto, ON, Canada M5G 0A4 (www.tcag.ca). HAP1 parental (#C631) and NMT2 KO (#HZGHC000562c006) cells were purchased from Horizon (https://horizondiscovery.com). The cells were maintained in RPMI or IMDM medium supplemented with 5–10% fetal bovine serum, 100 U/mL penicillin, 0.1 mg/mL streptomycin. All cell lines were maintained at 37 °C and 5% CO_2_ in a humidified incubator, and routinely checked for the presence of contaminating mycoplasmas.

BL2 and DOHH2 PCLX-001 resistant cells were selected by incubating the cells with increasing concentrations of PCLX-001 for more than 6 months. The starting concentration was 2 nM and the cells were viable (over 90% viability) at 20 nM at the end of the selection.

### Lysis of cells and lymphoma tumor samples

Cells were harvested, washed in cold PBS, and lysed in 0.1% SDS-RIPA buffer (50 mM Tris–HCl pH 8.0, 150 mM NaCl, 1% Igepal CA-630, 0.5% sodium deoxycholate, 2 mM MgCl_2_, and 2 mM EDTA with 1× complete protease inhibitor; Roche Diagnostics) by rocking for 15 min at 4 °C. The lysates were centrifuged at 16,000 × g for 10 min at 4 °C and the post-nuclear supernatant was collected.

Human diffuse large B-cell lymphoma (DLBCL) and follicular lymphoma (FL) tissues were obtained from patients consented by Dr. Raymond Lai (Cross Cancer Institute, Alberta, Canada) from consented patients. Frozen tumor tissues were cut into ~ 1-mm^3^ pieces and mixed with 1% SDS-RIPA with 1× complete protease inhibitor. Samples were homogenized with a small Dounce homogenizer and sonicated repeatedly for 2 min at 1-min intervals (on ice) at an output of 6.0 (Branson Sonifier 450) until the tumor tissues dissolved in the lysis buffer. The samples were then centrifuged at 16,000 × g for 10 min at 44 °C and the post-nuclear supernatant was collected for western blot analysis.

### Immunoblotting

Protein concentrations were determined using the BCA assay (Thermo Scientific), according to the manufacturer’s instructions. Samples were prepared for electrophoresis by adding 5× loading buffer and boiling for 5 min. If not stated otherwise, 30 μg of total protein per lane was loaded onto a 12.5% acrylamide gel. After electrophoresis, gels were transferred onto a 0.2 μM nitrocellulose membrane (Bio-Rad) and probed with antibodies as described in the Materials section. Peroxidase activity is revealed following the procedure provided for the ECL Prime Western Blotting Detection Reagent (GE Healthcare, PA, USA).

### Immunohistochemistry

B-cell lymphomas were fixed in formalin, embedded in paraffin, cut into 5 mm sections with a microtome, mounted on Superfrost Plus slides (Fisher Scientific), deparaffinized with xylene (3 times for 10 min each), dehydrated in a graded series of ethanol (100%, 80%, and 50%), and washed in running cold water for 10 min.

For antigen retrieval, the slides were loaded into a slide holder and placed in a Nordicware microwave pressure cooker. Next, 800 mL of 10 mM citrate buffer pH 6.0 was added and the pressure cooker was tightly closed and microwaved for 20 min. The slides were washed under cold running water for 10 min, soaked in 3% H_2_O_2_ in methanol for 10 min, washed under warm running water for 10 min, and then washed with PBS for 3 min.

Excess PBS was removed and a hydrophobic circle was drawn around the sample with a PAP pen (Sigma-Aldrich, St. Louis, MO). Rabbit anti-NMT1 (Proteintech, Rosemont, IL, USA) or rabbit anti-NMT2 (Origene, Rockville, MD, USA) was diluted in Dako antibody diluent buffer (1:50, ~ 400 μg/mL per slide) and incubated in a humidity chamber overnight at 4 °C. Slides were washed in PBS twice for 5 min each, and ~ 4 drops of EnVision + System-HRP labeled polymer (anti-rabbit) (Dako, Agilent Technologies, Santa Clara, CA, USA) were added to each slide and incubated at room temperature for 30 min. The slides were washed twice in PBS for 5 min each, and four drops of liquid diaminobenzidine + substrate chromogen (prepared according to the manufacturer’s instructions; Dako, Agilent Technologies) was added. Slides were developed for 5 min and rinsed with cold running water for 10 min.

The slides were then soaked in 1% CuSO_4_ for 5 min, rinsed briefly with cold running water, counterstained with hematoxylin for 60 s, and rinsed with cold running water. The slides were then dipped in lithium carbonate thrice, rinsed, and dehydrated in a graded series of ethanol. Coverslips were added and the slides were examined with a Nikon Eclipse 80i microscope and photographed with a QImaging camera.

### Bisulfite sequencing

Chromatin DNA was isolated from the cells using the QIAamp DNA and Blood Mini kit from Qiagen. DNA (100 ng to 1 μg/20 μL) was converted using an EpiTech Bisulfite kit (Qiagen) and amplified using bisulfite-specific primers [28]. The amplified PCR products were purified using the QIAquick PCR Purification kit (Qiagen) and cloned using the TA Cloning Kit (Life Technologies) and the pC2.1 Vector. Sequences were analyzed using QUMA.

### Droplet digital PCR of B-cell lymphoma cell lines

Droplet digital PCR (ddPCR) allows absolute quantification of target DNA copies without the need for running standards. It is based on sample partitioning into approximately 15,000–20,000 droplets (QX200 Droplet Generator, Bio-Rad) that are PCR amplified and fluorescently labeled, read in an automated droplet flow cytometer (QX200 Droplet Reader, Bio-Rad), individually assigned as positive or negative values based on fluorescent intensity, and ultimately quantified with Bio-Rad QuantaSoft software (copies/μl). Each ddPCR mixture (20 μL) was composed of 10 μL of 2X ddPCR Supermix for probes (no dUTP) (Bio-Rad), 250 nM of the same NMT1 and NMT2 TaqMan probes, and 1 μl of the DNA template. The generated droplets were amplified by PCR in a 96-well plate sealed with sealing foil using a C1000 Touch Thermal Cycler (Bio-Rad) (PCR conditions: enzyme activation for 10 min at 95 °C, 40 cycles of denaturation for 30 s at 94 °C, and extension for 1 min at 55 °C with a ramp rate of 2 °C/s, enzyme deactivation for 10 min at 98 °C, and a hold at 4 °C).

### Cell treatments with inhibitors of the proteasome, histone acetylation and DNA methylation

IM9, KMH2, BL2, and Ramos cells were plated (3 × 10^6^ cells per well in a 6-well plate) and treated with the 10 mM proteasome inhibitor MG-132 (Enzo Life Sciences, Farmingdale, NY) for 5 h. Equal amounts of dimethyl sulfoxide were added to the control samples. Cells were lysed and subjected to SDS-PAGE. Western blotting was performed using antibodies against NMT1, NMT2, MCL-1, and GAPDH as the loading controls.

IM9 and BL2 cells were plated at 3 × 10^6^ cells per well in a six-well dish and treated with 1 mM histone acetylation inhibitor suberoylanilide hydroxamic acid (SAHA) and/or increasing concentrations of the DNA methylation inhibitor 5-aza-2’-deoxycytidine (DAC) for 24 h. Equal amounts of dimethyl sulfoxide were added to the control samples. Cells were lysed and subjected to SDS-PAGE. Western blotting was performed using antibodies against NMT1, NMT2, and GAPDH as the loading controls.

### Cell viability measured by FACS

IM9 and BL2 cells were washed with PBS and resuspended at a density of 2.5 × 10^6^ cells per 100 µL of Buffer R prior to electroporation using the Neon Transfection System (Life Technologies). Five micrograms of pBI-CMV2-GFP/NMT2/plasmid DNA were mixed with 100 µL of each cell. IM9 and BL2 cells were electroporated with one pulse of 1700 V for 20 ms and two pulses of 1400 V for 20 ms. Transfected cells were cultured in RPMI 1640 medium supplemented with 10% FBS. At 48 h post-transfection, cell viability was measured by staining the cells with Ghost Violet 540 viability dye (Tonbo Biosciences) for 20 min at room temperature and then fixing them with IntraPrep fixation reagent (Beckman Coulter) according to the manufacturer’s instructions. Samples were analyzed using a BD LSRFortessa Cell Analyzer (BD Biosciences) to assess the viability of GFP-positive cell populations, and the data were analyzed using FCS Express 5 (De Novo Software, Glendale, CA, USA). The experiment was repeated three times.

### Generation of NMT2 and NDUFAF4 knock-out cell lines

Guide RNAs targeting human *NMT2* were prepared using Alt-R CRISPR-Cas9 tracrRNA, ATTO-550 (IDT), and Alt-R CRISPR-Cas9 crRNA (IDT) as previously described [95]. EnGen Cas9 NLS and S. pyogenes (NEB) proteins were incubated with prepared gRNAs to form ribonucleoprotein complexes, which were subsequently transfected into HAP1 cells using Lipofectamine CRISPRMAX according to the manufacturer’s instructions. The next day, cells positive for ATTO-550 were sorted individually into the wells of a 96-well plate on a FACSAria-III (Flow Cytometry Core; University of Alberta) to establish monoclonal populations. Cells were validated for NMT1 or NMT2 protein knockouts by western blotting.

This protocol was followed to generate a constitutive HAP1-*NDUFAF4*-knockout cell lines. Three gRNAs targeting the genomic region of human *NDUFAF4* exon 1 were prepared by using Alt-R CRISPR-Cas9 crRNA complexed with Alt-R CRISPR Cas9 tracrRNA-ATTO 550, as previously described. The EnGen Spy Cas9 was incubated with prepared gRNAs to form the RNP complexes. HAP1 cells were subsequently transfected with these complexes and Lipofectamine CRISPRMAX according to the manufacturer’s instructions. The next day, cells positive for ATTO-550 were single-cell sorted into the wells of a 96-well plate on a FACSAria-III (Flow Cytometry Core; University of Alberta) to establish monoclonal populations. Cells were validated for NDUFAF4 protein knockouts by western blotting.

### Generation of inducible knock-out cell lines

Inducible NMT1 knockout HAP1 cells were prepared as previously described [34]. Briefly, an NMT1-targeting Cas9 spacer sequence was cloned into the Bsmb1 site of FgH1tUTG (Addgene #70183) to generate an inducible gRNA-expression vector. Lentiviral particles were produced through calcium phosphate transfection of 293T cells using 12.5 µg of either FgH1tUTG-NMT1 or FUCas9Cherry (Addgene #70182) along with the packaging plasmid pMD2.G (3.125 µg), and psPAX2 (9.375 µg). 48 h after transfection, the virus-containing supernatant was harvested, centrifuged at 2,000 × g to remove cellular debris, and passed through a 0.45 µm filter. For infection of HAP1 cells, cells were plated at 0.36 × 10^6^ cells per well in a 6-well plate the day before infection. On the day of infection, the medium was replaced with 1 mL of virus-containing medium (FgH1tUTG-NMT1 and FUCas9Chery), and the cells were returned to the incubator. After 72 h of infection, cells that were dual-positive for both GFP and mCherry were sorted singly into the wells of a 96-well plate on a FACSAria-III (Flow Cytometry Core; University of Alberta) to establish monoclonal populations. Following colony expansion, the cells were validated for inducible Cas9-mediated cleavage of the NMT1 target site by addition of doxycycline at a final concentration of 1 µg/mL. Induction was allowed to proceed for 48 h, followed by gDNA isolation, PCR amplification, and T7 endonuclease I digestion to measure Cas9-mediated indels [96]. Clones displaying high levels of Cas9 cleavage at the *NMT1* target site were selected for downstream application.

### Viability of cells treated with PCLX-001

IM9, BL2, and HAP1 cells (1 × 10^5^ cells) were grown in six-well plates in 4 mL media/well and incubated with increasing concentrations of PCLX-001 (0–5 μM) for 96 h. The viability of cells treated with PCLX-001 was measured using the CellTiter-Blue Cell Viability Assay (Promega), and cells were counted with a Cytation 5 cell imaging multi-mode reader (BioTek Instruments) and Gen5 Data Analysis software.

### Sample preparation for mass spectrometry

Cell lysates (30 μg protein/lane, *n* = 3) were separated by sodium dodecyl sulfate–polyacrylamide gel electrophoresis for in-gel digestion. The gels were fixed for 20 min (50% ethanol and 2% phosphoric acid), washed twice for 20 min each (ddH2O), stained overnight with blue-silver Coomassie stain (20% ethanol, 10% phosphoric acid, 750 mM ammonium sulfate, and 0.12% Coomassie Blue G-250), and washed twice for 10 min each (ddH2O). Each lane was separated into 11 fractions and cut into 1 mm pieces. The gel bands were transferred to a round-bottom 96-well plate and destained (50 mM ammonium bicarbonate and 50% acetonitrile) at 37 °C for 10 min. The solution was removed from the wells, and destaining was repeated three times. The gel bands were then dehydrated in acetonitrile at 37 °C for 10 min. Dehydration was repeated twice until the gel bands became white (2 times). The solution was removed and the samples were dried at 37 °C for 10 min. The proteins were reduced (100 mM ammonium bicarbonate, 11.4 mM β-mercaptoethanol) at 37 °C for 30 min then alkylated (100 mM ammonium bicarbonate, 10 mg/mL iodoacetamide) at 37 °C for 30 min. The gel bands were washed twice with 100 mM ammonium bicarbonate at 37 °C for 10 min, dehydrated twice in acetonitrile at 37 °C for 10 min, and dried at 37 °C for 15 min. Proteins were trypsinized (90 μL of 100 mM ammonium bicarbonate and 6 μg/mL sequence-grade modified trypsin; Promega Inc.) overnight. The solutions containing tryptic peptides were transferred to a round-bottom 96-well plate. Tryptic peptides were extracted from the gel bands using an extraction solution (2% acetonitrile and 1% formic acid) and incubated at 37 °C for 1 h. Final extraction was conducted using 50% acetonitrile and 0.5% formic acid and incubated at 37 °C for 1 h. All solutions containing tryptic peptides were transferred to round-bottom 96-well plates and lyophilized overnight. The samples were resuspended (5 fractions per lane) in 0.1% formic acid for liquid chromatography-tandem mass spectrometry (LC–MS/MS) analysis.

### Mass spectrometry analysis

The samples were analyzed using nanoflow-HPLC (Thermo Scientific EASY-nLC 1200 System) coupled to an Orbitrap Fusion Lumos Tribrid Mass Spectrometer (Thermo Fisher Scientific). The peptide mixture underwent reverse phase separation using a trap column (5 µm, 100 Å, 100 µm × 2 cm, Acclaim PepMap 100 nanoViper C18; Thermo Fisher Scientific) and an analytical column (2 µm, 100 Å, 50 µm × 15 cm, PepMap RSLC C18; Thermo Fisher Scientific) for the NMT knockouts and controls, or an analytical column (Aurora Ultimate nanoflow UHPLC column 25 cm × 75 µm ID, 1.7 µm C18, 120 Å; IonOpticks Inc.) for the PCLX-001 treated samples and controls. Peptides were eluted over a 60 min linear gradient from 0 to 36.8% acetonitrile in 0.1% formic acid. Data analysis was conducted using ProteomeDiscoverer (v2.4.1.15) software with the Homo sapiens proteome (Proteome ID UP000005640, download date:2020/02/18). The search parameters included a maximum of three missed trypsin cleavages, a precursor mass tolerance of 15 ppm, a fragment mass tolerance of 0.8 Da, carbamidomethylation of Cys (static modification), and oxidation of Met and deamidation of Asn and Gln (dynamic modifications). A decoy database search was conducted to evaluate the false-positive rates. The strict target false discovery rate was set at 0.01 and the relaxed false discovery rate was set at 0.05. Additional filters included proteins identified at medium and high confidence, with two or more peptides in two or more biological replicates, and master proteins (the top-ranking protein of a group). Proteins with a p-value less than 0.05 and a fold change threshold of 2 compared to the wildtype control were identified as increased or decreased. The MS data were available through MassIVE (MSV ID: MSV000091913).

### Library construction, quality control and RNA sequencing

Messenger RNA was purified from the total RNA using poly T oligo-attached magnetic beads. After fragmentation, first-strand cDNA was synthesized using random hexamer primers, followed by second-strand cDNA synthesis using either dUTP for the directional library or dTTP for the non-directional library. The non-directional library was prepared after end repair, A-tailing, adapter ligation, size selection, amplification, and purification.

A directional library was prepared after end repair, A-tailing, adapter ligation, size selection, USER enzyme digestion, amplification, and purification.

The library was checked with Qubit and real-time PCR for quantification and a bioanalyzer for size distribution detection. Quantified libraries were pooled and sequenced on Illumina platforms according to the effective library concentration and data amount.

### Clustering and RNA sequencing

Clustering of index-coded samples was performed according to the manufacturer’s instructions. After cluster generation, the library preparations were sequenced on an Illumina platform and paired-end reads were generated.

### RNAseq quality control

Raw data (raw reads) in fastq format were first processed using in-house Perl scripts. In this step, clean data (clean reads) were obtained by removing reads containing adapters, poly-N, and low-quality reads from raw data. At the same time, the Q20, Q30, and GC contents of the clean data were calculated. All downstream analyses were based on high-quality clean data.

### Reads mapping to the reference genome

The reference genome and gene model annotation files were downloaded directly from the genome website. The index of the reference genome was built using Hisat2 v2.0.5, and clean paired-end reads were aligned to the reference genome using Hisat2 v2.0.5. We selected Hisat2 as the mapping tool because Hisat2 can generate a database of splice junctions based on the gene model annotation file and thus provide a better mapping result than other non-splice mapping tools.

### Quantification of gene expression level

featureCounts v1.5.0-p3 was used to count the number of reads mapped to each gene. Then, the FPKM of each gene was calculated based on the length of the gene and the read count mapped to this gene. FPKM, the expected number of Fragments Per Kilobase of transcript sequence per million base pairs sequenced, considers the effect of sequencing depth and gene length for the read count at the same time and is currently the most used method for estimating gene expression levels.

### Metabolomics

#### Sample preparation—metabolite extraction

One hundred microliters of 1:1 (v/v) LC–MS-grade methanol:water was added to each individual sample tube. The samples were then immersed in liquid nitrogen for 30 s and thawed in water. The freeze–thaw cycle was repeated five times for each tube. The tubes were then centrifuged at 12,000 rpm for 10 min at 4 °C. The supernatant was then transferred to a new vial and dried. The samples were dissolved in 100 μL water.

### Sample normalization and aliquoting

The total sample concentrations were determined using a NovaMT Sample Normalization Kit. The samples were split into six aliquots for different labeling methods, backups, and pooled sample preparation. Each aliquot contained 10 μL of the cell extract. An aliquot for preparing pooled samples from each individual sample was combined and mixed thoroughly to generate a pooled sample that was used as a reference.

### Chemical isotope labeling

For each aliquot of the sample for amine-/phenol-labeling, LC–MS-grade water was added to dilute the sample. The labeling protocol strictly followed the SOP provided in the kit. Briefly, 5 μL of Reagent A (Reagent A) and 15 μL of 12C2-labeling (for the individual and pooled samples) or 13C2-labeling (for the pooled sample) reagent (Reagent B) were added to the samples. The samples were vortexed and spun down. The mixtures were incubated at 40 °C for 45 min. Subsequently, 3 μL of quenching reagent (Reagent C) was added to quench excess labeling reagent. The mixtures were incubated at 40 °C for 10 min. Finally, 12 μL of pH-adjusting reagent (Reagent D) was added.

For one aliquot of the sample for carboxyl labeling, the labeling protocol strictly followed the SOP provided in the kit. Briefly, 4 μL of catalyzing Reagent A (Reagent A) and 10 μL of 12C2-labeling (for the individual samples and the pooled sample) or 13C2-labeling (for the pooled sample) reagent (Reagent B) were added to the samples. The samples were vortexed and spun down. The mixtures were incubated at 80 °C for 60 min. Subsequently, 16 μL of quenching reagent (Reagent C) was added to quench excess labeling reagent. The mixtures were incubated at 80 °C for 30 min, and the chemical isotope-labeling procedure was complete.

### Mixing

The 12C2-labeled individual sample was mixed with 13C2-labeled reference sample in equal volume. The mixture was then subjected to LC–MS analysis. Prior to LC–MS analysis of the entire sample set, a quality control (QC) sample was prepared using an equal-volume mix of pooled 12C-labeled and 13C-labeled samples.

### LC–MS analysis condition

LC–MS analysis strictly followed SOP (i.e., Rapid LC–MS Analysis for HP-CIL Metabolomics Platform). QC samples were injected every six sample runs to monitor the instrument performance.

**Table:** 

Instrument:	Agilent 1290 LC linked to Agilent 6546 Mass Spectrometer
Column:	Agilent eclipse plus reversed phase Cl8 column (150 × 2.1 mm, 1.8 µm particle size)
MPA:	0.1% (v/v) formic acid in water
MPB:	0.1% (v/v) formic acid in acetonitrile
Gradient:	*t* = 0 min, 25% B; *t* = 10 min, 99% B; *t* = 15 min, 99% B; *t* = 15.1 min, 25% B; *t* = l8 min, 25% B
Flow rate:	400 µL/min
Column oven temperature:	40 °C
Mass range:	m/z 220–1000
Acquisition rate:	L Hz

### Data processing

A total of 30 LC–MS data from the 2-channel analysis (15 LC–MS data points, including three QC points in each channel) were exported. csv file using the Agilent MassHunter software. The exported data were uploaded to the IsoMS Pro 1.2.16. Data processing was performed after a quality check. The parameters used for data processing are as follows:

**Table:** 

Minimum m/z:	220
Maximum m/z:	1000
Saturation intensity:	20,000,000
Retention time tolerance	9 s
Mass tolerance:	l0 ppm

### Data cleansing

Ten groups were assigned to 15 LC–MS data in each channel:3 data files labeled as ‘Day3’ group, 3 data files labeled as ‘Day3_Drug’ group, 3 data files labeled as ‘Day5’ group, 3 data files labeled as ‘Day5_Drug’ group, and 3 data files labeled as ‘QC’ group. Peak pairs without data present in at least 80.0% of the samples in any group were filtered out (see Sect. 3.2). The data were normalized to the Ratio of Total Useful Signal.

### Metabolite identification

**Table:** 

Retention time tolerance for CIL library ID	10 s
Retention time tolerance for LI library ID	75 s
Mass tolerance for CIL library ID	l0 ppm
Mass tolerance for LI library ID	l0 ppm
Mass tolerance for mass-based database ID	l0 ppm

### Metabolomics analysis

MetaboAnalyst 5.0 (https://www.metaboanalyst.ca) is a comprehensive platform dedicated to metabolomic data analysis via a user-friendly, web-based interface. The platform was used to perform statistical analysis (one factor) of volcano plots, pathway analysis, and enrichment analysis (MSEA) [97].

### Mitochondrial isolation

Mitochondria were isolated from the cells as previously described [98]. The medium was aspirated, and the cells were scraped in PBS, pelleted, and washed a second time in PBS. Following pelleting, the cells were resuspended in 1.05 ml of mitochondrial isolation buffer (MIB), and 50 µL was removed and stored at −80 °C for whole cell analysis. Mitochondria were isolated by drawing into and expelling the cell solution from a 3 ml syringe using 18.5 gauge and 27 gauge needles respectively, for a total of six times. Cell debris was pelleted twice: first in a 50 ml conical tube, followed by transfer of the supernatant, and a second centrifugation step in a 15 ml conical tube. The MIB enriched in mitochondria was transferred to a 1.5 ml microfuge tube, and mitochondria were pelleted at 10 k × g for 5 min, washed in 1 ml MIB, pelleted again as above, and finally resuspended in 100 µL MIB. Isolated mitochondria were stored at −80 °C until further processing.

### Mitochondrial membrane solubilization

Mitochondrial membranes were solubilized as per [99] at 5 mg/ml, and the final supernatant was stored at −80 °C until blue native PAGE was performed.

### Blue-native PAGE of mitochondrial OXPHOS complexes

Blue native PAGE was performed on solubilized mitochondria using the buffers and methods described in [99], with the following modifications. PAGE was performed with 10–20 ug protein/lane for western blotting and 20–40 ug/lane for in-gel complex I activity assays using 8% resolving and 4% stacking wide-pore gels prepared from a stock acrylamide solution containing 50% (w/v) acrylamide and 0.5% (w/v) bis acrylamide (100:1 acrylamide: bisacrylamide) [100]. Electrophoresis was performed at 10 mA/gel until the voltage reached 250 V, at which point the current was reduced to 5 mA/gel. Electrophoresis was performed for 40 min after the front left side of the gel. Cathode buffer A was replaced with cathode buffer B when the front reached the halfway point of the gel.

### Transfer and western blotting

Following electrophoresis, the gels were transferred to PVDF membranes following the protocol described in [99], with the transfer time at 300 mA increased to 4 h to obtain the best results. Membranes were blocked 1 h in 5% BSA PBS 0.1% Tween-20 (blocking buffer) and incubated overnight in blocking buffer containing either Rb α-NDUFB11 (ab183716 Abcam) to detect intact complex I or total OXPHOS antibody cocktail (ab110413 Abcam) to detect levels of the remaining OXPHOS complexes. The secondary goat α-Rb and goat α-ms HRP-conjugated antibodies were incubated in blocking buffer for 1 h at room temperature. After washing, the membranes were developed using SuperSignal™ West Pico PLUS Chemiluminescent Substrate (Thermo Scientific 34,577) and visualized using ImageQuant 800 (Amersham).

### In-gel complex I activity assays

In-gel complex I activity assays were performed following blue native PAGE, as previously described [99], for 1 h. The gels were visualized and imaged using ImageQuant 800 (Amersham, UK) before and after the activity assay. To accurately quantify the blue/purple precipitate, the blue background (Coomassie G-250) was removed by soaking the gels in 5% methanol for 2 h (to shrink the swollen gels), followed by overnight incubation in a de-staining solution containing 10% methanol and 10% acetic acid.

### Complex I enzymatic assay

HAP 1 cells were treated with 1 µg/mL doxycycline or not and incubated for 96 h. Cells were scraped, centrifuged, and resuspended in PBS. Complex I activity was determined using the Complex I Enzyme Activity Microplate Assay Kit (Abcam #ab109721). Briefly, protein concentration was determined using the Pierce BCA Protein Assay Kit. The cell concentration of the initial solution was calculated, and the extracts were prepared using a detergent kit followed by centrifugation. A final sample concentration of 750 µg/mL was used to incubate the extracts in the plate. After 3 h, each well was washed thrice with wash buffer. After the addition of the assay buffer, the absorbance of each well was measured for 60 min at 450 nm using a Cytation 5 cell imaging multi-mode reader (BioTek Instruments) and Gen5 Data Analysis software.

### Respirometry measurements

HAP1 cell cultured in IMDM supplemented with 10% FBS and 1% antibiotics were trypsinized and counted using the Countess 3 automated cell counter (Thermo Fisher Scientific). Cells were then seeded at 5000–7500 cells/well in media containing 1 μg/mL doxycycline or not. Cells were allowed to adhere to the plate for 1 h at 37 °C with 5% CO2 before changing the lid to an Oxygen Consumption Rate (OCR) sensing lid and attaching the Resipher cell culture monitor (Lucid Scientific) to the lid after placing the plate back into the incubator. OCR was measured for 24 h before refreshing the media with normal media, media containing doxycycline, or media containing 0.5 μM PCLX-001 or 1 μM IACS-10759 [101]. OCR was measured approximately 120 h after treatment. OCR data were downloaded from the Lucid Lab online portal, processed using Microsoft Excel, and subsequently graphed and analyzed using Prism software.

### Gene set enrichment analysis and MISS-54 gene set design

Gene Set Enrichment Analysis was performed using GSEA_4.2.3 software [102]. To create a Myristoylation Inhibition Sensitivity Signature, we determined the IC50 (molar) for four cell line screens performed using PCLX-001 [22] and PCLX-002 (ICL1100013 [31]). The expression levels of genes were measured in transcripts per million (TPM), and a value known as pIC50 was calculated using the formula −log10(IC50[Molar]). This value was selected as the phenotype vector for correlation analysis. The pIC50 value for each sample was exported to GSEA as a "Continuous vector" CLS file. The Pearson correlation coefficient between the TPMs of genes and pIC50 was used as the gene-ranking metric. Gene sets consisting of more than 10 members and less than 1000 members were considered for analysis. The default value of 1000 permutations was selected for the phenotype permutation test to determine the significance of the gene sets. Leading Edge Analysis was performed and genes associated with myristoylation inhibition sensitivity were determined for each screen. MISS-54 represented the 54 genes independently associated with myristoylation inhibition sensitivity in all four screens (Additional file 2: Table S3). MISS-54 score was defined as the mean value of log_2_(TPM + 1) for each gene [41].

### Over-representation analysis (ORA)

The 54 genes present in MISS-54 were used for ORA analysis using the online platform WebGestalt [103] (https://www.webgestalt.org). Homo Sapiens was used as the target organism. Genes were compared to Gene Ontology Biological Process noRedundant, geneontology Molecular Function noRedundant, geneontology Cellular Component noRedundant, and disease GLAD4U [104]. Genome protein coding was used as the Reference Set.

### Statistical methods

Data were analyzed using Prism 9.1.0 software (GraphPad) and generally expressed as mean ± s.e.m. unless stated otherwise.

### Supplementary Information

**Additional file 1: Figure S1.** *NMT2* expression level is lower in hematologic cancer cells and tumors*. NMT2* mRNA expression was significantly lower in hematologic cancer cells (**A**) and tumors (**B**) than in cells and tumors of other origins (min-to-max box plot; ***p < 0.0001). Data were extracted from Depmap 22Q4 and TCGA databases. **Figure S2.** NMT1 expression is stable in multiple hematologic cancer cell lines and tumors. NMT1 levels were assessed by western blotting on 35 μg of cell lysate proteins in immortalized “normal” human B-cell line IM9, neoplastic B-cell lymphoma cell lines, leukemic T-cell lines, and lysates of various types of human solid lymphomas. **Figure S3.** *NMT1* and *NMT2* expression in tumors and associated normal tissues. Graphs were obtained using the GEPIA2 platform [41] for cancer selection. Although *NMT2* expression was lower in all tumors than that in the associated normal tissues (other than DLBC, PAAD, and LIHC), *NMT1* expression was higher in all tumors than that in the associated normal tissues (other than LAML, BRCA, and PRAD). **Figure S4.** *NMT2* expression is significantly decreased in metastases. Data were collected using the TNMplot platform [107]. Box plots demonstrate that while *NMT1* expression is increased in numerous tumors and metastases (**A**), *NMT2* expression is significantly lower in metastases than in the associated normal tissue in Breast, Colon, Lung and Ovarian cancers (**B**). **Figure S5.** *NMT1* and *NMT2* mRNA and protein levels are correlated in cancer cell lines. *NMT1* and *NMT2* RNA expression (**A**) and protein levels (**B**) were extracted from the Depmap (22Q2), revealing a significant positive correlation between *NMT1* and *NMT2*. **Figure S6.** Hematologic cancer cell lines are more dependent on *NMT1.* Dependency scores (median non-essential KO effect is 0 and median essential KO effect is −1) obtained after *NMT* CRISPR knockout in 115 hematologic cell lines (**B**). *NMT1* dependency score box plot in hematologic cancer cells compared with cancers of other origins. **Figure S7.** Hematologic and bladder cancers are highly dependent on *NMT1. NMT1* Dependency Scores were extracted from the Depmap platform (22Q4) and sorted according to cancer of origin. Lymphoma, Bladder and Myeloid cell lines were the most sensitive to *NMT1* KO, whereas thyroid, pleural, and kidney cell lines were moderately affected by *NMT1* KO. (P. N. S. is the peripheral nervous system). **Figure S8.** *NMT1* and *NMT2* survival maps. Low *NMT2* expression is associated with poor prognosis in kidney cancer, glioma, ovarian cancer, and leukemia. Heat maps represent hazard ratios calculated for *NMT1* and *NMT2* in multiple cancer types for disease-free survival (DFS) and Overall Survival (OS). The group cut-off was set as the median value. The significance level was set at *p* < 0.05. Red and blue blocks represent higher and lower risks, respectively, with an increase in gene expression. Blocks with red or blue frames indicate statistical significance. Heat maps were obtained using the GEPIA2 platform [41]. **Figure S9.** *NMT1* and *NMT2* gene expression and DNA methylation in multiple cancers. Aggregation methylation beta values at the *NMT1* locus in tumors and normal tissues showed increased methylation in breast, colorectal, head and neck compared to those in associated normal tissues whereas it was decreased in bladder, kidney, liver, lung, pancreas, and thyroid (**A**). This analysis revealed 12 methylation sites at the *NMT2* locus. Site cg02268561 is the site that participates in the downregulation of *NMT2* levels in lymphoma, leukemia, breast cancer, and lung cancer. Data collected from the SMART ((Shiny Methylation Analysis Resource Tool) platform [108]. Detailed correlation between methylation beta values and *NMT2* expression for 12 methylation sites in DLBCL (**B**), LAML (**C**), lung (**D**), and breast (**E**). **Figure S10.** Validation of bisulfite sequencing methodology. Bisulfite sequencing of the *DAPK1* promoter region revealed a highly methylated CpG containing promoter region only in malignant B lymphocytes, as previously described [29]. **Figure S11.** Effects of acute DAC and SAHA treatment on *NMT1* and *NMT2* levels in normal and malignant lymphocytic cell lines. Number of copies of *NMT1* mRNA in BL2 (**A**), IM9 (**B**), DOHH2 (**D**), and WSU-DLCL2 (**F**) after 24 h of treatment with increasing concentrations of demethylation agent (DAC) in the presence of histone deacetylase inhibitor (SAHA). Number of copies of *NMT2* mRNA in the DOHH2 (**C**) and WSU-DLCL2 (**E**) cell lines after 24 h of treatment with increasing concentrations of demethylation agent (DAC) in the presence of histone deacetylase inhibitor (SAHA). Overall, DAC and SAHA treatment reduced the expression of *NMT1* in a time- and concentration-dependent manner in all cell lines. Values represent mean ± s.e.m. of three independent experiments. Samples sharing letters are not statistically different (one-way ANOVA). **Figure S12.** Correlation between *NMT* expression and EC_50_/IC_50_ in four cell line screens treated with PCLX-001 or PCLX-002. Aggregates of over 1200 cell lines were treated with PCLX-001 [22] or PCLX-002 (or ICL1100013 [31]) for 96 h or 72 h respectively and viability was measured using Cell Titer Blue® and Syto60, respectively. The sensitivity to PCLX compounds was poorly correlated with both *NMT1* and *NMT2* levels. A weak but significant correlation was found between *NMT1* and *NMT2* levels and IC_50_ for PCLX-002 (*r* = 0.043 and *r* = 0.1031, respectively). Red dots represent hematologic cancer cell lines and correlation. No significant correlation was found with NMT1/NMT2 protein levels (data not shown). **Figure S13.** *NMT1* and *NMT2* are not the genes that correlate the most with sensitivity to PCLX-002. Volcano plot describing the Pearson correlation between gene expression and IC_50_ for PCLX-002 in cancer cell lines. Data analysis was performed using the DepMap portal. *NMT1* and *NMT2* are shown in red, and myristoylated proteins are shown in green. **Figure S14.** Establishment of a Myristoylation Inhibition Sensitivity Signature gene set. Cells were selected from four cell line screens (three screens for PCLX-001 [22] and one screen for PCLX-002 [31]). Four GSEA were performed with −log(IC_50_) used as a continuous phenotype vector to identify hallmark pathways that were enriched in the sensitive population. We performed four leading-edge analyses on the positively enriched pathways for each screen and identified 54 genes that were found in all screens. The obtained gene set was named myristoylation inhibition sensitivity signature 54 (MISS-54) (**A**). Hallmark pathways enriched in sensitive cancer cell lines sorted by the average normalized enrichment score (**B**). **Figure S15.** Maps of MISS-54 scores for clustered tumors, normal tissues, and cancer cell lines. RNA expression was used to cluster 11,070 tumors (TCGA) (**A, B**), 17,382 normal tissue samples (GTEx) (**C**) and 1408 cancer cell lines (CCLE) (**D**). MISS-54 can be visualized by the blue or pink color scale, revealing lymphoma, leukemia, and other solid tumor cancers that are predicted to be sensitive to PCLX-001 and PCLX-002 treatment. The MISS-54 score is significantly lower in paracancerous normal tissue than in associated tumors (expression profile from TCGA) in multiple cancers (with the exception of KIHC and LUSC) (**B**). Clustering maps were designed using tumormap (https://tumormap.ucsc.edu) and showed the distribution of the different cancers predicted to be sensitive to PCLX-001 treatment with a high MISS-54 score. **Figure S16.** MISS-54 scores in cancer patients sorted by sex and clinicopathological parameters. TCGA tumor MISS-54 scores were sorted according to sex (**A**), age (**B**), smoking (**C**), histological grade (**D**), clinical grade (**E**), and pathological grade (**F**). A t-test was performed for significant differences between the groups (*p* < 0.05). **Figure S17.** MISS-54 survival map. Genes included in MISS-54 are associated with poorer prognosis in the patients with leukemia, liver, gliomas, adrenal gland cancers. Heat maps represent hazard ratios calculated for 54 genes in multiple types of cancers for overall survival (**A**) and disease-free survival (**B**). The group cut-off was set as the median value. The significance level was set at *p* < 0.05. Red and blue blocks represent higher and lower risks, respectively, with an increase in gene expression. Blocks with red or blue frames indicate statistical significance. Heat maps were generated using the GEPIA2 platform [41]. The majority of the 54 genes present in MISS-54 were positively associated with low survival (red squares), indicating that patients with high MISS-54 (sensitive to PCLX-001) did not respond to the actual standard of care and had low survival. **Figure S18.** PCLX-001 resistant DOHH2 cells have higher NMT2 protein levels. DOHH2 DLBCL cancer cells were grown for 6 months with increasing concentrations of PCLX-001 (from 2 to 20 nM final) to selectively isolate resistant cells. Viability curves demonstrated a significant increase in the resistance of cells to PCLX-001 treatment for 96 h (*n* = 3) (**A**). The corresponding histogram shows a significant ~ twofold increase in calculated EC_50_ values. (**B**) Selectively isolated PCLX-001 DOHH2 resistant cells show a significant increase in NMT2 protein levels. **Figure S19.** Confirmation of inducible Cas9-mediated cleavage at the *NMT1* target site and time-dependent *NMT1* knockout in HAP1 *NMT1* inducible KO cells. Sanger sequencing trace of the *NMT1* target site following doxycycline treatment in either parental HAP1 cells (above) or cells containing the inducible Cas9 system (below) (**A**). Doxycycline was added to the growth medium at a final concentration of 1 µg/mL for 48 h, followed by gDNA extraction, PCR amplification, and Sanger sequencing of the amplicon. The presence of multiple base calls in the Sanger sequencing trace was indicative of indels generated by Cas9 cleavage. HAP1 cells were treated with doxycycline (1 μg/ml) for up to 72 h. Western blot analysis revealed *NMT1* time-dependent KO in HAP1 inducible *NMT1* KO cells and HAP1 inducible *NMT1* KO/*NMT2* KO cells (**B**). 72 h doxycycline treatment is necessary to produce functional *NMT1* KO cells. **Figure S20.** Doxycycline treatment of HAP1 *NMT1* inducible KO and *NMT2* KO/inducible *NMT1* inducible KO cells safely abrogates the expression of *NMT1* leaving the cells more sensitive to PCLX-001 treatment. Viability of inducible *NMT1* KO HAP1 cells and *NMT2* KO/inducible *NMT1* KO HAP1 cells incubated with or without doxycycline (1 μg/ml) and with increasing concentration of PCLX-001 for 96 h (**A**), or pre-incubated with/without doxycycline (1 μg/ml) for 72 h then with increasing concentration of PCLX-001 for 96 h (**B**). **Figure S21.** The proteins downregulated or upregulated in HAP1 cells with various *NMT* KOs and PCLX-001 treatment do not overlap. Venn Diagram displaying the different groups of proteins downregulated or upregulated with different treatments. Owing to the potency and toxicity of PCLX-001, multiple pathways, such as epithelial mesenchymal transition, unfolded protein response, hypoxia, and apoptosis, are rapidly upregulated, resulting in different populations that do not fully overlap. This could also be explained by the inducible *NMT1* KO system, which could leave some residual NMT1 in the cell. **Figure S22.** GSEA describing the differentially expressed genes in *NMT* KOs or PCLX-001 treated HAP1 cells. RNA sequencing (RNA-seq) analysis (Novogene Inc.;Sacramento, CA, USA) was performed on HAP1 cells lacking *NMT1, NMT2,* or both, or treated with PCLX-001, as previously described. Hallmark GSEA results for HAP1 *NMT1* KO (**A**), *NMT2* KO (**B**), double *NMT1/NMT2* KO (**C**), 0.5 µM PCLX-001 treatment (**D**) and 5 µM PCLX-001 treatment (**E**) for 72 h. **Figure S23.** *NDUFAF4* KO in HAP1 cells results to complex I mis-assembly, degradation, and reduced activity. Western blot analysis of NDUFAF4 (**A**) from the mitochondria of HAP1 parental cells and engineered *NDUFAF4* KO (2 clones). Isolated mitochondrial Complex I (NDUFB11) Blue Native PAGE immunoblotting (**B**) of HAP1 parental cells and engineered *NDUFAF4* KO (2 clones). In gel complex I diaphorase activity assay of isolated mitochondria from HAP1 parental cells and engineered *NDUFAF4* KO (2 clones). Histograms represent quantification (normalized to VDAC expression) of three independent experiments. **Figure S24.** Time-dependent impact of *NMT* KOs on the oxygen consumption rate in HAP1 cells. Oxygen consumption rate (OCR) histograms of HAP1 cells after 0.5 µM PCLX-001 or 1 µM IACS10759 treatment at 24 (**A**), 48 (**B**), 72 (**C**), and 96-h (**D**) time points after drug treatment or not, with time 0 representing the addition of PCLX-001 or IACS010759. Doxycycline was added as a 24-h pre-treatment to HAP1 cells with inducible *NMT1* KO when initially seeding the cells (*n* = 3). **Figure S25.** *NMT1* KO drastically affected cellular metabolite levels in HAP1 cells. A volcano plot was constructed by plotting the fold change (FC) of each metabolite against the *p* value (**A**). The fold change was calculated as Mean (Day3)/Mean (Day3_*NMT1*KO) and Mean(Day5)/Mean(Day5_*NMT1*KO). Metabolites identified in Tiers 1 and 2 as high-confidence results were used for pathway analysis and are represented in a scatter plot (**B**). Pathway analysis was performed using the Global Test for enrichment analysis and Relative-betweeness Centrality as topology analysis in MetaboAnalyst (www.metaboanalyst.ca) for days 3 and 5 of PCLX-001 treatment (**C**). **Figure S26.** *NMT1* KO in HAP1 cells drastically affected the media metabolites. A volcano plot was constructed by plotting the fold change (FC) of each metabolite against the *p* value (**A**). The fold change was calculated as Mean (Day3)/Mean(Day3_*NMT1*KO) and Mean(Day5)/Mean(Day5_*NMT1*KO). Metabolites identified in Tiers 1 and 2 as high-confidence results were used for pathway analysis and are represented in a scatter plot (**B**). Pathway analysis was performed using the Global Test for enrichment analysis and Relative-betweeness Centrality as topology analysis in MetaboAnalyst (www.metaboanalyst.ca) for days 3 and 5 of PCLX-001 treatment (**C**). **Figure S27.** Tumors with low OXPHOS scores are predicted to be more sensitive to myristoylation inhibition. Tumors were scored for glycolysis and oxidative phosphorylation by Bi et al. [36], and the groups were median-separated. While the predicted sensitivities of low- and high-glycolysis-scored tumors were equal (**A**), tumors with High or Low Glycolysis & Low OXPHOS had a significantly higher MISS-54 score (**B**) compared to High OXPHOS scored tumors. **Figure S28.** Tumor clustering based on the expression of MISS-54 genes. RNA expression for the 54 genes included in the MISS-54 gene set was used to cluster 11,070 tumors (TCGA). The clustering map was designed using tumormap (https://tumormap.ucsc.edu). Cancer origin clusters were maintained for DLBC, LAML, LGG, LIHC, and KIRC, but were more diffuse for cancers predicted to be intermediately sensitive. **Figure S29.** MISS-54 Over-Representation Analysis (ORA). Bar charts show the enrichment ratio and significance (FDR) of MISS-54 genes in the most significant gene sets (TOP10) present in the Gene Ontology database for biological processes (**A**), molecular functions (**B**), cellular components (**C**), and disease-GLAD4U (**D**). **Figure S30.** *MYC* expression is negatively correlated with *NMT2 expression* and *NMT1* dependency and positively correlated with MISS-54 score in cancer cell lines. *MYC* and *NMT2* RNA expression (**A**) *NMT1* dependency (**B**) were extracted from Depmap (22Q2), which revealed a significant negative correlation. MISS-54 significantly positively correlates with *MYC* RNA expression (**C**).

**Additional file 2: Table S1.** Univariate and multivariate analyses of high versus low *NMT2* mRNA expression as a prognostic marker for progression-free survival in the GSE31312 cohort. High *NMT2* indicates an *NMT2* mRNA expression level above the median NMT2 level in that cohort, and low *NMT2* indicates an *NMT2* mRNA expression level below the median in that cohort. HR, hazard ratio; CI, confidence interval; GCB, germinal center B‐cell like; ACB, activated B‐cell like; UC, unclassified. **Table S2.** Association of *NMT2* expression with patient clinicopathological features in DBCL (analyzed from the GSE31312 cohort). **Table S3.** PCLX-001 and PCLX-002 sensitive cells have multiple origins. The treated cell lines were grouped according to their sensitivity to PCLX-001 and PCLX-002 (IC50). The cell lines were sorted into quartiles representing NMTI-sensitive (0–25%), less sensitive (25–50%), less resistant (50–75%), and resistant (75–100%) cells. The origin of the cells sensitive to PCLX-001 [22] and PCLX-002 [31] is not restricted to hematologic cancers, but could also be found in cells originating from solid tumors. Therefore, MISS-54 is not a signature of hematological cancers. **Table S4.** List of the 54 genes included in the Myristoylation Inhibition Sensitive Signature (MISS-54). **Table S5.** TCGA study abbreviations. (https://gdc.cancer.gov/resources-tcga-users/tcga-code-tables/tcga-study-abbreviations)

**Additional file 3: **Supplementary data file_MISS54

## Data Availability

Data were extracted from available datasets, including RNA-seq expression data, and dependency scores were obtained from the Depmap portal (https://depmap.org/portal/), tumor RNA expression data from TCGA dataset (https://portal.gdc.cancer.gov), Tumormap (https://tumormap.ucsc.edu), expression and survival data from GEPIA2 platform (http://gepia2.cancer-pku.cn/#index), metastatic RNA expression from TNMplot platform (https://tnmplot.com/analysis/), methylation data from SMART platform (http://www.bioinfo-zs.com/smartapp/), ICL1100013 drug response from GDSC (https://www.cancerrxgene.org), and DLBCL GSE31312 expression data on GEO platform (https://www.ncbi.nlm.nih.gov/geo/). Mass spectrometry proteomics data are available through MassIVE (MSV ID: MSV000091913). The calculated MISS-54 scores for the cell lines, tumors (T), and normal (N) tissues are described in Additional file 3: Supplementary data file_MISS54.

## Abbreviations

NMT: N-myristoyltransferase
NMTI: N-myristoyltransferase inhibitor
MISS: Myristoylation inhibition sensitivity signature
SFK: Src family kinase
CDX: Cell line derived xenograft
PDX: Patient derived xenograft
BCR: B-cell eeceptor
CCLE: Cancer cell line encyclopedia
IHC: Immunohistochemistry
DAC: Deoxyazacytidine
SAHA: Suberoylanilide hydroxamic acid
GSEA: Gene set enrichment analysis
OXPHOS: Oxidative phosphorylation
TCGA: The cancer genome atlas
OCR: Oxygen consumption rate
ETS: Electron transport system
UPR: Unfolded protein response
CSC: Cancer stem cell
ORA: Over-representation analysis
