# Supplementary material for: Multiomics analysis identifies oxidative phosphorylation as a cancer vulnerability arising from myristoylation inhibition
Source: J Transl Med. 2024 May 7;22:431. doi: 10.1186/s12967-024-05150-6 (PMC11075276; doi:10.1186/s12967-024-05150-6)
Supplement: Supplementary file 1 — Additional file 1: Figure S1. NMT2 expression level is lower in hematologic cancer cells and tumors. NMT2 mRNA expression was significantly lower in hematologic cancer cells (A) and tumors (B) than in cells and tumors of other origins (min-to-max box plot; ***p < 0.0001). Data were extracted from Depmap 22Q4 and TCGA databases. Figure S2. NMT1 expression is stable in multiple hematologic cancer cell lines and tumors. NMT1 levels were assessed by western blotting on 35 μg of cell lysate proteins in immortalized “normal” human B-cell line IM9, neoplastic B-cell lymphoma cell lines, leukemic T-cell lines, and lysates of various types of human solid lymphomas. Figure S3. NMT1 and NMT2 expression in tumors and associated normal tissues. Graphs were obtained using the GEPIA2 platform [41] for cancer selection. Although NMT2 expression was lower in all tumors than that in the associated normal tissues (other than DLBC, PAAD, and LIHC), NMT1 expression was higher in all tumors than that in the associated normal tissues (other than LAML, BRCA, and PRAD). Figure S4. NMT2 expression is significantly decreased in metastases. Data were collected using the TNMplot platform [107]. Box plots demonstrate that while NMT1 expression is increased in numerous tumors and metastases (A), NMT2 expression is significantly lower in metastases than in the associated normal tissue in Breast, Colon, Lung and Ovarian cancers (B). Figure S5. NMT1 and NMT2 mRNA and protein levels are correlated in cancer cell lines. NMT1 and NMT2 RNA expression (A) and protein levels (B) were extracted from the Depmap (22Q2), revealing a significant positive correlation between NMT1 and NMT2. Figure S6. Hematologic cancer cell lines are more dependent on NMT1. Dependency scores (median non-essential KO effect is 0 and median essential KO effect is −1) obtained after NMT CRISPR knockout in 115 hematologic cell lines (B). NMT1 dependency score box plot in hematologic cancer cells compared with cancers of other origi [file 12967_2024_5150_MOESM1_ESM.pptx]

## Slide 1
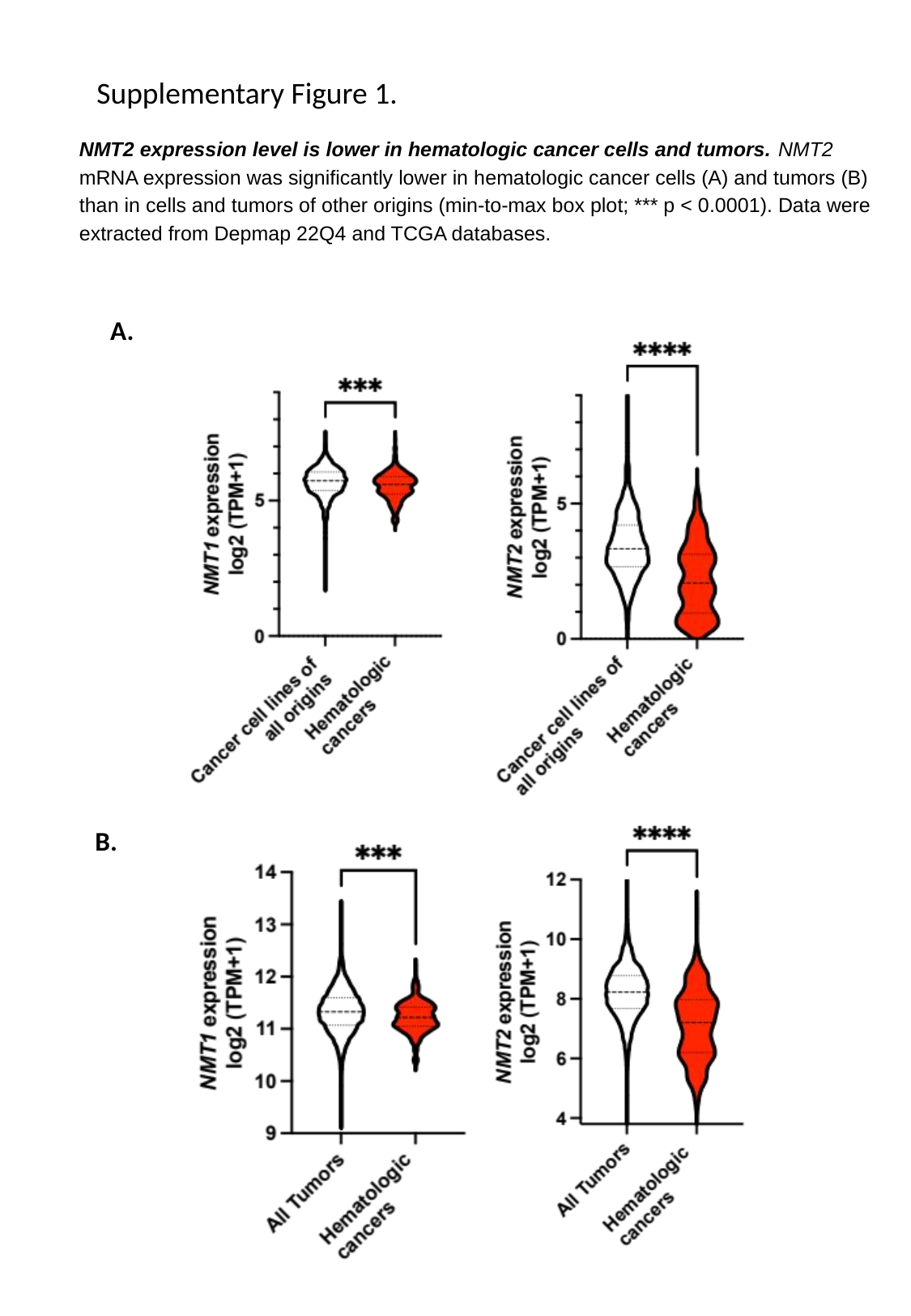

Supplementary Figure 1.
NMT2 expression level is lower in hematologic cancer cells and tumors. NMT2 mRNA expression was significantly lower in hematologic cancer cells (A) and tumors (B) than in cells and tumors of other origins (min-to-max box plot; *** p < 0.0001). Data were extracted from Depmap 22Q4 and TCGA databases.
A.
B.

## Slide 2
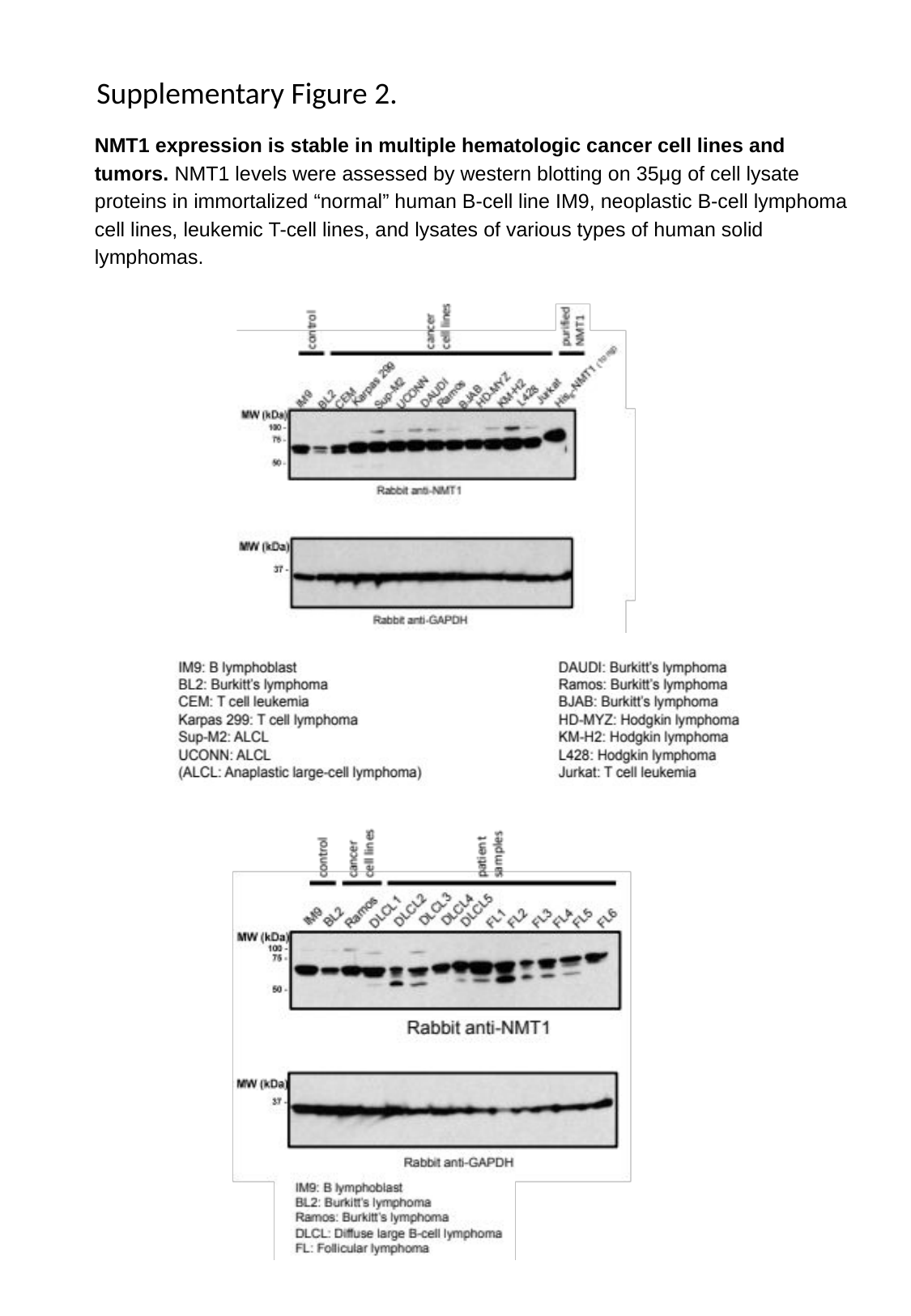

Supplementary Figure 2.
NMT1 expression is stable in multiple hematologic cancer cell lines and tumors. NMT1 levels were assessed by western blotting on 35μg of cell lysate proteins in immortalized “normal” human B-cell line IM9, neoplastic B-cell lymphoma cell lines, leukemic T-cell lines, and lysates of various types of human solid lymphomas.

## Slide 3
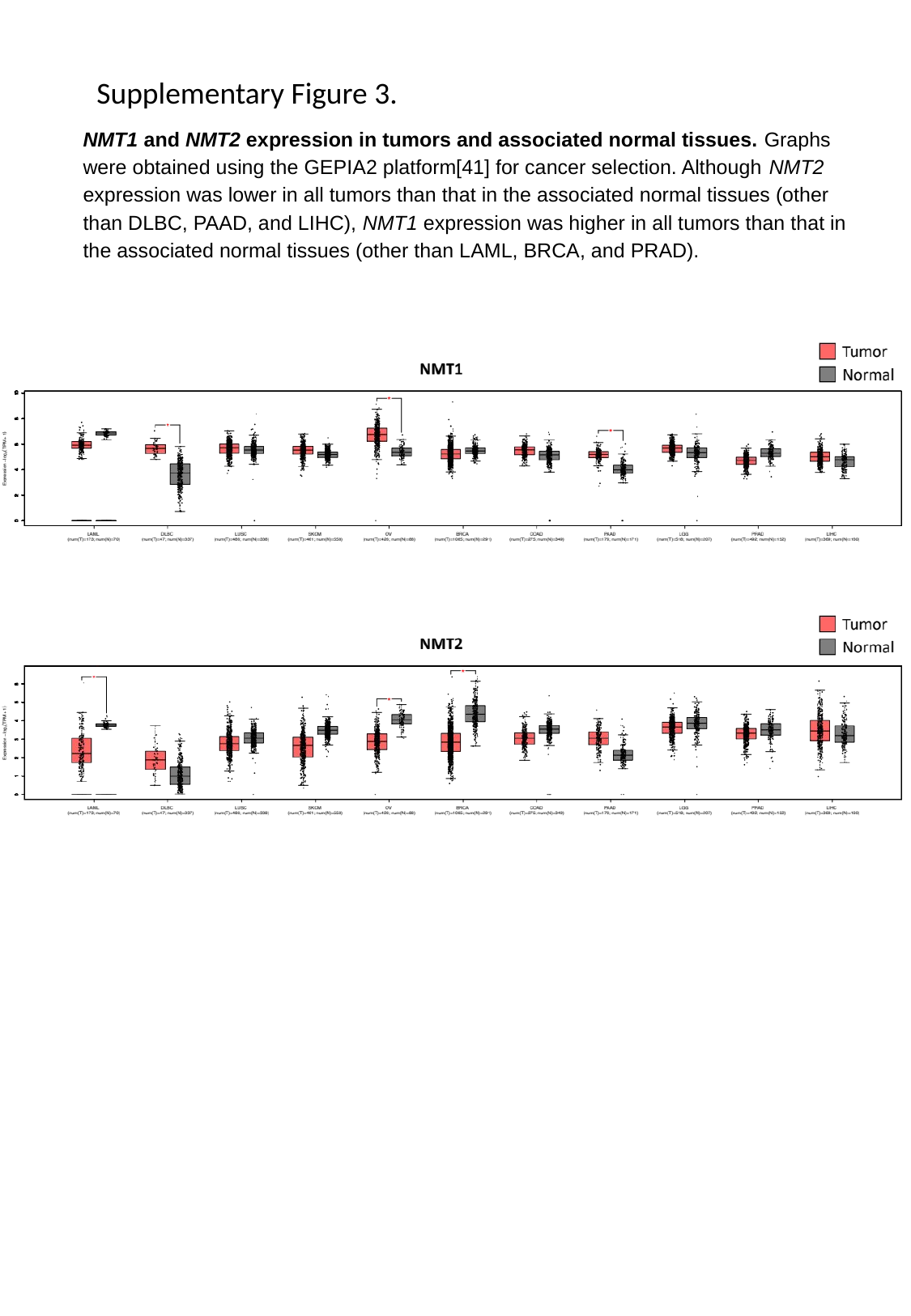

Supplementary Figure 3.
NMT1 and NMT2 expression in tumors and associated normal tissues. Graphs were obtained using the GEPIA2 platform[41] for cancer selection. Although NMT2 expression was lower in all tumors than that in the associated normal tissues (other than DLBC, PAAD, and LIHC), NMT1 expression was higher in all tumors than that in the associated normal tissues (other than LAML, BRCA, and PRAD).

## Slide 4
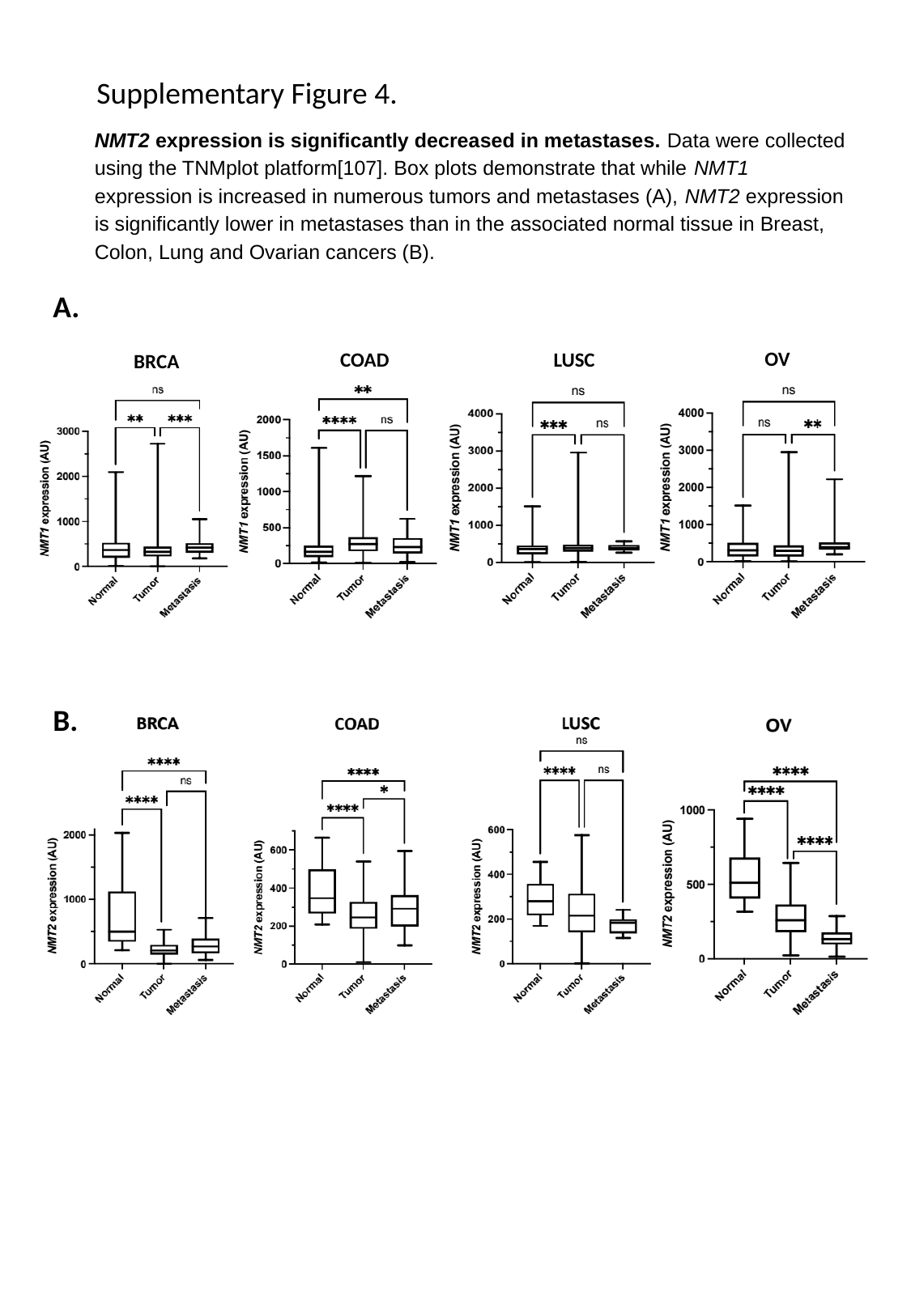

Supplementary Figure 4.
NMT2 expression is significantly decreased in metastases. Data were collected using the TNMplot platform[107]. Box plots demonstrate that while NMT1 expression is increased in numerous tumors and metastases (A), NMT2 expression is significantly lower in metastases than in the associated normal tissue in Breast, Colon, Lung and Ovarian cancers (B).
A.
OV
LUSC
COAD
BRCA
B.

## Slide 5
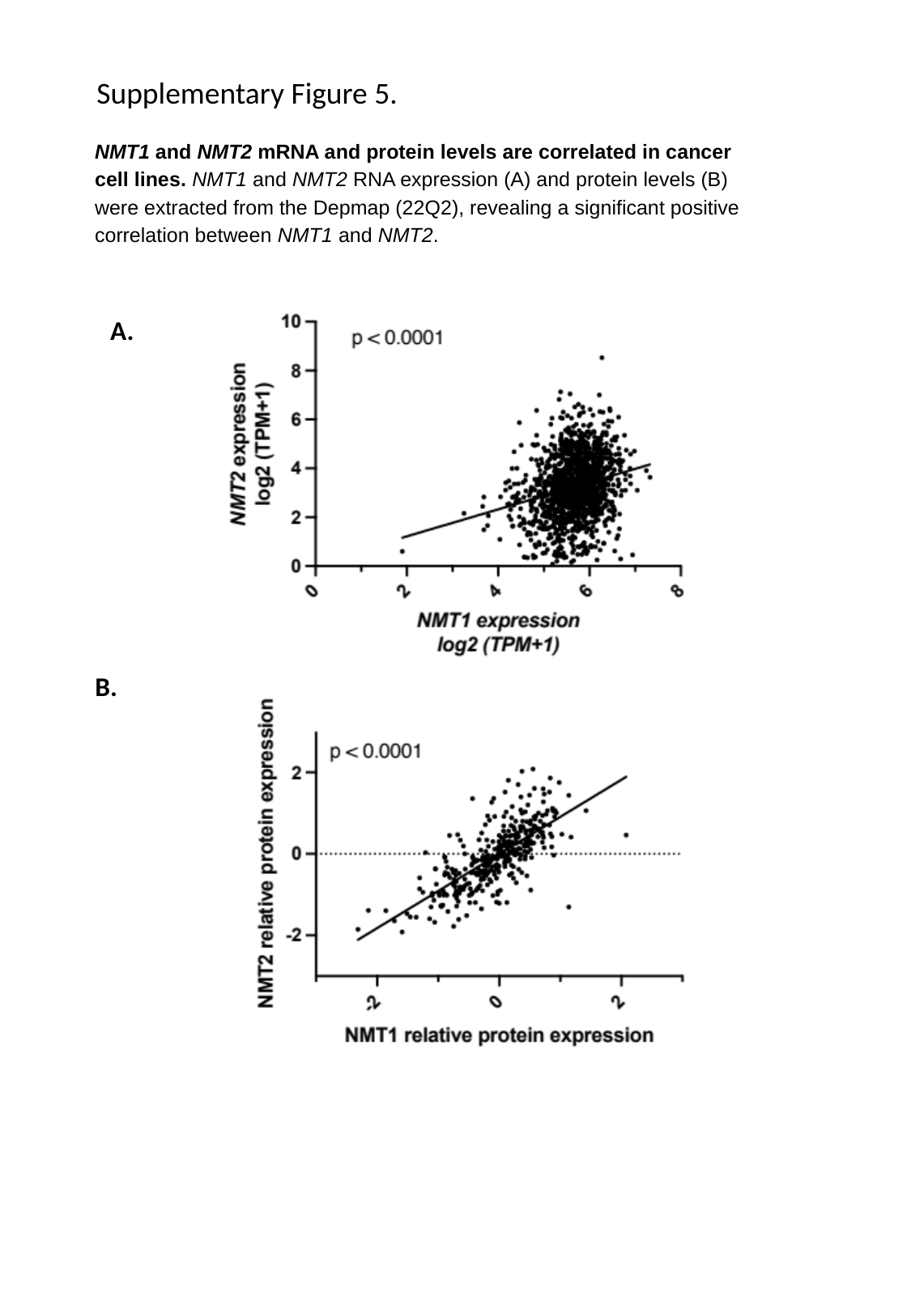

Supplementary Figure 5.
NMT1 and NMT2 mRNA and protein levels are correlated in cancer cell lines. NMT1 and NMT2 RNA expression (A) and protein levels (B) were extracted from the Depmap (22Q2), revealing a significant positive correlation between NMT1 and NMT2.
A.
B.

## Slide 6
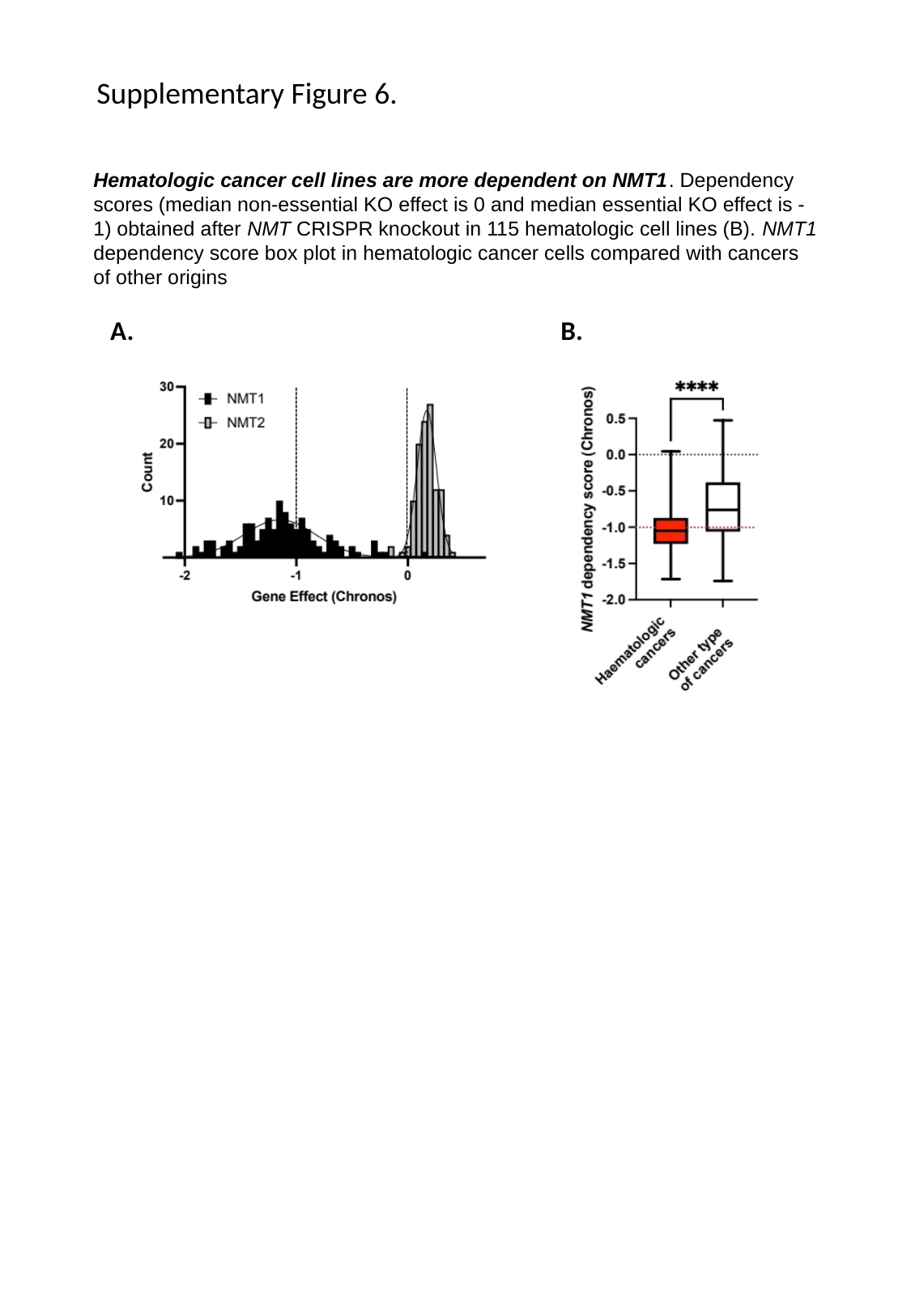

Supplementary Figure 6.
Hematologic cancer cell lines are more dependent on NMT1. Dependency scores (median non-essential KO effect is 0 and median essential KO effect is -1) obtained after NMT CRISPR knockout in 115 hematologic cell lines (B). NMT1 dependency score box plot in hematologic cancer cells compared with cancers of other origins
A.
B.

## Slide 7
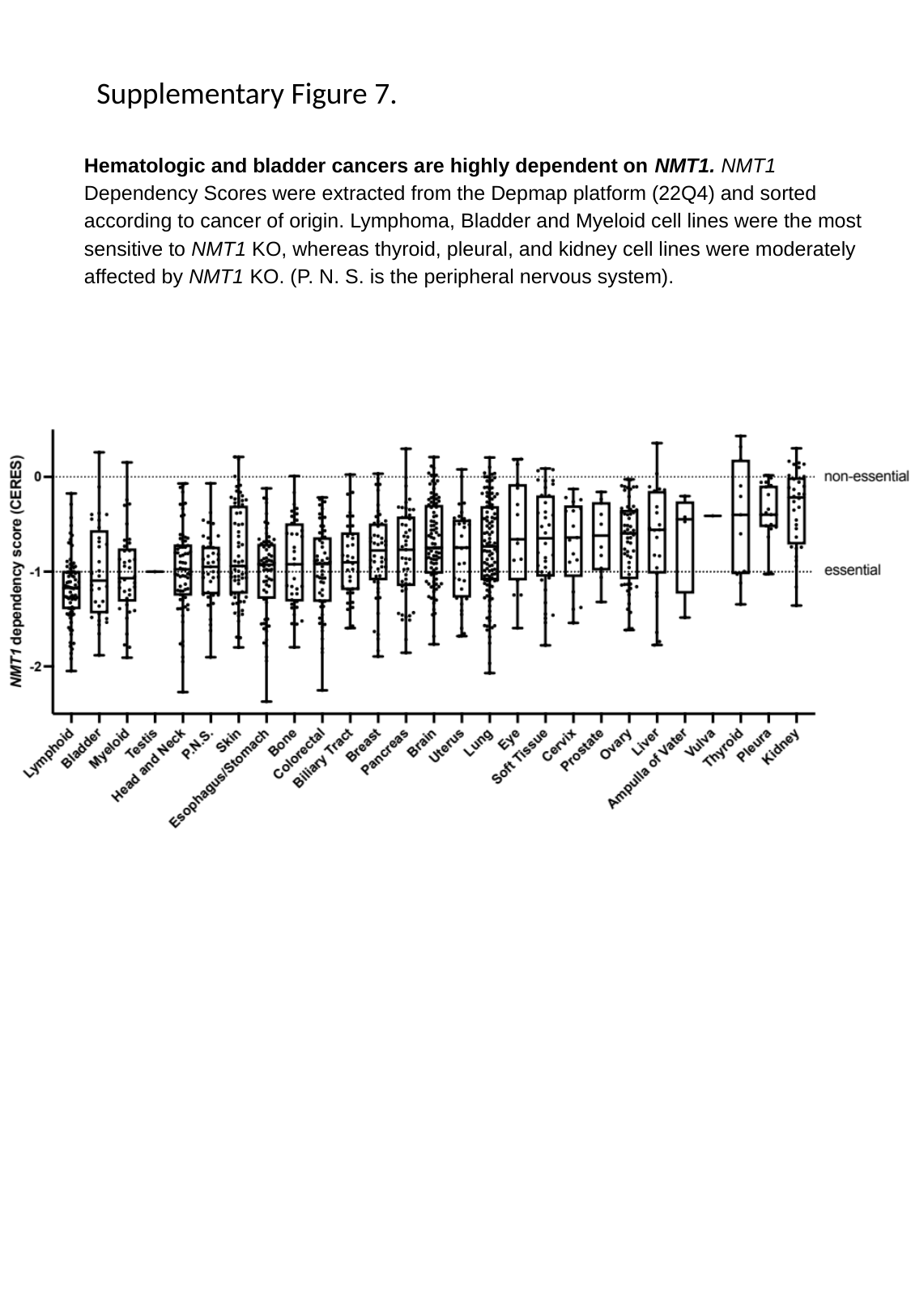

Supplementary Figure 7.
Hematologic and bladder cancers are highly dependent on NMT1. NMT1 Dependency Scores were extracted from the Depmap platform (22Q4) and sorted according to cancer of origin. Lymphoma, Bladder and Myeloid cell lines were the most sensitive to NMT1 KO, whereas thyroid, pleural, and kidney cell lines were moderately affected by NMT1 KO. (P. N. S. is the peripheral nervous system).

## Slide 8
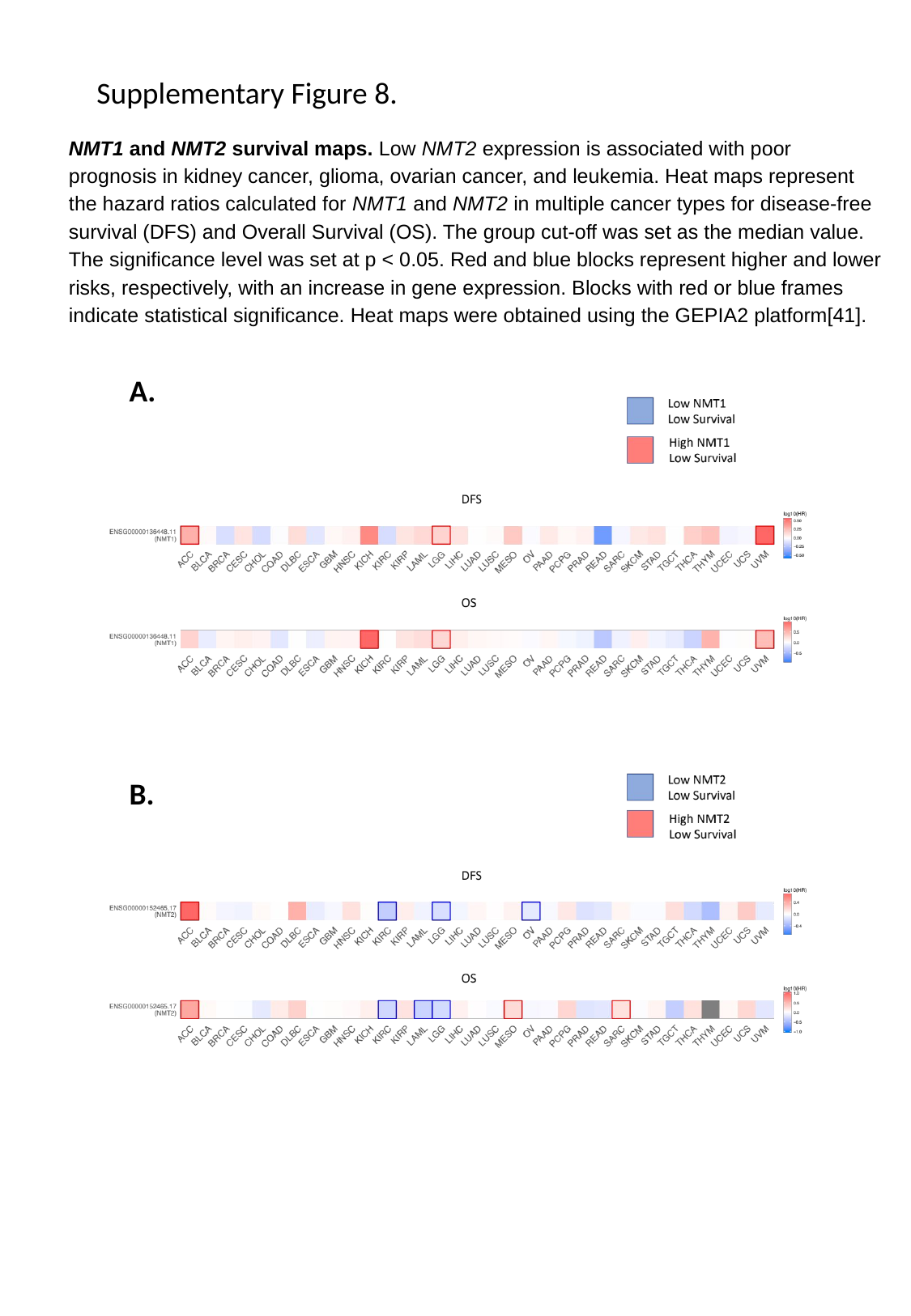

Supplementary Figure 8.
NMT1 and NMT2 survival maps. Low NMT2 expression is associated with poor prognosis in kidney cancer, glioma, ovarian cancer, and leukemia. Heat maps represent the hazard ratios calculated for NMT1 and NMT2 in multiple cancer types for disease-free survival (DFS) and Overall Survival (OS). The group cut-off was set as the median value. The significance level was set at p < 0.05. Red and blue blocks represent higher and lower risks, respectively, with an increase in gene expression. Blocks with red or blue frames indicate statistical significance. Heat maps were obtained using the GEPIA2 platform[41].
A.
B.

## Slide 9
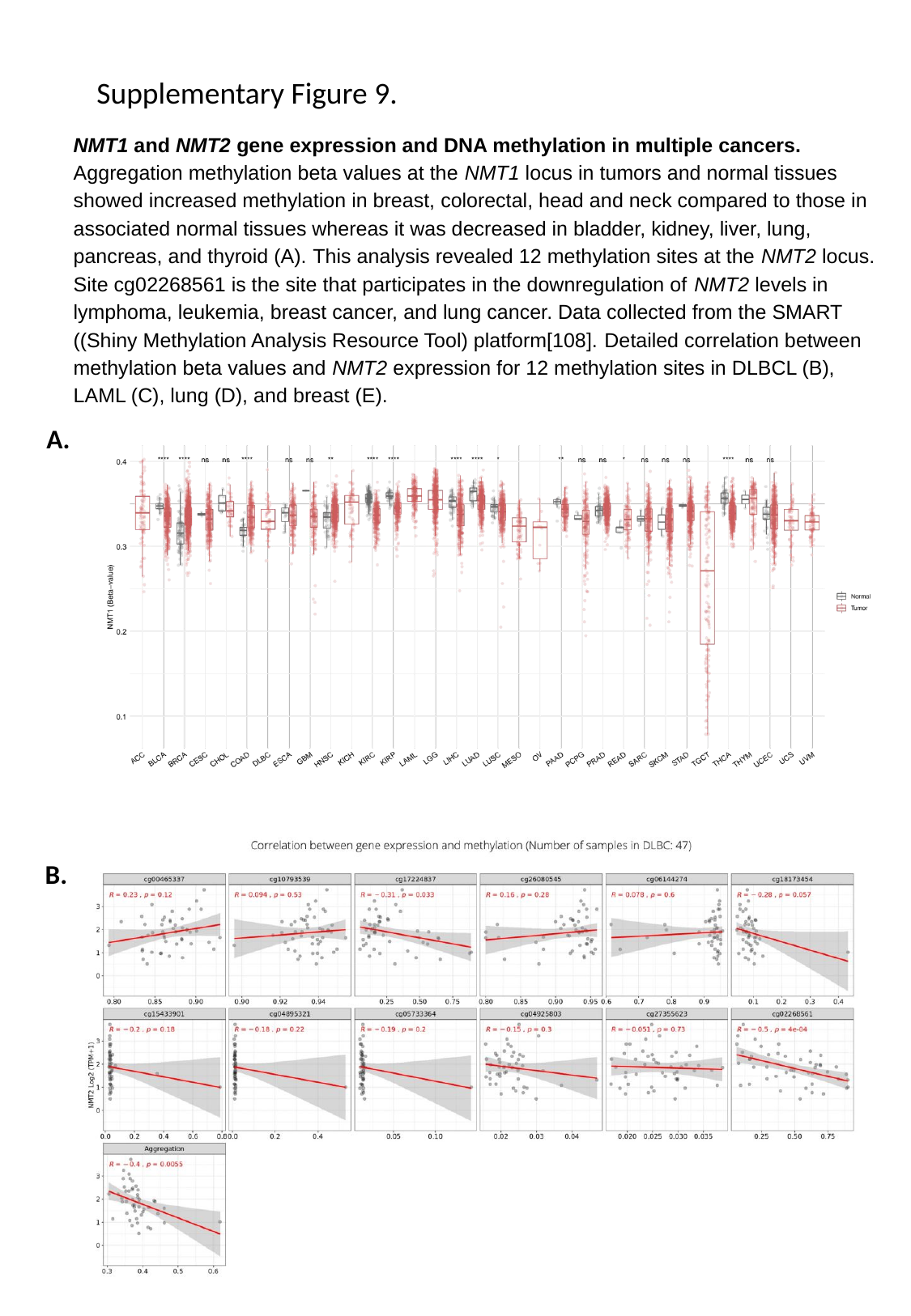

Supplementary Figure 9.
NMT1 and NMT2 gene expression and DNA methylation in multiple cancers. Aggregation methylation beta values at the NMT1 locus in tumors and normal tissues showed increased methylation in breast, colorectal, head and neck compared to those in associated normal tissues whereas it was decreased in bladder, kidney, liver, lung, pancreas, and thyroid (A). This analysis revealed 12 methylation sites at the NMT2 locus. Site cg02268561 is the site that participates in the downregulation of NMT2 levels in lymphoma, leukemia, breast cancer, and lung cancer. Data collected from the SMART ((Shiny Methylation Analysis Resource Tool) platform[108]. Detailed correlation between methylation beta values and NMT2 expression for 12 methylation sites in DLBCL (B), LAML (C), lung (D), and breast (E).
A.
B.

## Slide 10
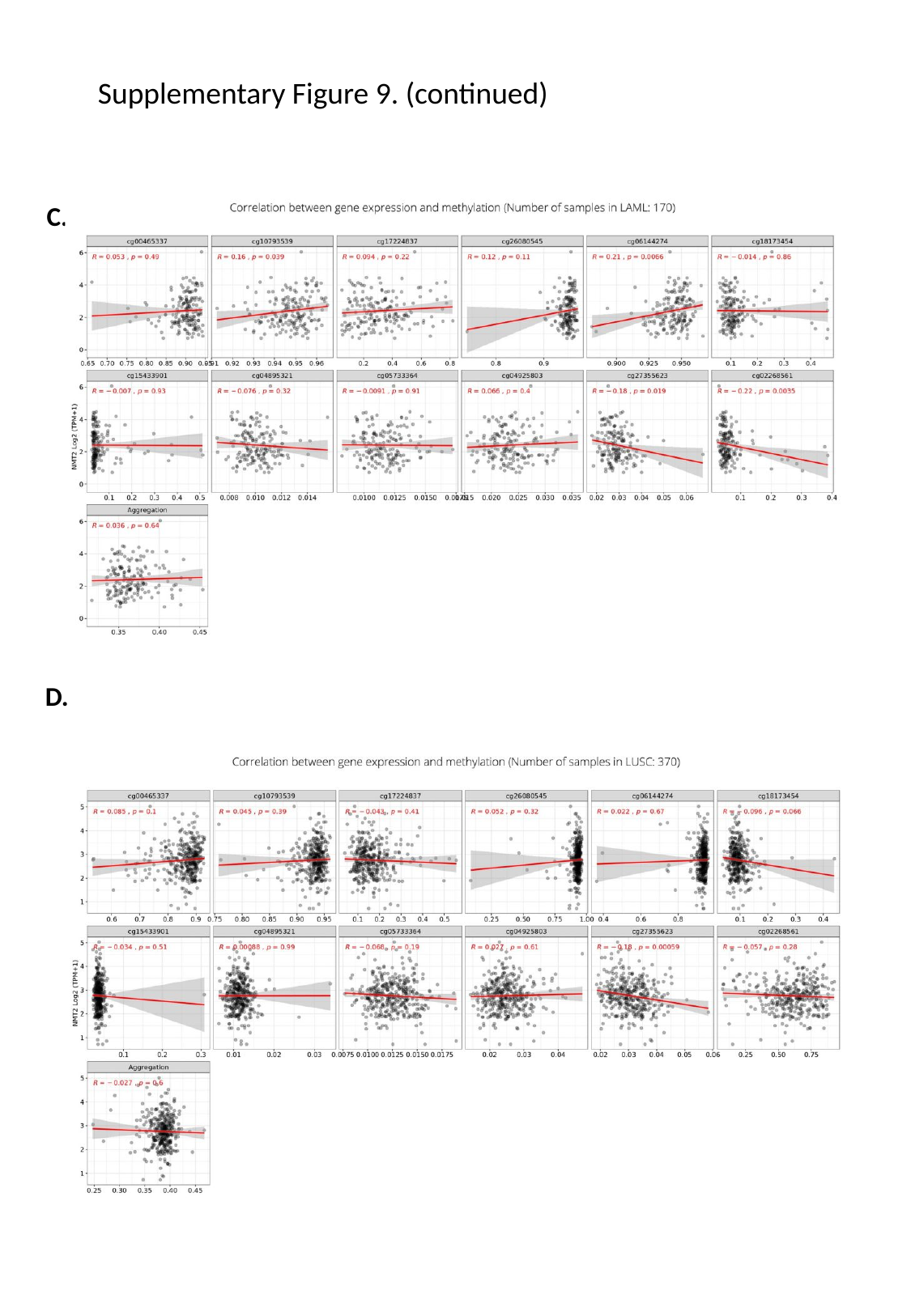

Supplementary Figure 9. (continued)
C.
D.

## Slide 11
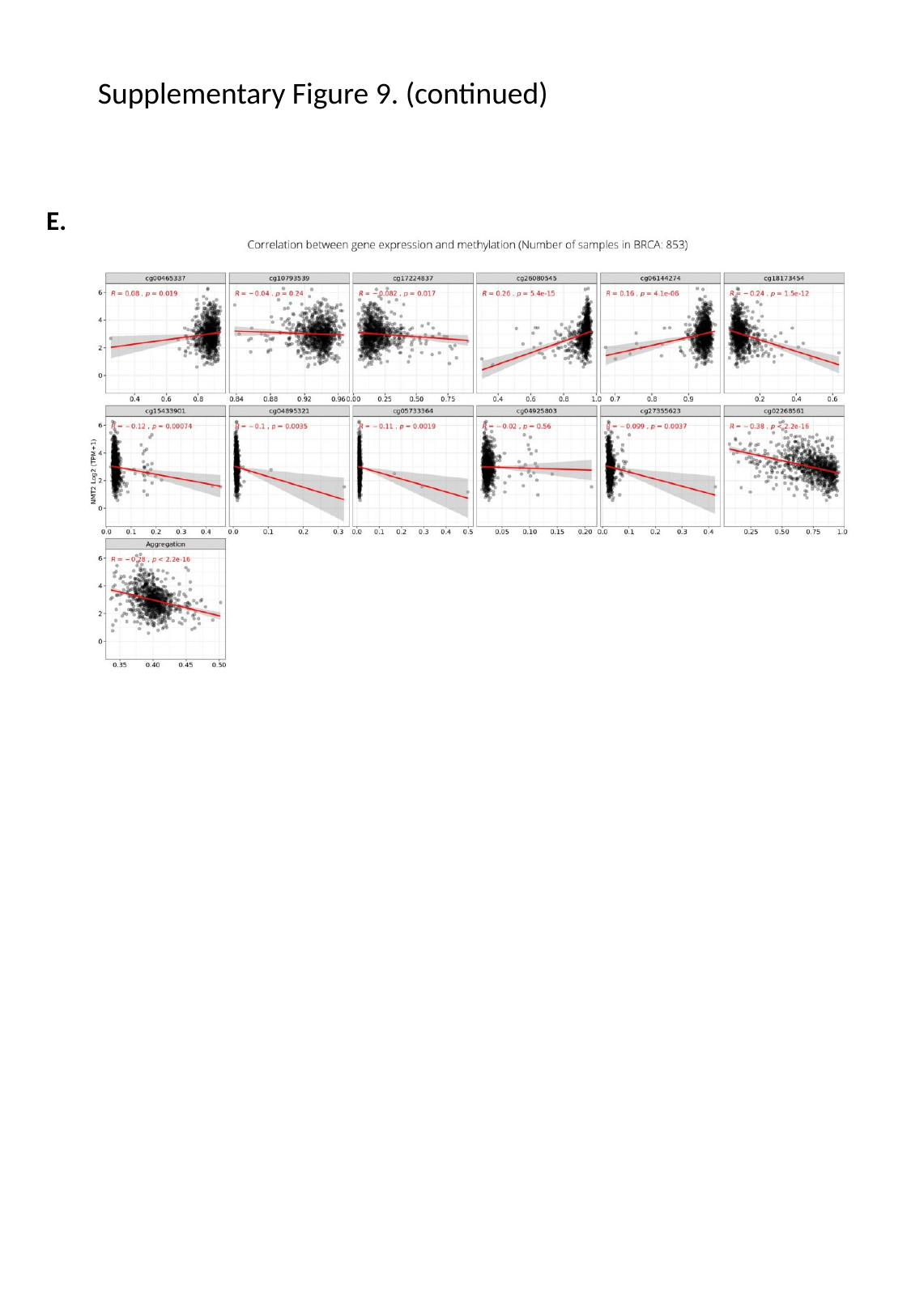

Supplementary Figure 9. (continued)
E.

## Slide 12
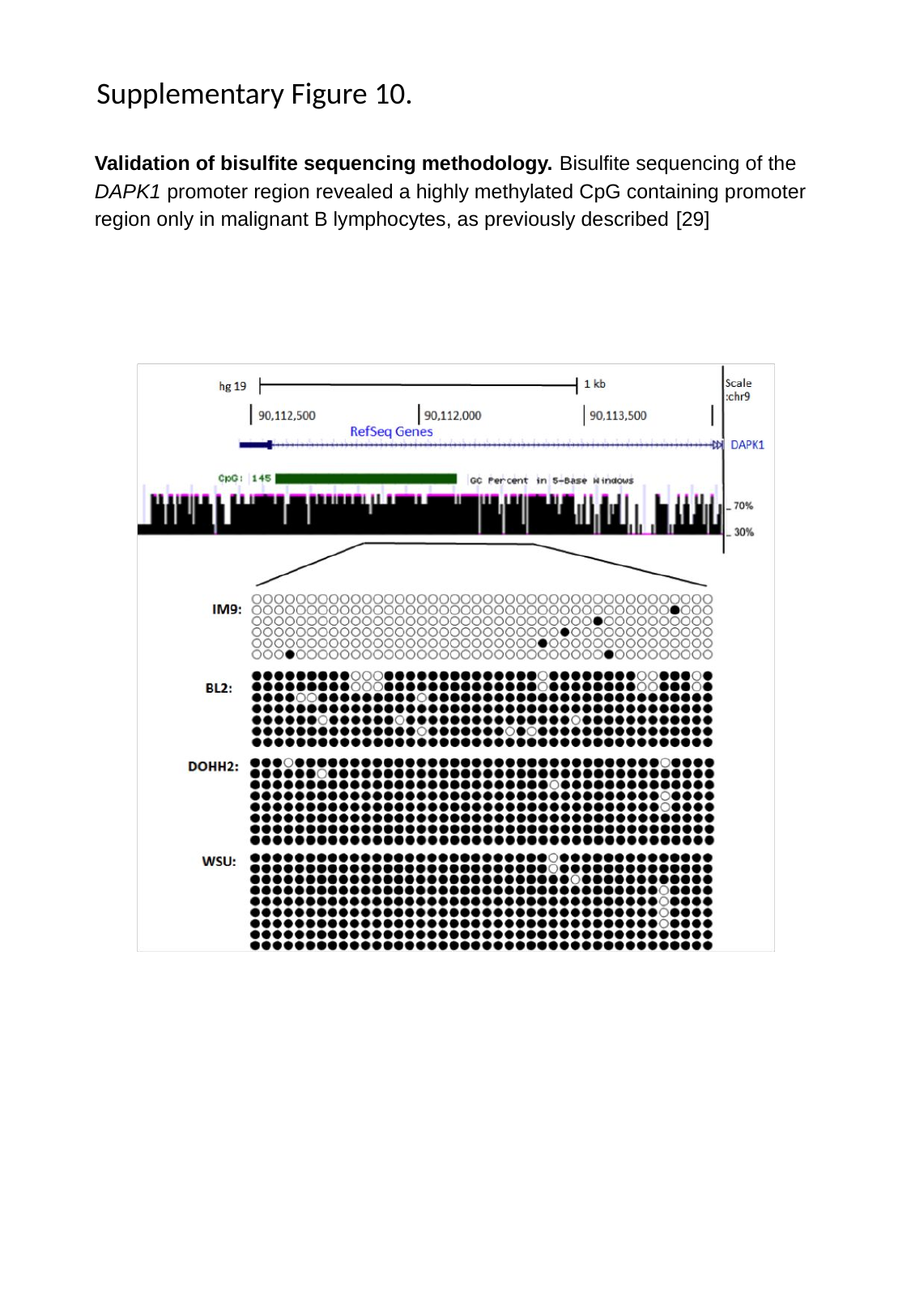

Supplementary Figure 10.
Validation of bisulfite sequencing methodology. Bisulfite sequencing of the DAPK1 promoter region revealed a highly methylated CpG containing promoter region only in malignant B lymphocytes, as previously described [29]

## Slide 13
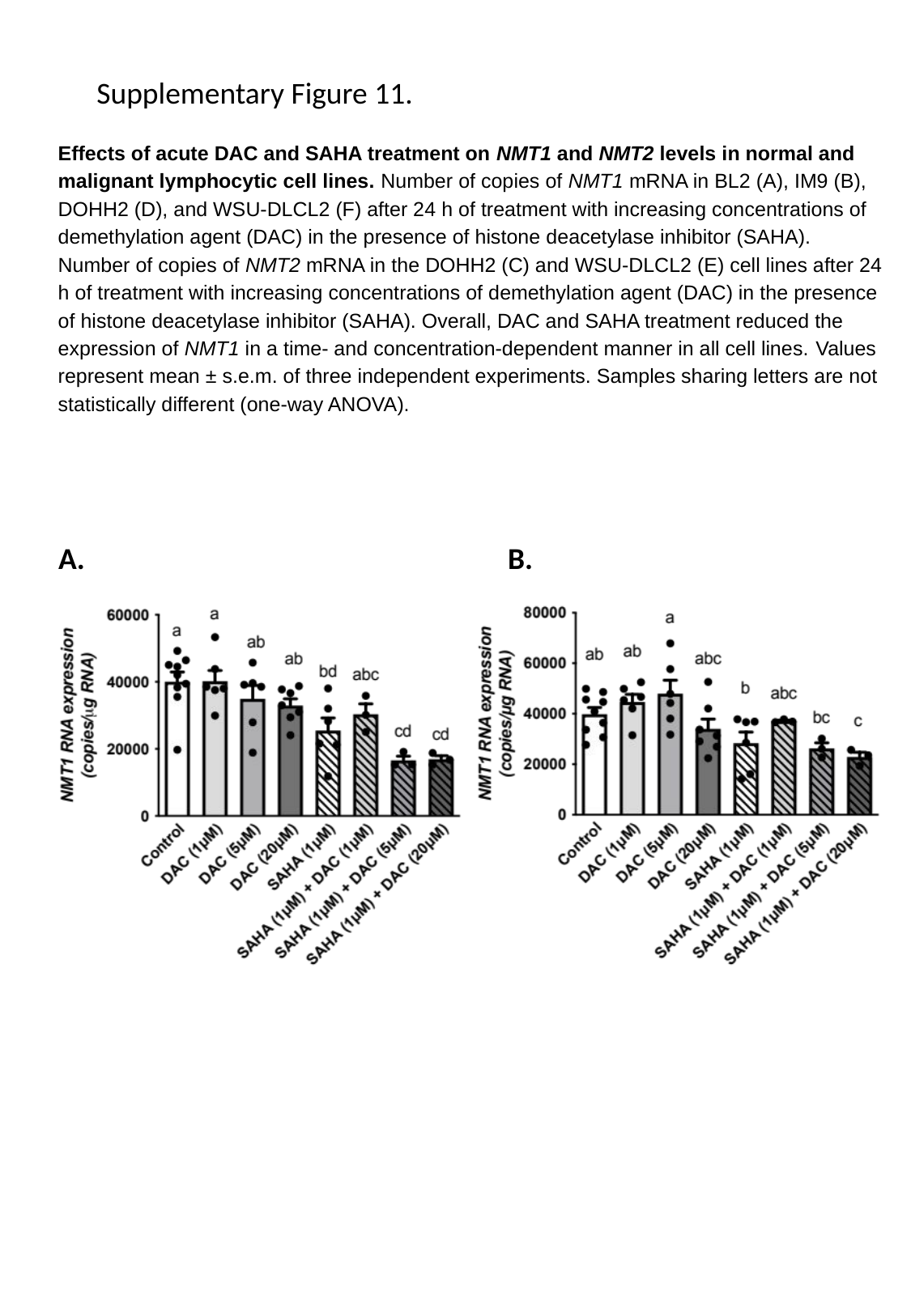

Supplementary Figure 11.
Effects of acute DAC and SAHA treatment on NMT1 and NMT2 levels in normal and malignant lymphocytic cell lines. Number of copies of NMT1 mRNA in BL2 (A), IM9 (B), DOHH2 (D), and WSU-DLCL2 (F) after 24 h of treatment with increasing concentrations of demethylation agent (DAC) in the presence of histone deacetylase inhibitor (SAHA). Number of copies of NMT2 mRNA in the DOHH2 (C) and WSU-DLCL2 (E) cell lines after 24 h of treatment with increasing concentrations of demethylation agent (DAC) in the presence of histone deacetylase inhibitor (SAHA). Overall, DAC and SAHA treatment reduced the expression of NMT1 in a time- and concentration-dependent manner in all cell lines. Values represent mean ± s.e.m. of three independent experiments. Samples sharing letters are not statistically different (one-way ANOVA).
A.
B.

## Slide 14
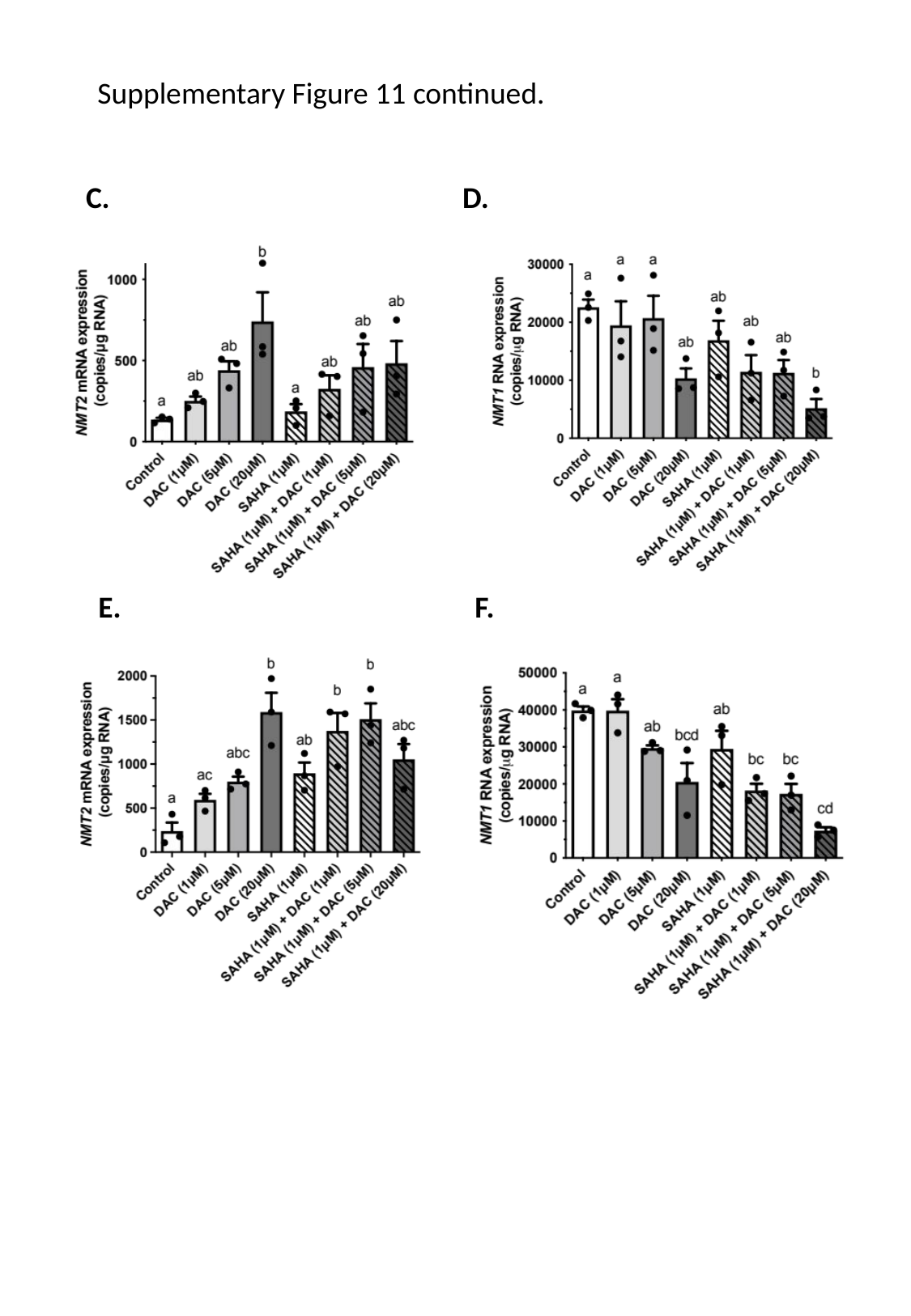

Supplementary Figure 11 continued.
C.
D.
E.
F.

## Slide 15
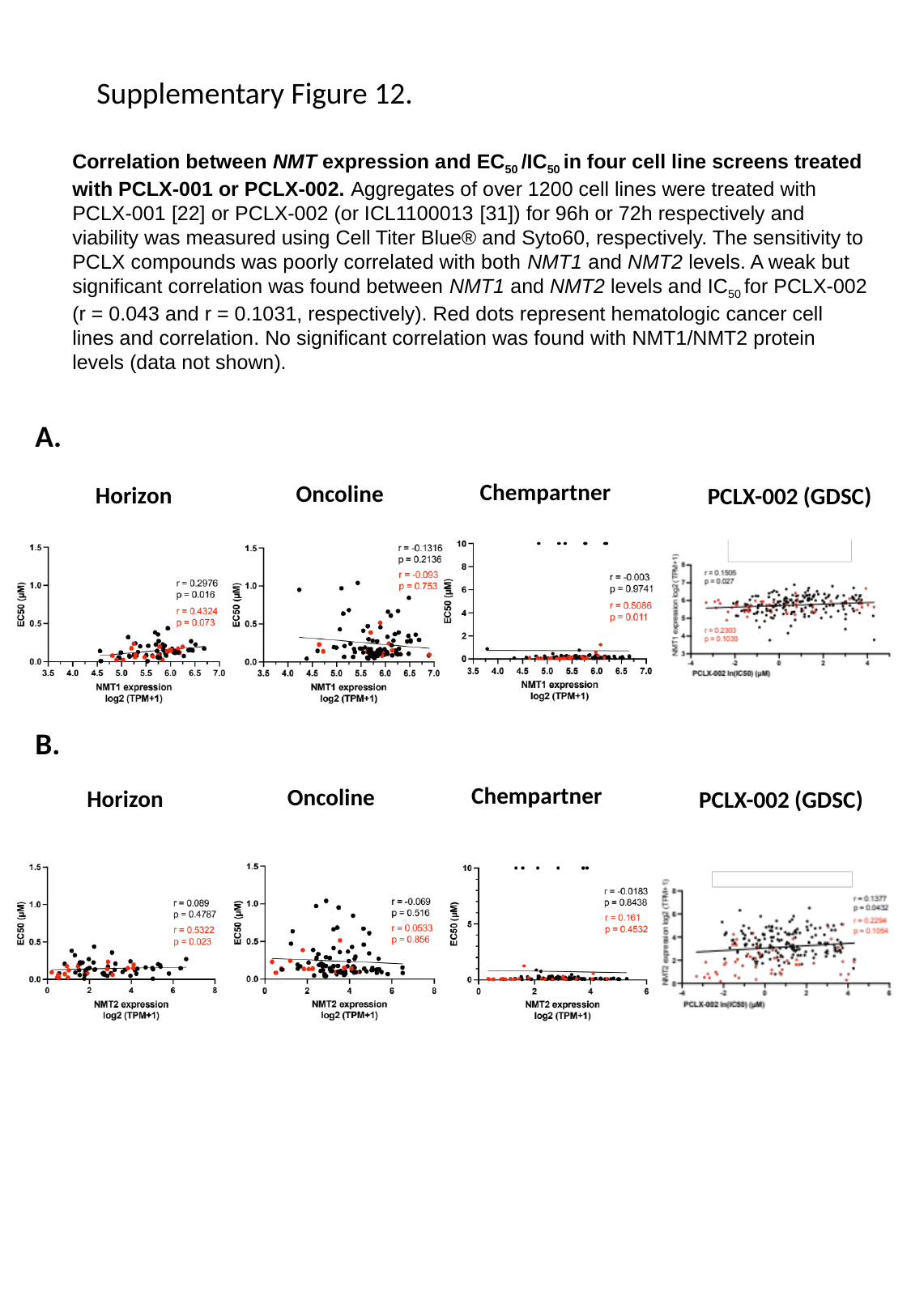

Supplementary Figure 12.
Correlation between NMT expression and EC50 /IC50 in four cell line screens treated with PCLX-001 or PCLX-002. Aggregates of over 1200 cell lines were treated with PCLX-001 [22] or PCLX-002 (or ICL1100013 [31]) for 96h or 72h respectively and viability was measured using Cell Titer Blue® and Syto60, respectively. The sensitivity to PCLX compounds was poorly correlated with both NMT1 and NMT2 levels. A weak but significant correlation was found between NMT1 and NMT2 levels and IC50 for PCLX-002 (r = 0.043 and r = 0.1031, respectively). Red dots represent hematologic cancer cell lines and correlation. No significant correlation was found with NMT1/NMT2 protein levels (data not shown).
A.
Chempartner
Oncoline
Horizon
PCLX-002 (GDSC)
B.
Chempartner
Oncoline
Horizon
PCLX-002 (GDSC)

## Slide 16
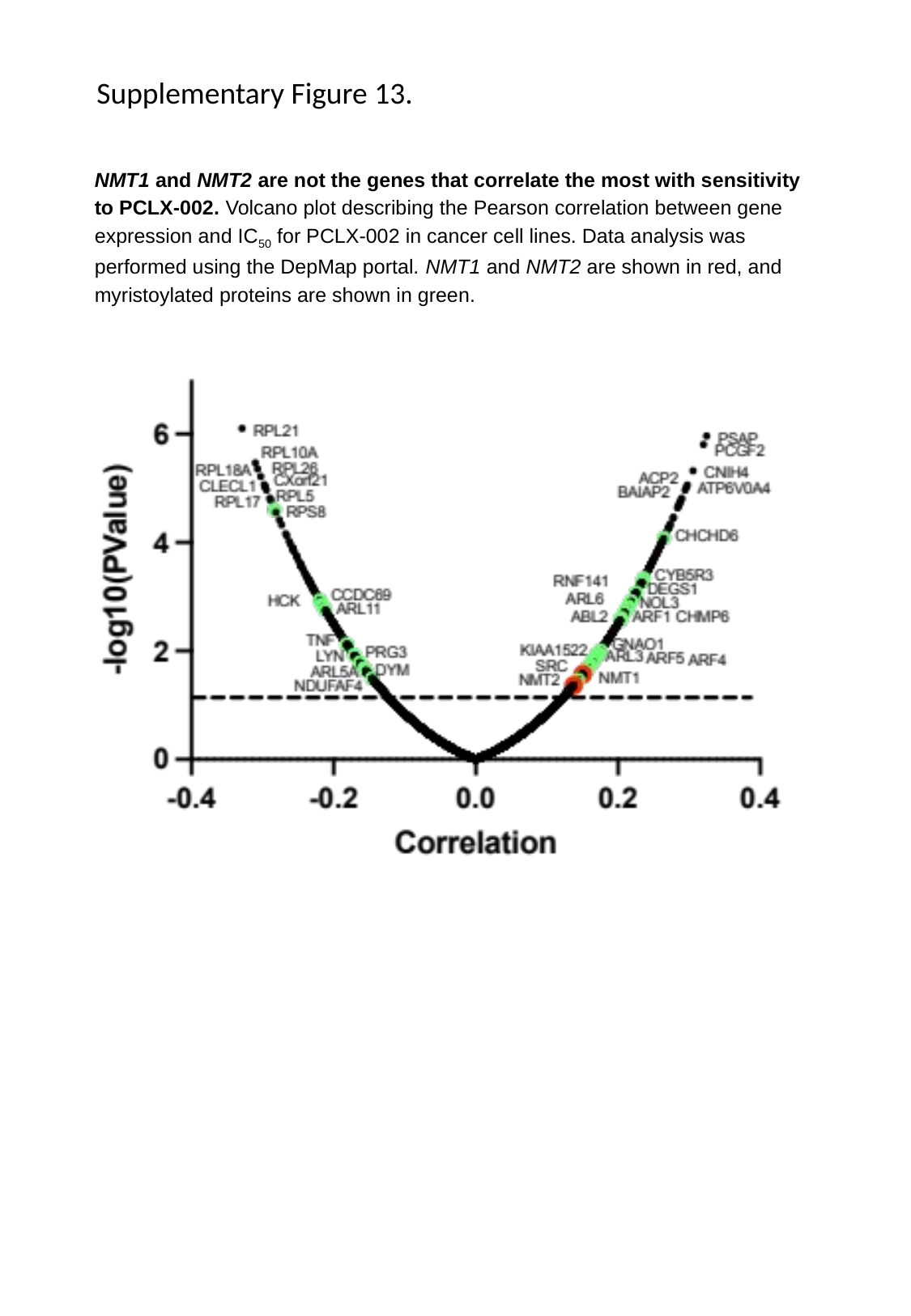

Supplementary Figure 13.
NMT1 and NMT2 are not the genes that correlate the most with sensitivity to PCLX-002. Volcano plot describing the Pearson correlation between gene expression and IC50 for PCLX-002 in cancer cell lines. Data analysis was performed using the DepMap portal. NMT1 and NMT2 are shown in red, and myristoylated proteins are shown in green.

## Slide 17
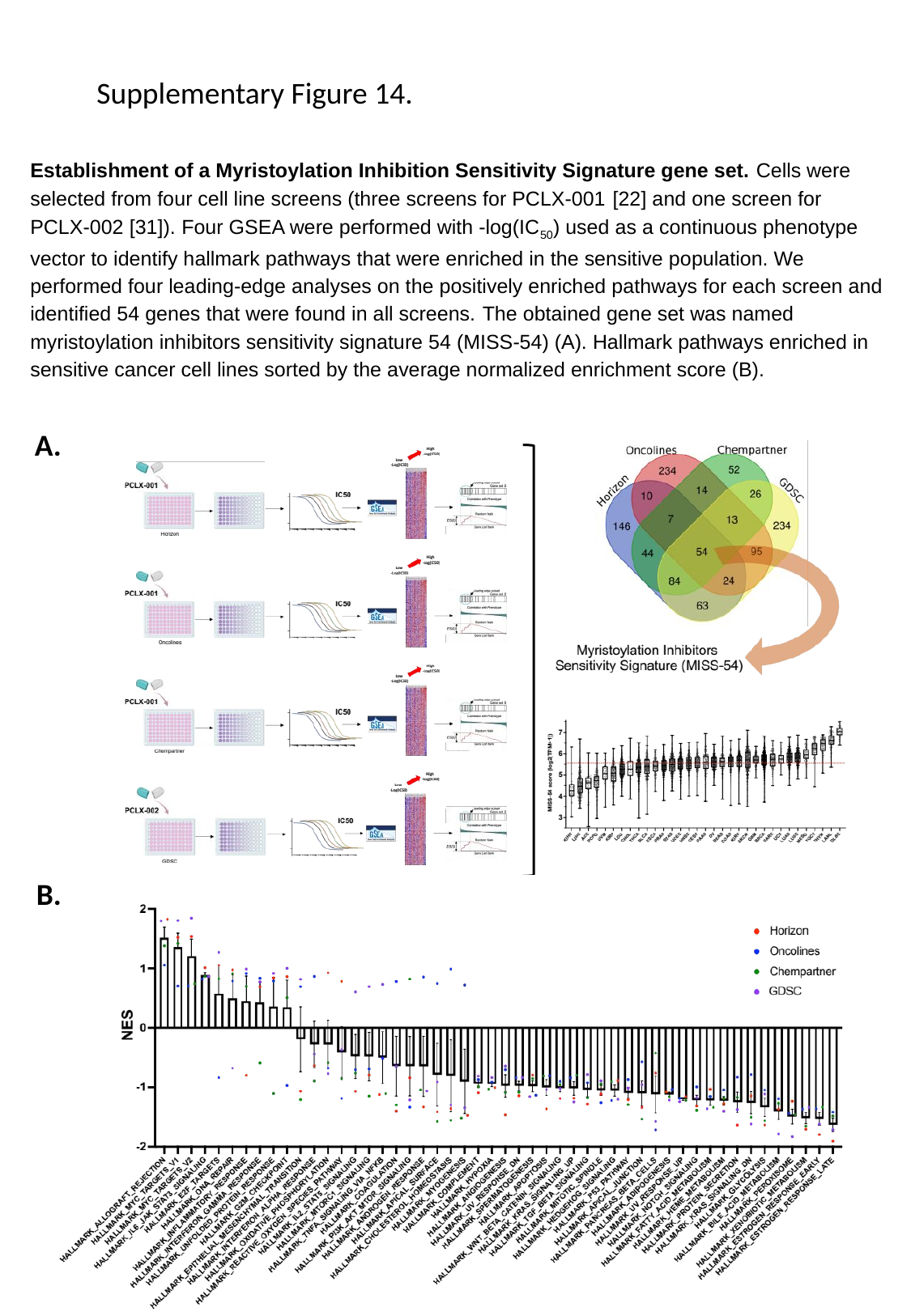

Supplementary Figure 14.
Establishment of a Myristoylation Inhibition Sensitivity Signature gene set. Cells were selected from four cell line screens (three screens for PCLX-001 [22] and one screen for PCLX-002 [31]). Four GSEA were performed with -log(IC50) used as a continuous phenotype vector to identify hallmark pathways that were enriched in the sensitive population. We performed four leading-edge analyses on the positively enriched pathways for each screen and identified 54 genes that were found in all screens. The obtained gene set was named myristoylation inhibitors sensitivity signature 54 (MISS-54) (A). Hallmark pathways enriched in sensitive cancer cell lines sorted by the average normalized enrichment score (B).
A.
B.

## Slide 18
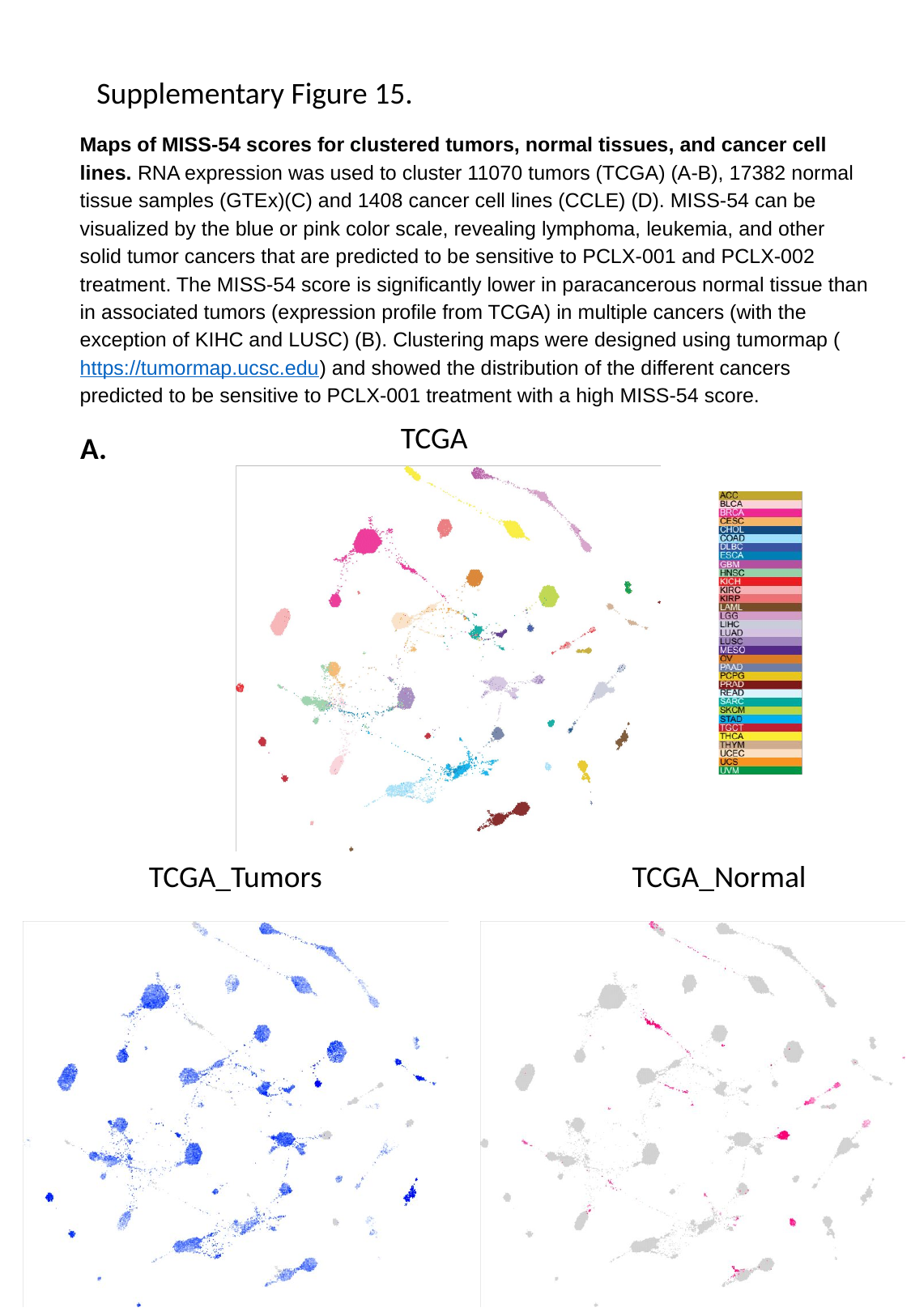

Supplementary Figure 15.
Maps of MISS-54 scores for clustered tumors, normal tissues, and cancer cell lines. RNA expression was used to cluster 11070 tumors (TCGA) (A-B), 17382 normal tissue samples (GTEx)(C) and 1408 cancer cell lines (CCLE) (D). MISS-54 can be visualized by the blue or pink color scale, revealing lymphoma, leukemia, and other solid tumor cancers that are predicted to be sensitive to PCLX-001 and PCLX-002 treatment. The MISS-54 score is significantly lower in paracancerous normal tissue than in associated tumors (expression profile from TCGA) in multiple cancers (with the exception of KIHC and LUSC) (B). Clustering maps were designed using tumormap (https://tumormap.ucsc.edu) and showed the distribution of the different cancers predicted to be sensitive to PCLX-001 treatment with a high MISS-54 score.
TCGA
A.
TCGA_Tumors
TCGA_Normal

## Slide 19
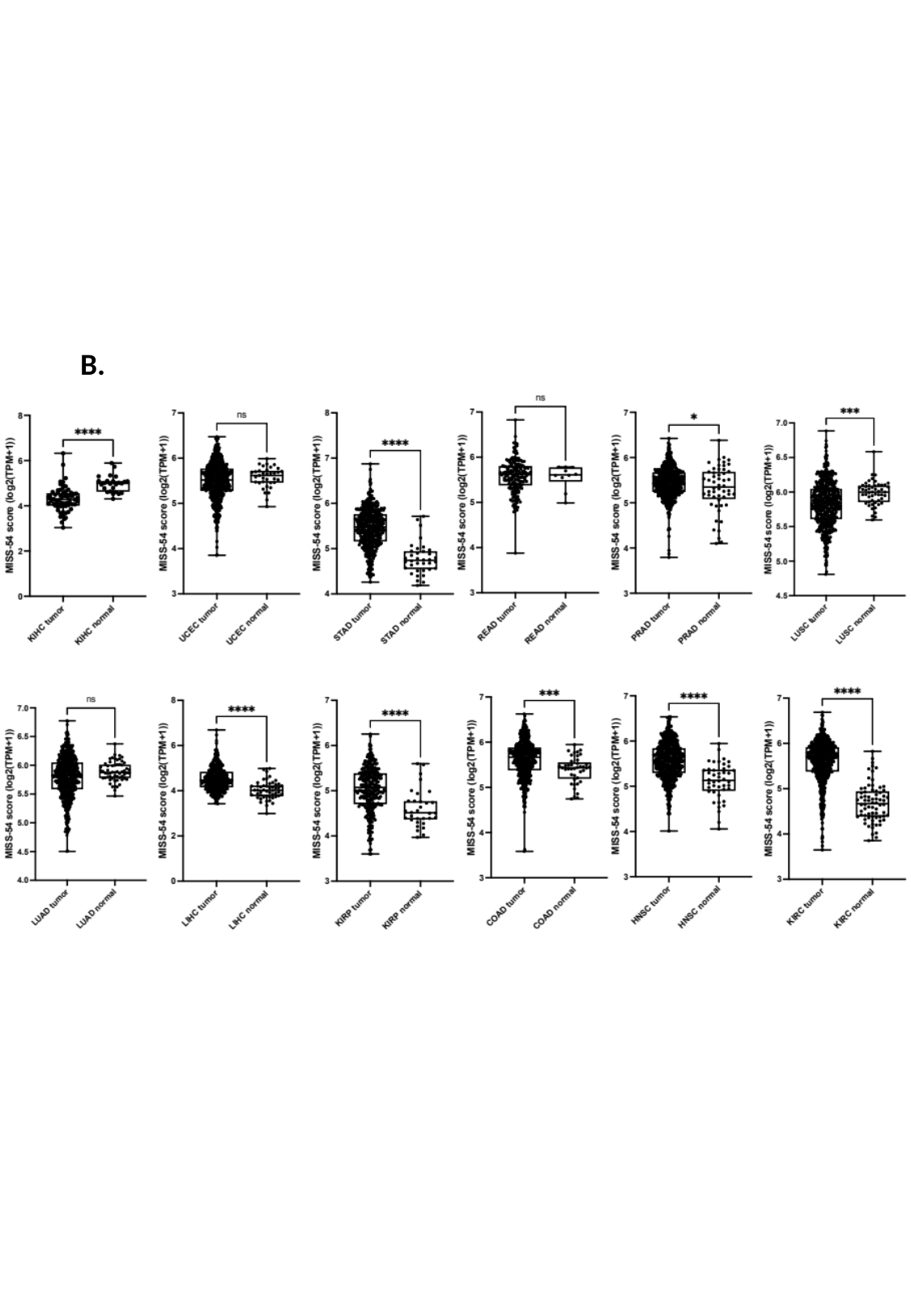

B.

## Slide 20
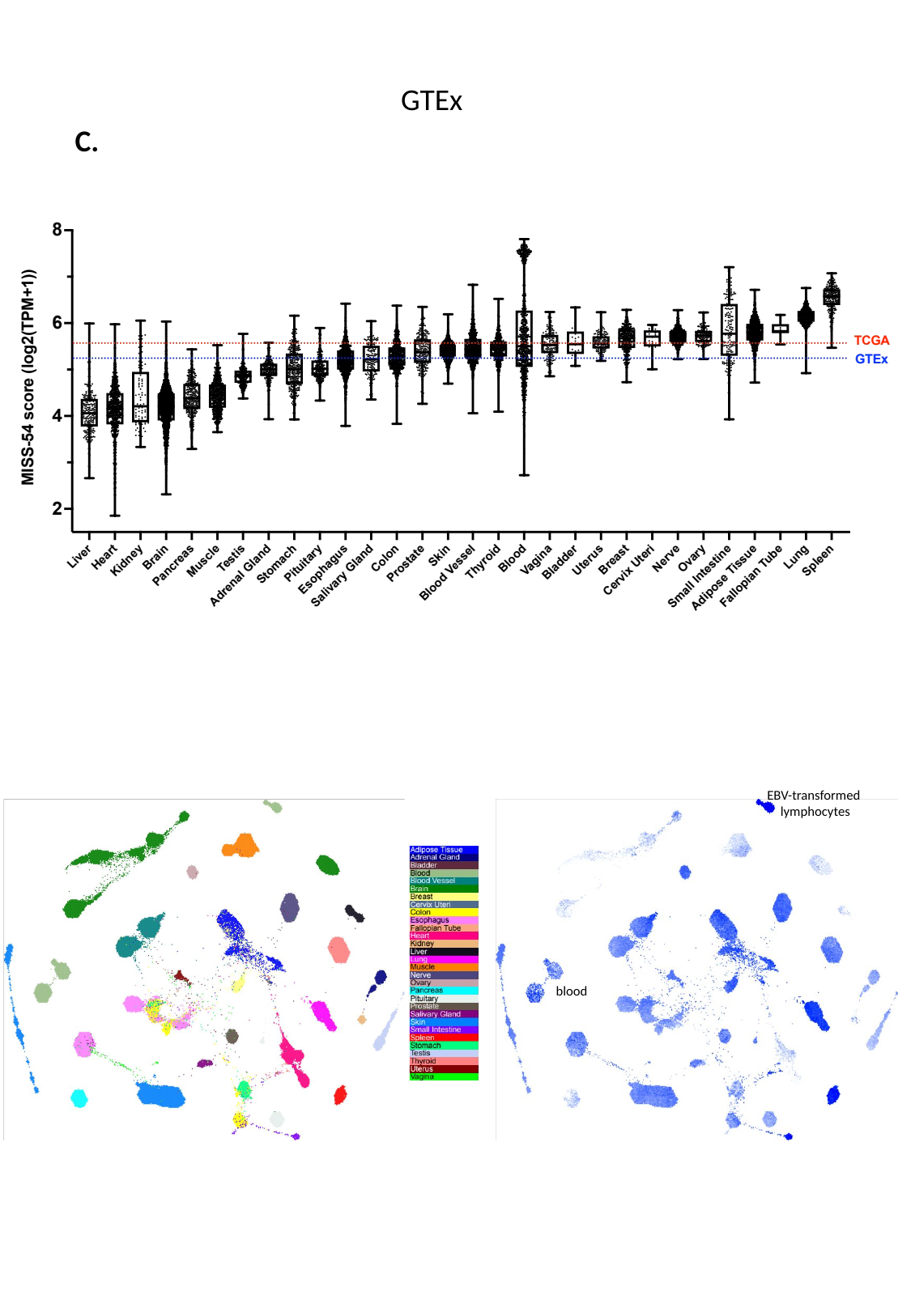

GTEx
C.
EBV-transformed
lymphocytes
blood

## Slide 21
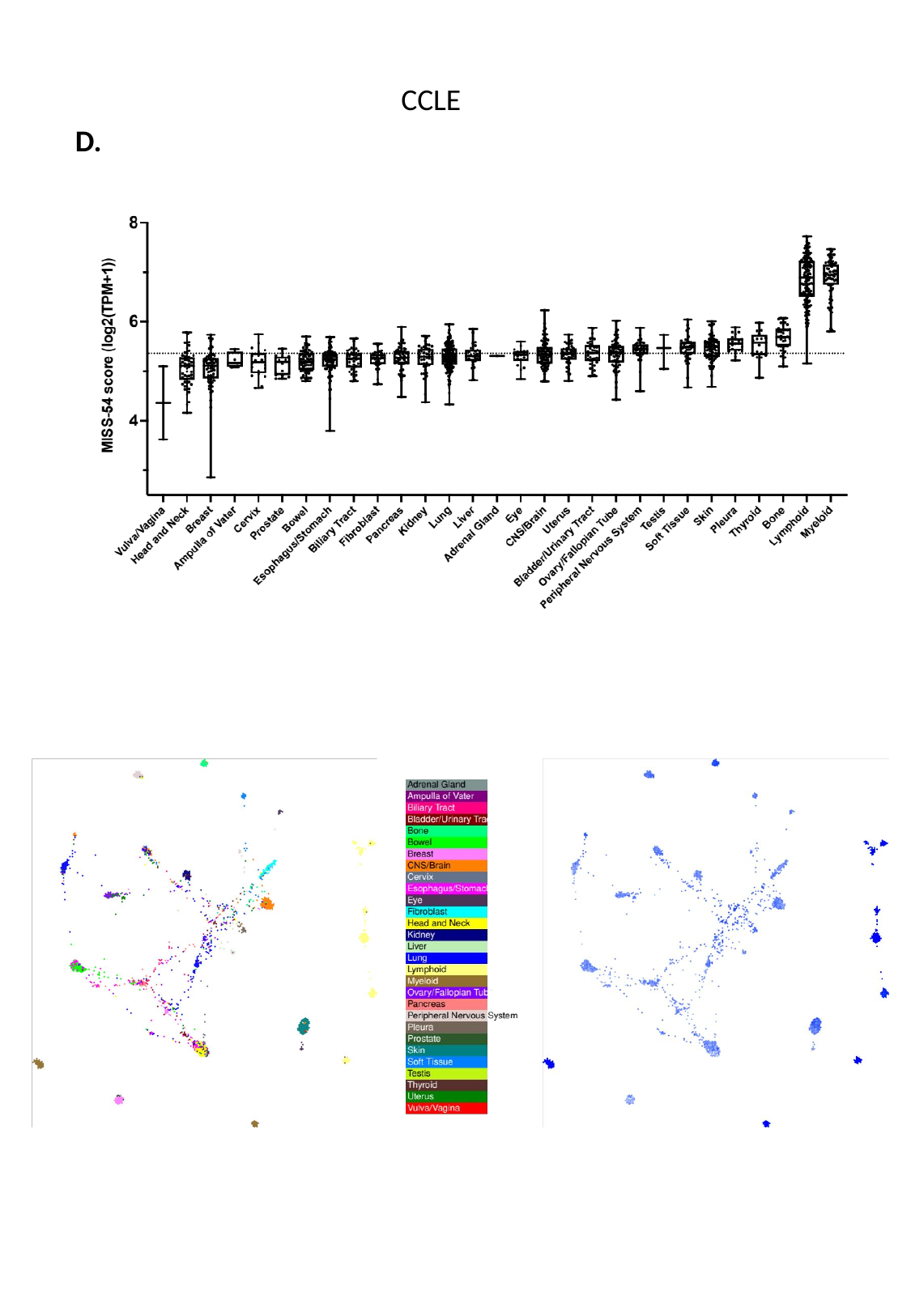

CCLE
D.

## Slide 22
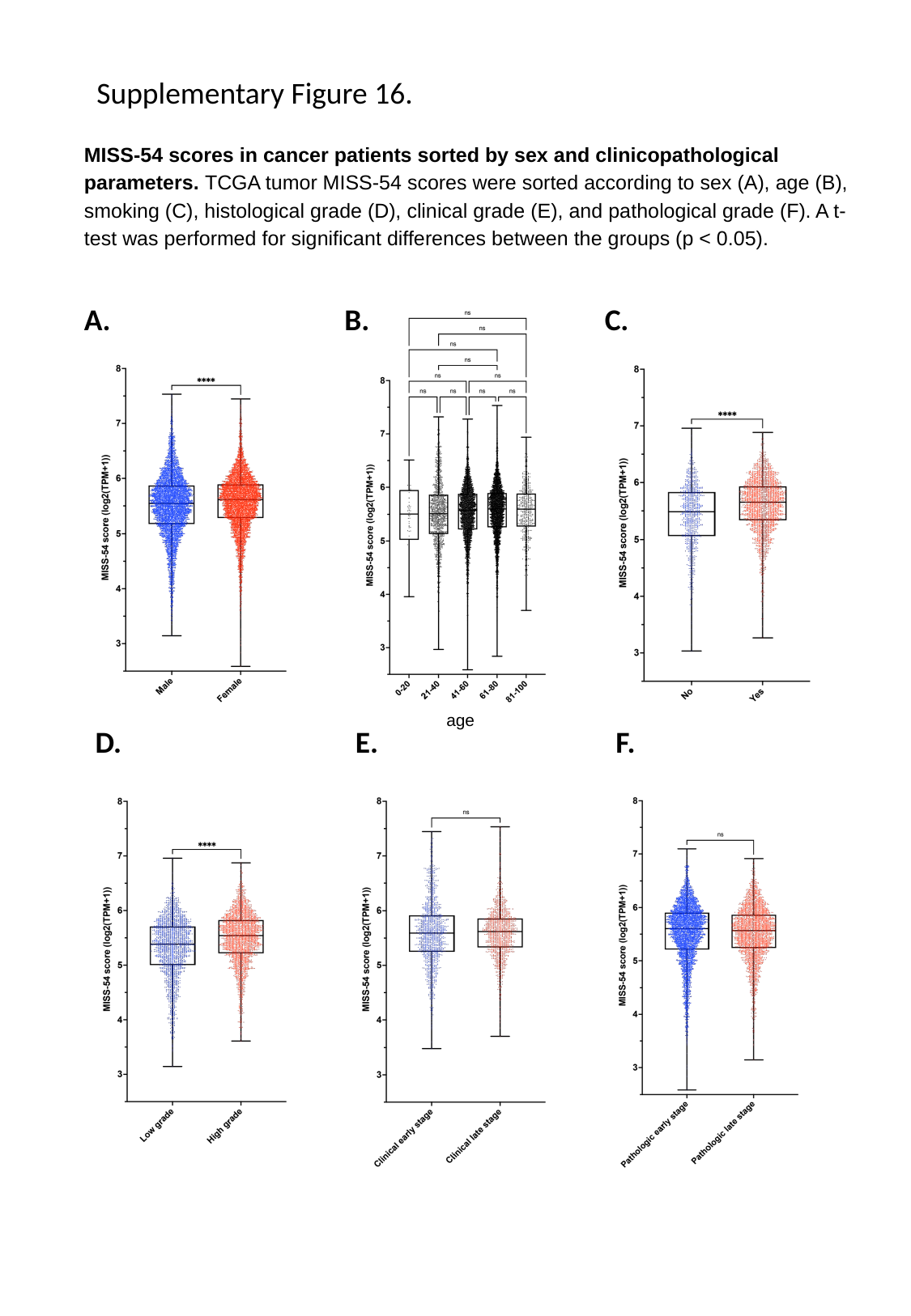

Supplementary Figure 16.
MISS-54 scores in cancer patients sorted by sex and clinicopathological parameters. TCGA tumor MISS-54 scores were sorted according to sex (A), age (B), smoking (C), histological grade (D), clinical grade (E), and pathological grade (F). A t-test was performed for significant differences between the groups (p < 0.05).
A.
B.
C.
age
D.
E.
F.

## Slide 23
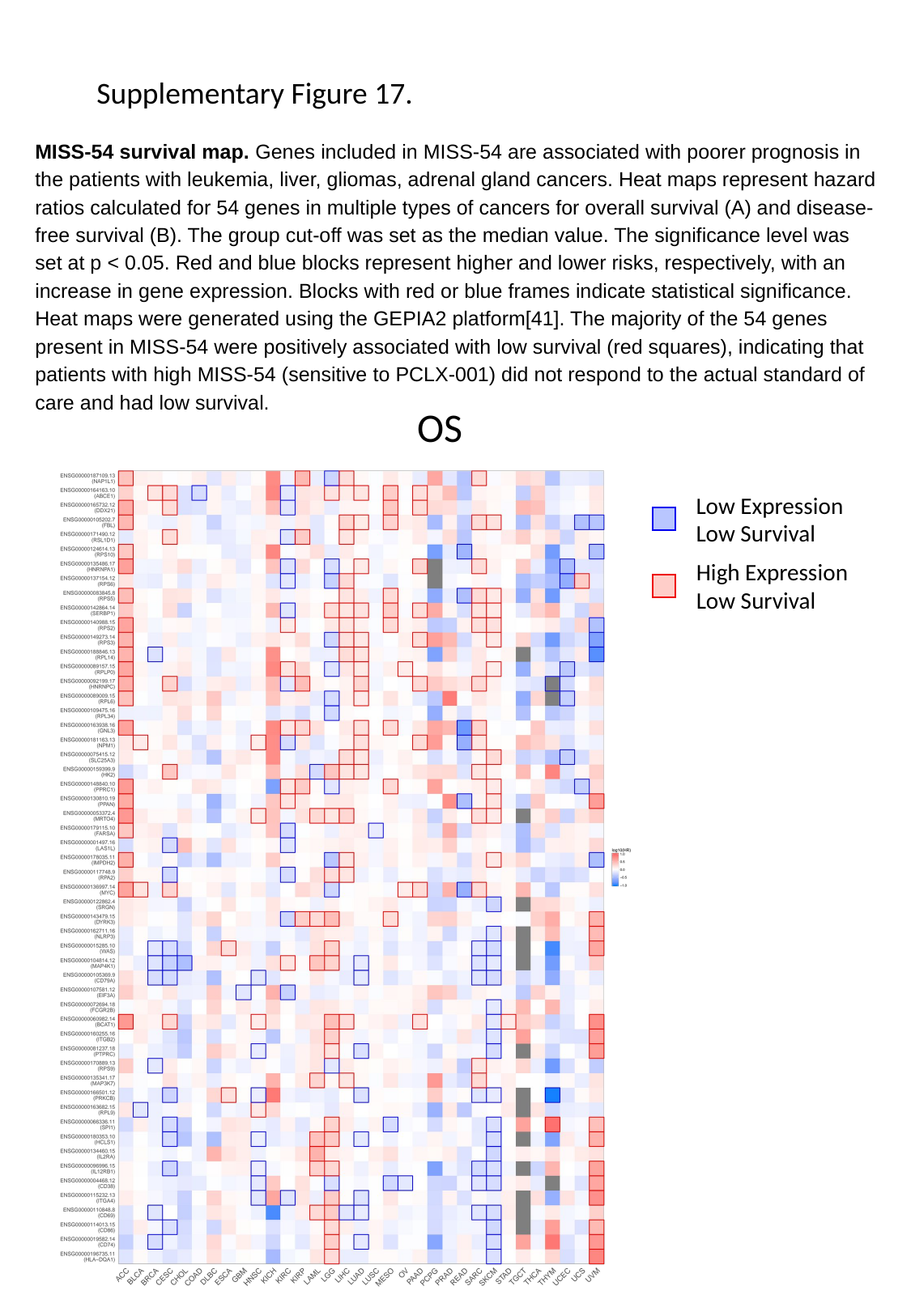

Supplementary Figure 17.
MISS-54 survival map. Genes included in MISS-54 are associated with poorer prognosis in the patients with leukemia, liver, gliomas, adrenal gland cancers. Heat maps represent hazard ratios calculated for 54 genes in multiple types of cancers for overall survival (A) and disease-free survival (B). The group cut-off was set as the median value. The significance level was set at p < 0.05. Red and blue blocks represent higher and lower risks, respectively, with an increase in gene expression. Blocks with red or blue frames indicate statistical significance. Heat maps were generated using the GEPIA2 platform[41]. The majority of the 54 genes present in MISS-54 were positively associated with low survival (red squares), indicating that patients with high MISS-54 (sensitive to PCLX-001) did not respond to the actual standard of care and had low survival.
OS
Low Expression
Low Survival
High Expression
Low Survival

## Slide 24
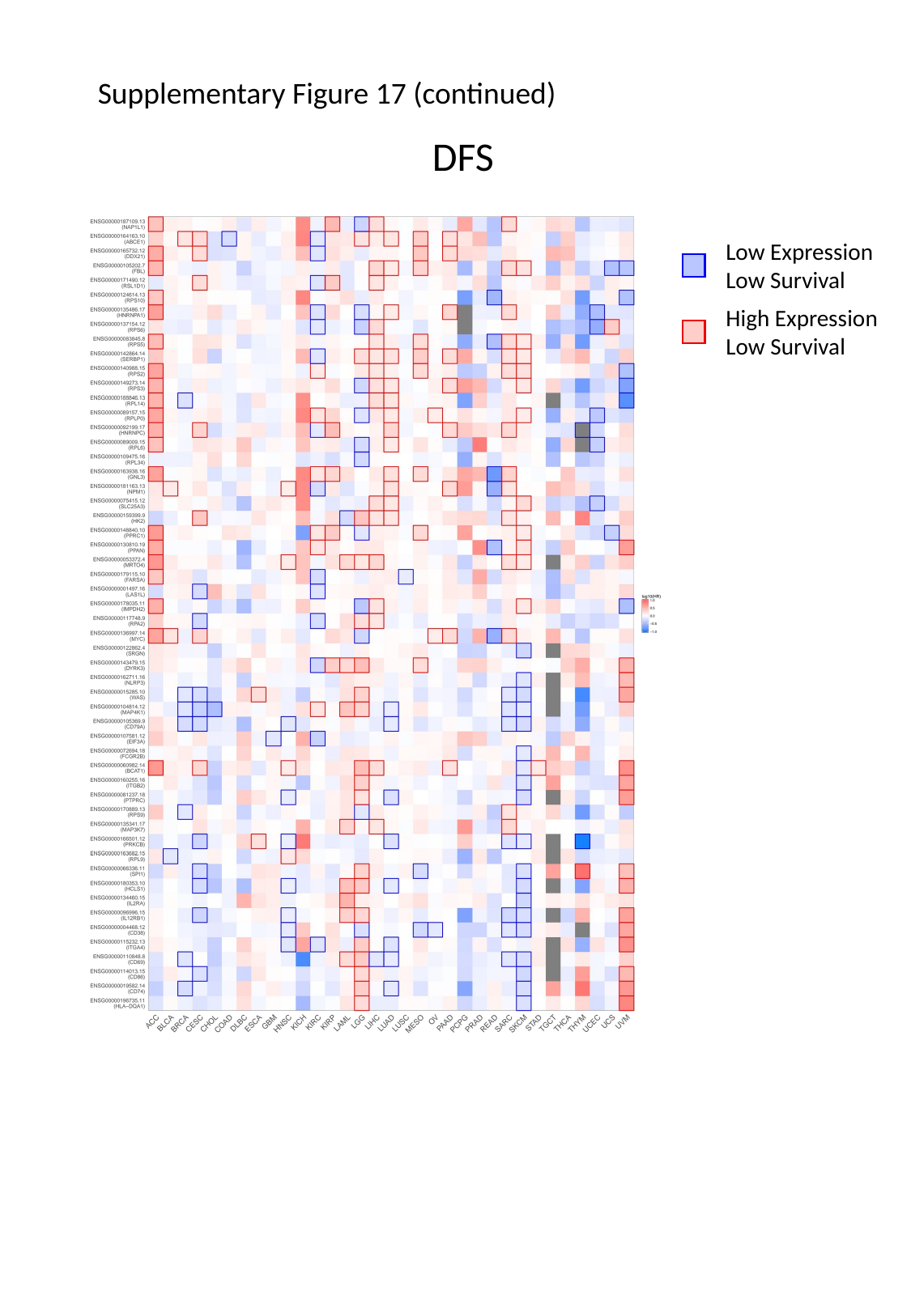

Supplementary Figure 17 (continued)
DFS
Low Expression
Low Survival
High Expression
Low Survival

## Slide 25
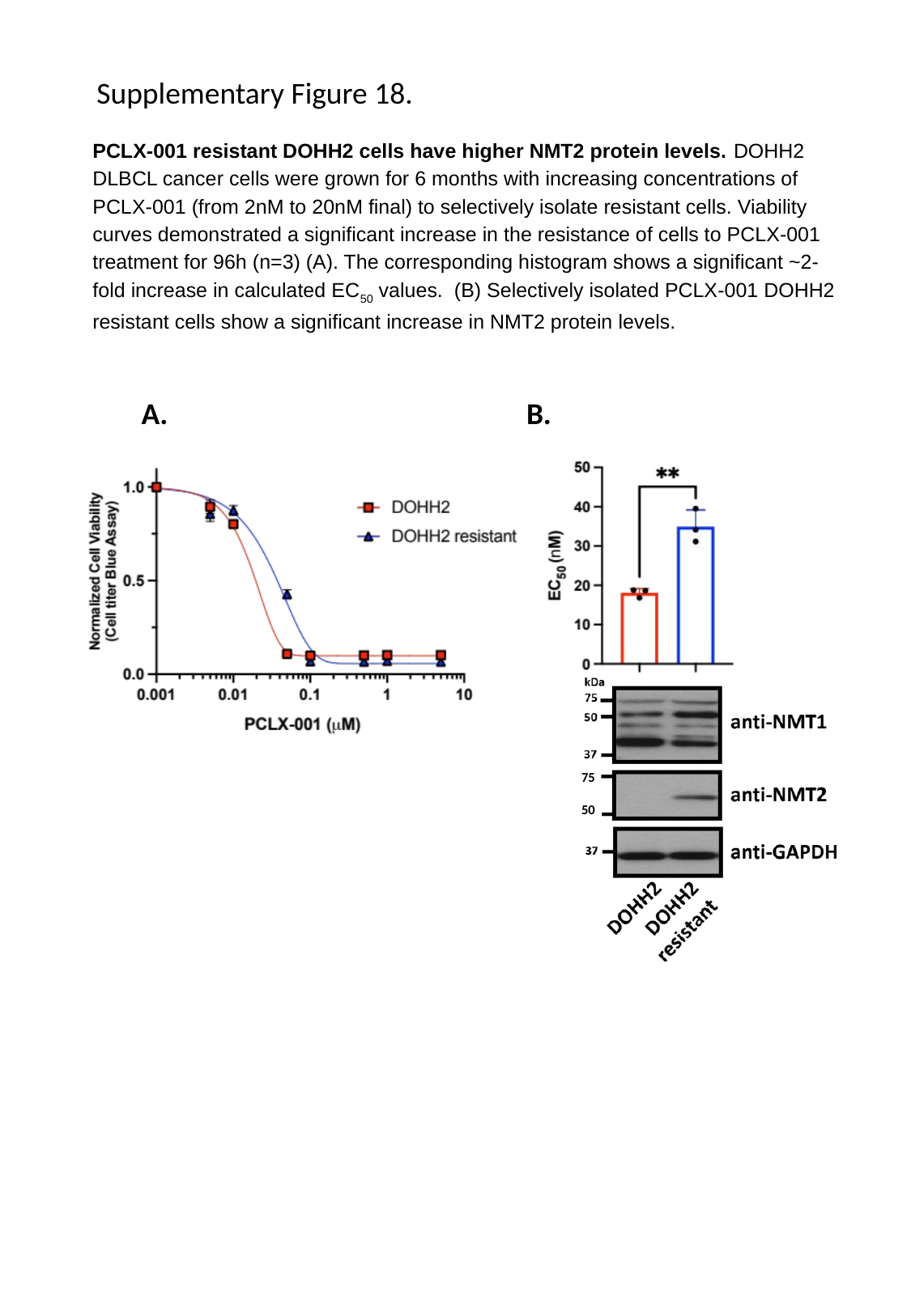

Supplementary Figure 18.
PCLX-001 resistant DOHH2 cells have higher NMT2 protein levels. DOHH2 DLBCL cancer cells were grown for 6 months with increasing concentrations of PCLX-001 (from 2nM to 20nM final) to selectively isolate resistant cells. Viability curves demonstrated a significant increase in the resistance of cells to PCLX-001 treatment for 96h (n=3) (A). The corresponding histogram shows a significant ~2-fold increase in calculated EC50 values. (B) Selectively isolated PCLX-001 DOHH2 resistant cells show a significant increase in NMT2 protein levels.
A.
B.
75
50

## Slide 26
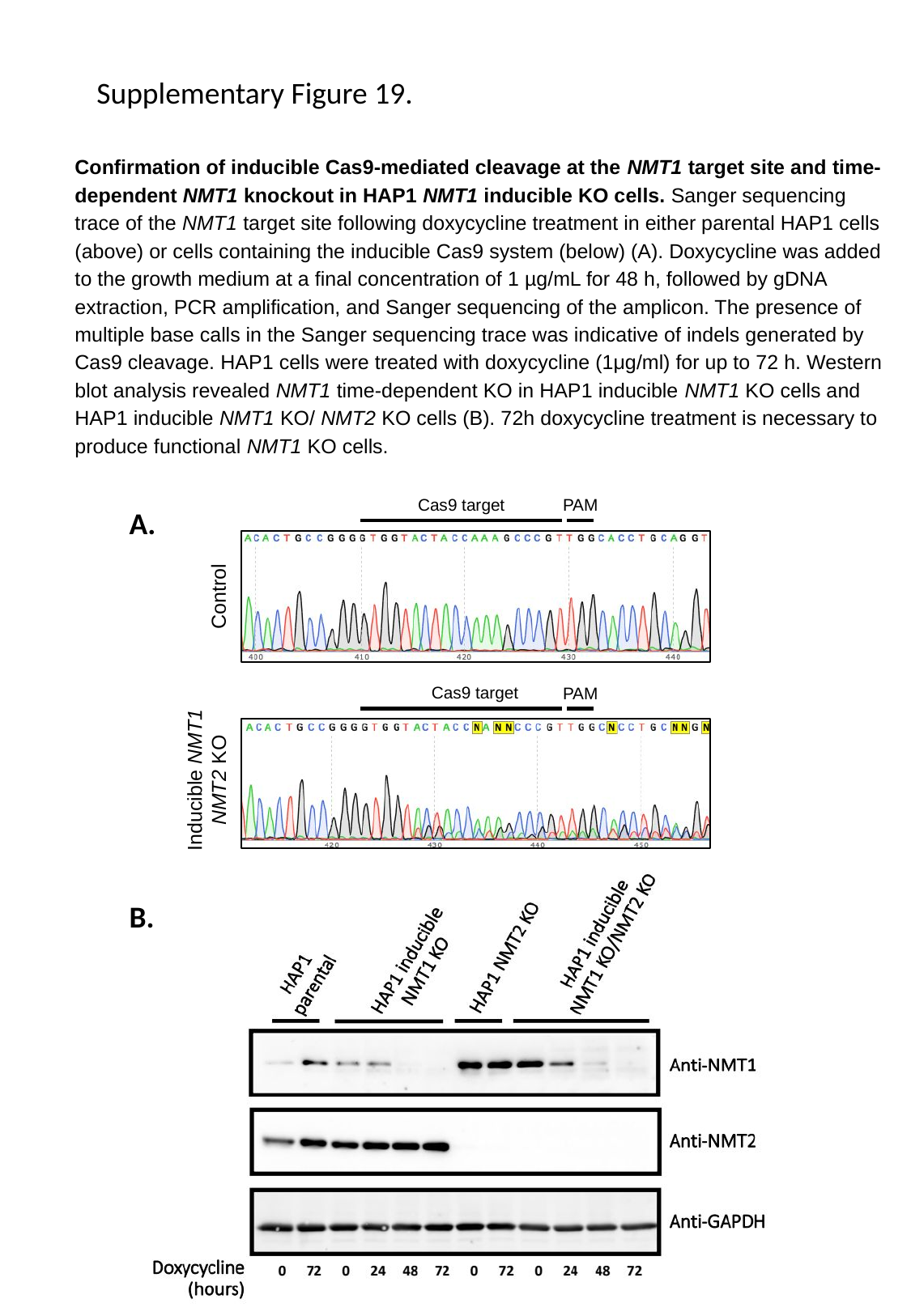

Supplementary Figure 19.
Confirmation of inducible Cas9-mediated cleavage at the NMT1 target site and time-dependent NMT1 knockout in HAP1 NMT1 inducible KO cells. Sanger sequencing trace of the NMT1 target site following doxycycline treatment in either parental HAP1 cells (above) or cells containing the inducible Cas9 system (below) (A). Doxycycline was added to the growth medium at a final concentration of 1 µg/mL for 48 h, followed by gDNA extraction, PCR amplification, and Sanger sequencing of the amplicon. The presence of multiple base calls in the Sanger sequencing trace was indicative of indels generated by Cas9 cleavage. HAP1 cells were treated with doxycycline (1μg/ml) for up to 72 h. Western blot analysis revealed NMT1 time-dependent KO in HAP1 inducible NMT1 KO cells and HAP1 inducible NMT1 KO/ NMT2 KO cells (B). 72h doxycycline treatment is necessary to produce functional NMT1 KO cells.
Cas9 target
PAM
Control
Cas9 target
PAM
Inducible NMT1 NMT2 KO
A.
B.

## Slide 27
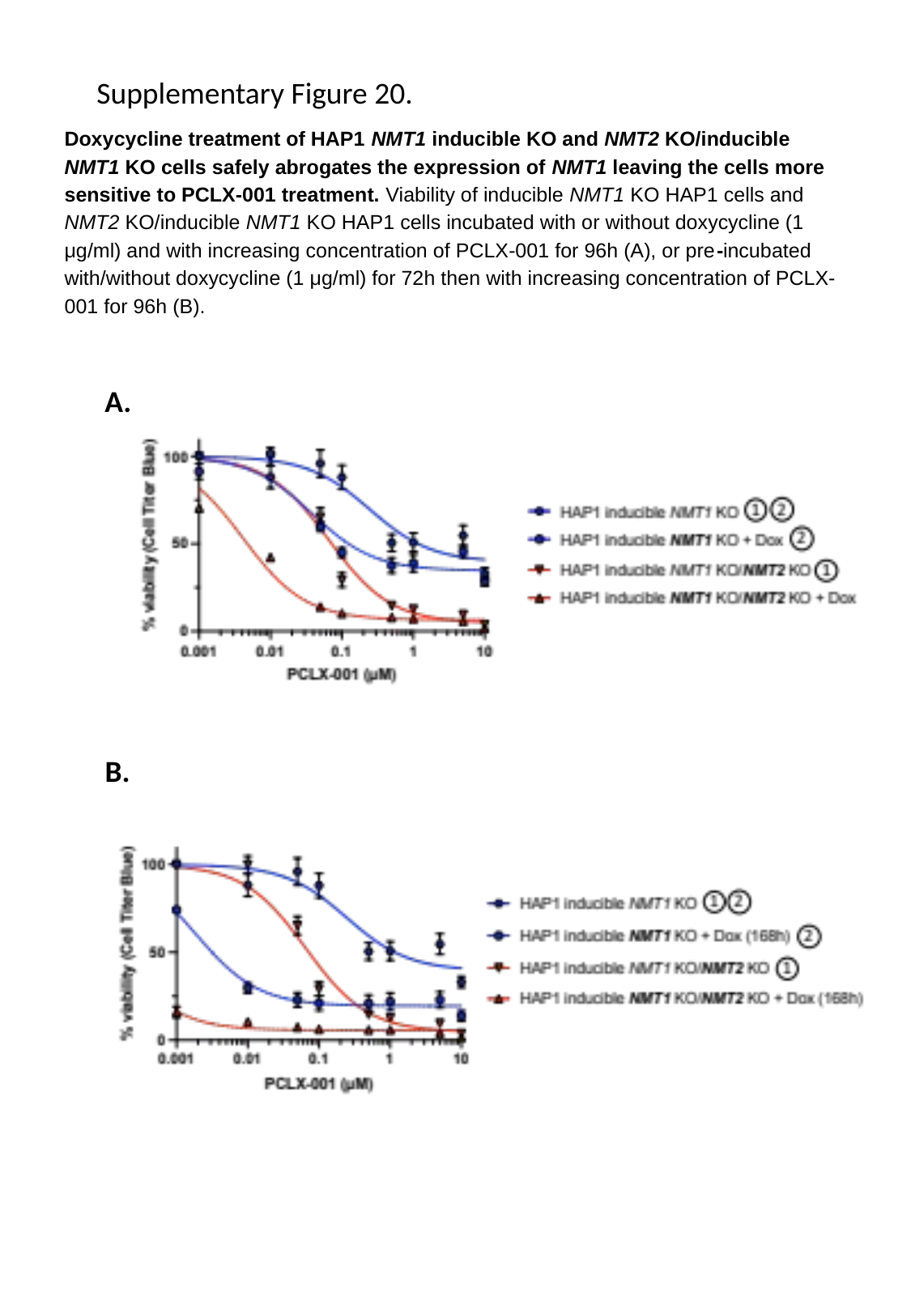

Supplementary Figure 20.
Doxycycline treatment of HAP1 NMT1 inducible KO and NMT2 KO/inducible NMT1 KO cells safely abrogates the expression of NMT1 leaving the cells more sensitive to PCLX-001 treatment. Viability of inducible NMT1 KO HAP1 cells and NMT2 KO/inducible NMT1 KO HAP1 cells incubated with or without doxycycline (1 μg/ml) and with increasing concentration of PCLX-001 for 96h (A), or pre-incubated with/without doxycycline (1 μg/ml) for 72h then with increasing concentration of PCLX-001 for 96h (B).
A.
B.

## Slide 28
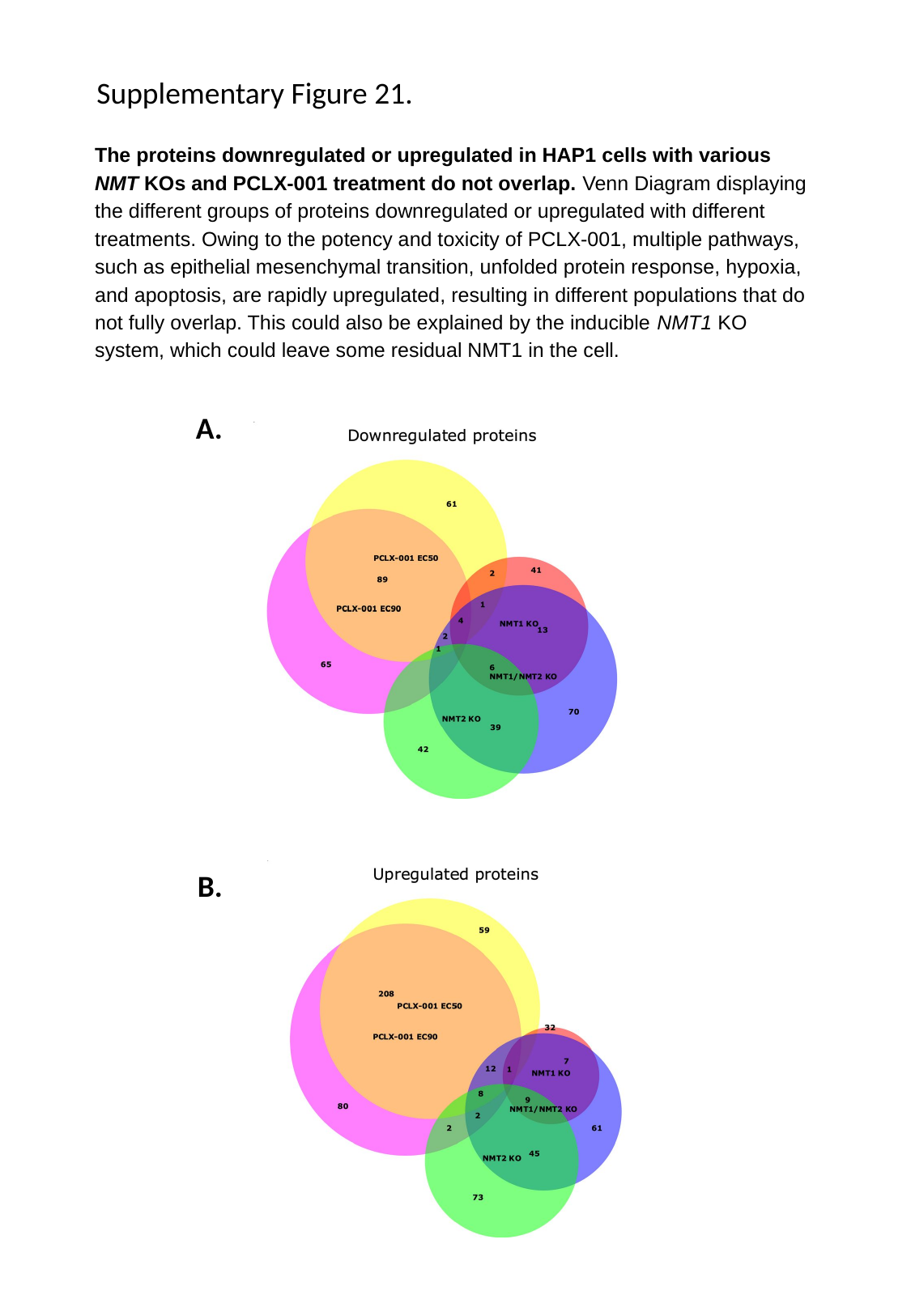

Supplementary Figure 21.
The proteins downregulated or upregulated in HAP1 cells with various NMT KOs and PCLX-001 treatment do not overlap. Venn Diagram displaying the different groups of proteins downregulated or upregulated with different treatments. Owing to the potency and toxicity of PCLX-001, multiple pathways, such as epithelial mesenchymal transition, unfolded protein response, hypoxia, and apoptosis, are rapidly upregulated, resulting in different populations that do not fully overlap. This could also be explained by the inducible NMT1 KO system, which could leave some residual NMT1 in the cell.
A.
B.

## Slide 29
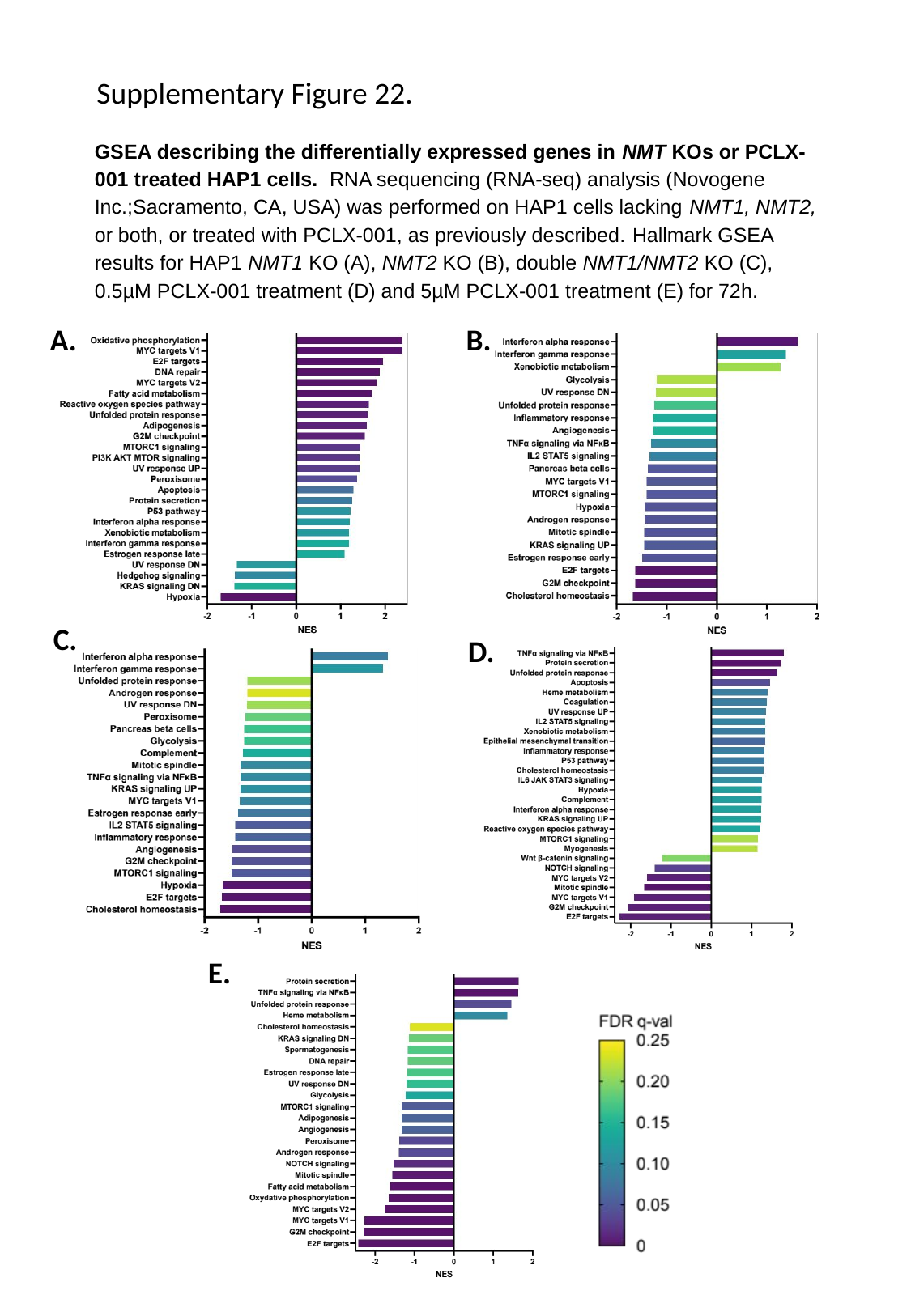

Supplementary Figure 22.
GSEA describing the differentially expressed genes in NMT KOs or PCLX-001 treated HAP1 cells.  RNA sequencing (RNA-seq) analysis (Novogene Inc.;Sacramento, CA, USA) was performed on HAP1 cells lacking NMT1, NMT2, or both, or treated with PCLX-001, as previously described. Hallmark GSEA results for HAP1 NMT1 KO (A), NMT2 KO (B), double NMT1/NMT2 KO (C), 0.5µM PCLX-001 treatment (D) and 5µM PCLX-001 treatment (E) for 72h.
A.
B.
C.
D.
E.

## Slide 30
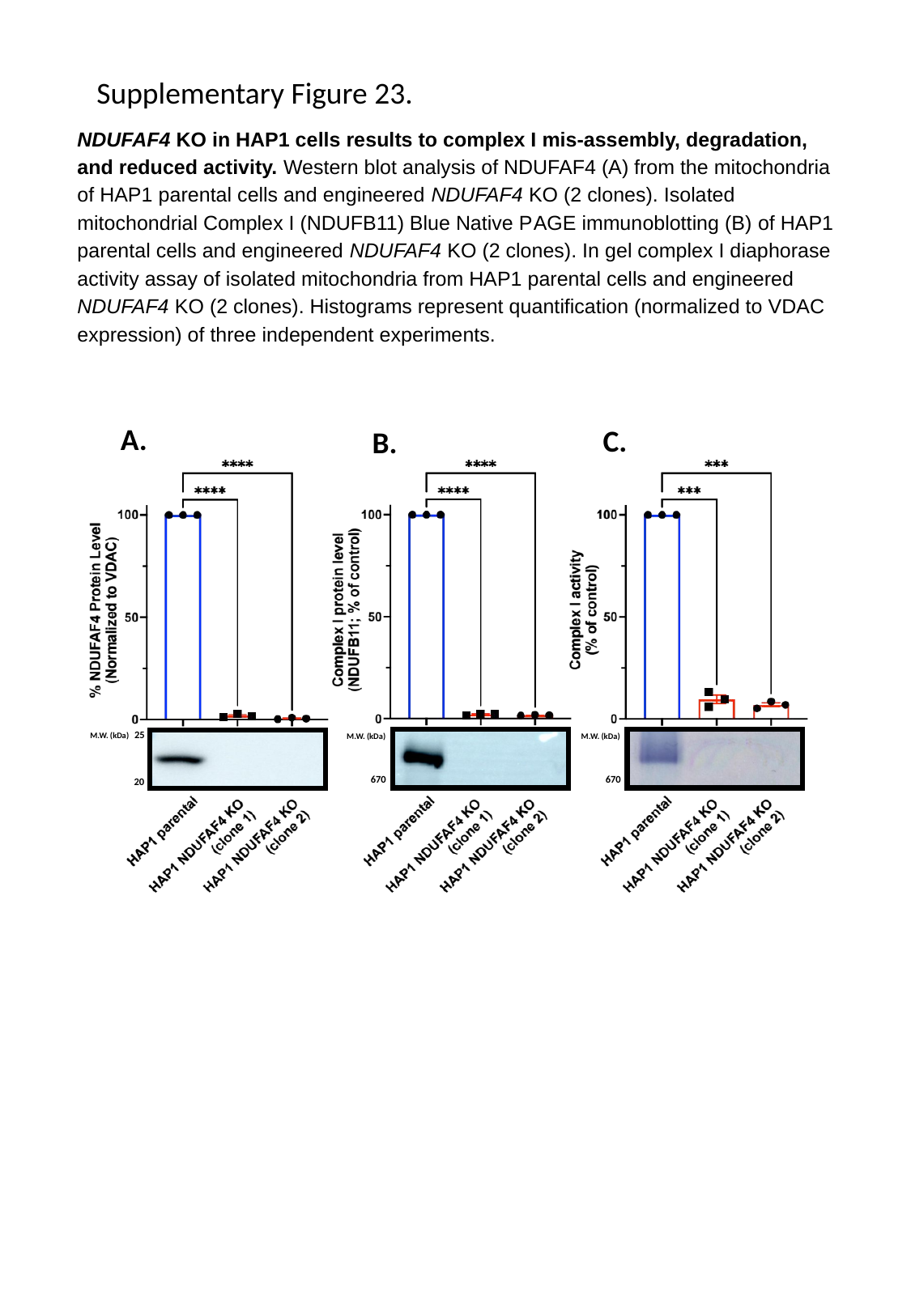

Supplementary Figure 23.
NDUFAF4 KO in HAP1 cells results to complex I mis-assembly, degradation, and reduced activity. Western blot analysis of NDUFAF4 (A) from the mitochondria of HAP1 parental cells and engineered NDUFAF4 KO (2 clones). Isolated mitochondrial Complex I (NDUFB11) Blue Native PAGE immunoblotting (B) of HAP1 parental cells and engineered NDUFAF4 KO (2 clones). In gel complex I diaphorase activity assay of isolated mitochondria from HAP1 parental cells and engineered NDUFAF4 KO (2 clones). Histograms represent quantification (normalized to VDAC expression) of three independent experiments.
A.
C.
B.
M.W. (kDa)
670
25
M.W. (kDa)
20
M.W. (kDa)
670

## Slide 31
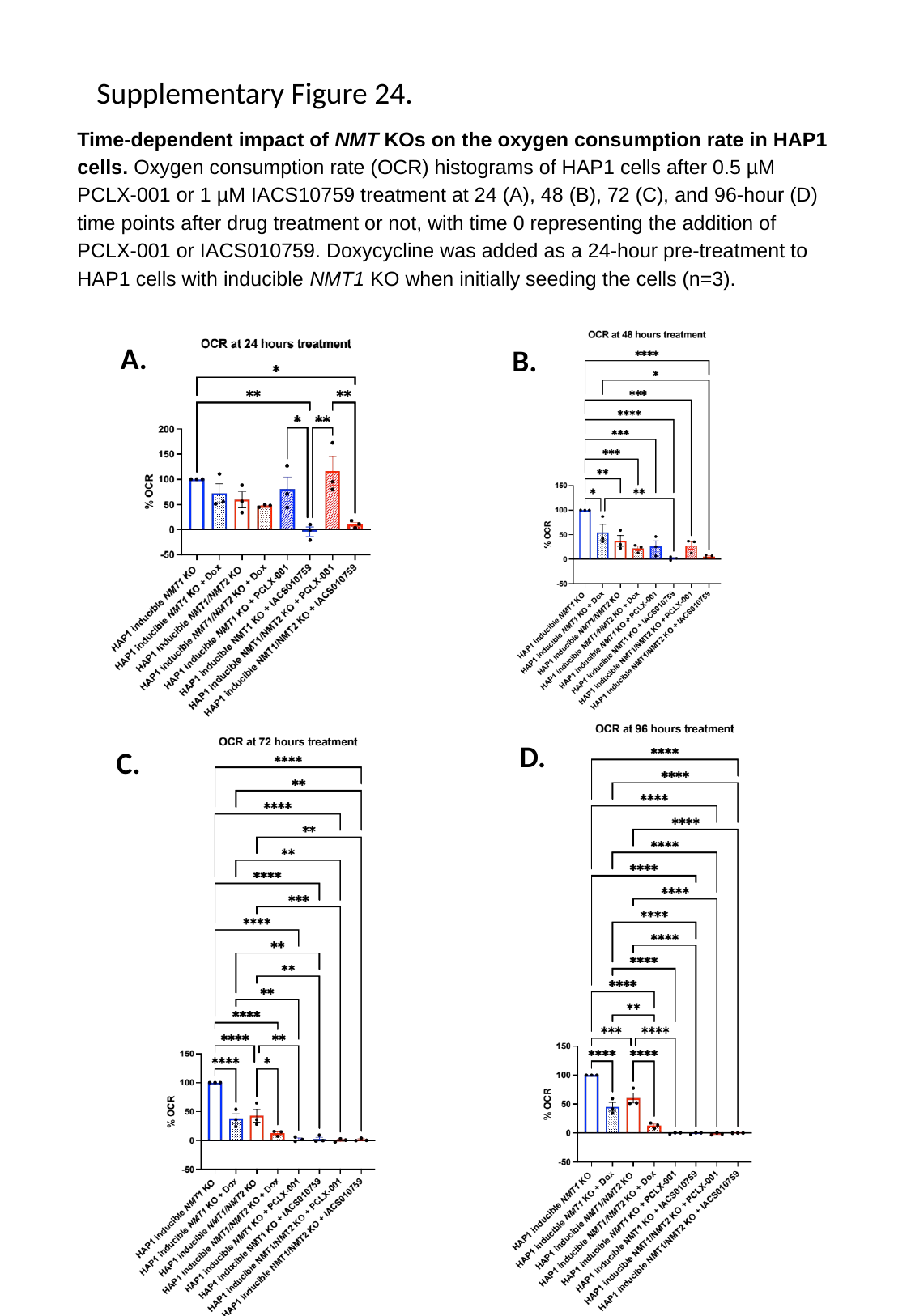

Supplementary Figure 24.
Time-dependent impact of NMT KOs on the oxygen consumption rate in HAP1 cells. Oxygen consumption rate (OCR) histograms of HAP1 cells after 0.5 µM PCLX-001 or 1 µM IACS10759 treatment at 24 (A), 48 (B), 72 (C), and 96-hour (D) time points after drug treatment or not, with time 0 representing the addition of PCLX-001 or IACS010759. Doxycycline was added as a 24-hour pre-treatment to HAP1 cells with inducible NMT1 KO when initially seeding the cells (n=3).
A.
B.
D.
C.

## Slide 32
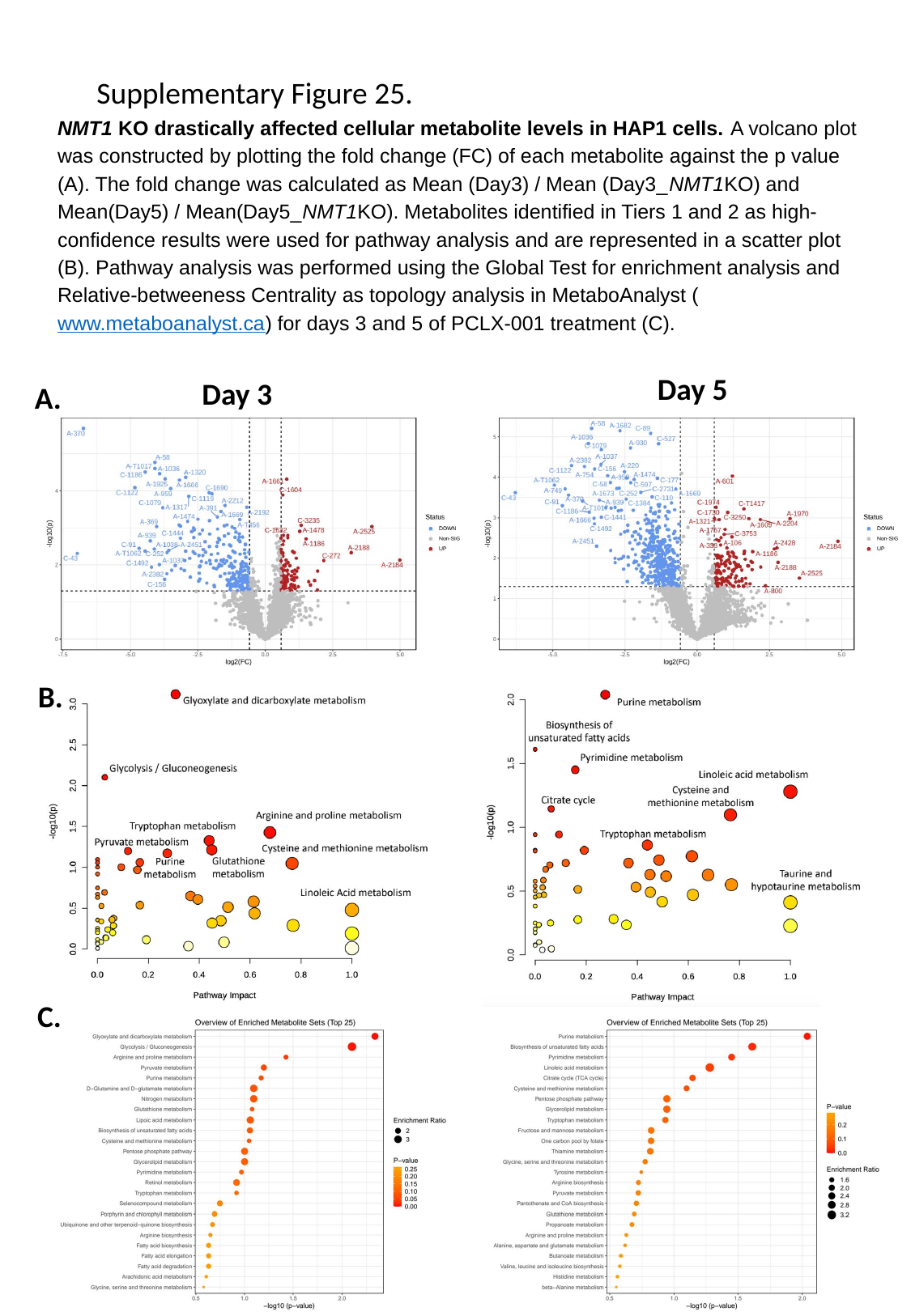

Supplementary Figure 25.
NMT1 KO drastically affected cellular metabolite levels in HAP1 cells. A volcano plot was constructed by plotting the fold change (FC) of each metabolite against the p value (A). The fold change was calculated as Mean (Day3) / Mean (Day3_NMT1KO) and Mean(Day5) / Mean(Day5_NMT1KO). Metabolites identified in Tiers 1 and 2 as high-confidence results were used for pathway analysis and are represented in a scatter plot (B). Pathway analysis was performed using the Global Test for enrichment analysis and Relative-betweeness Centrality as topology analysis in MetaboAnalyst (www.metaboanalyst.ca) for days 3 and 5 of PCLX-001 treatment (C).
Day 5
Day 3
A.
B.
C.

## Slide 33
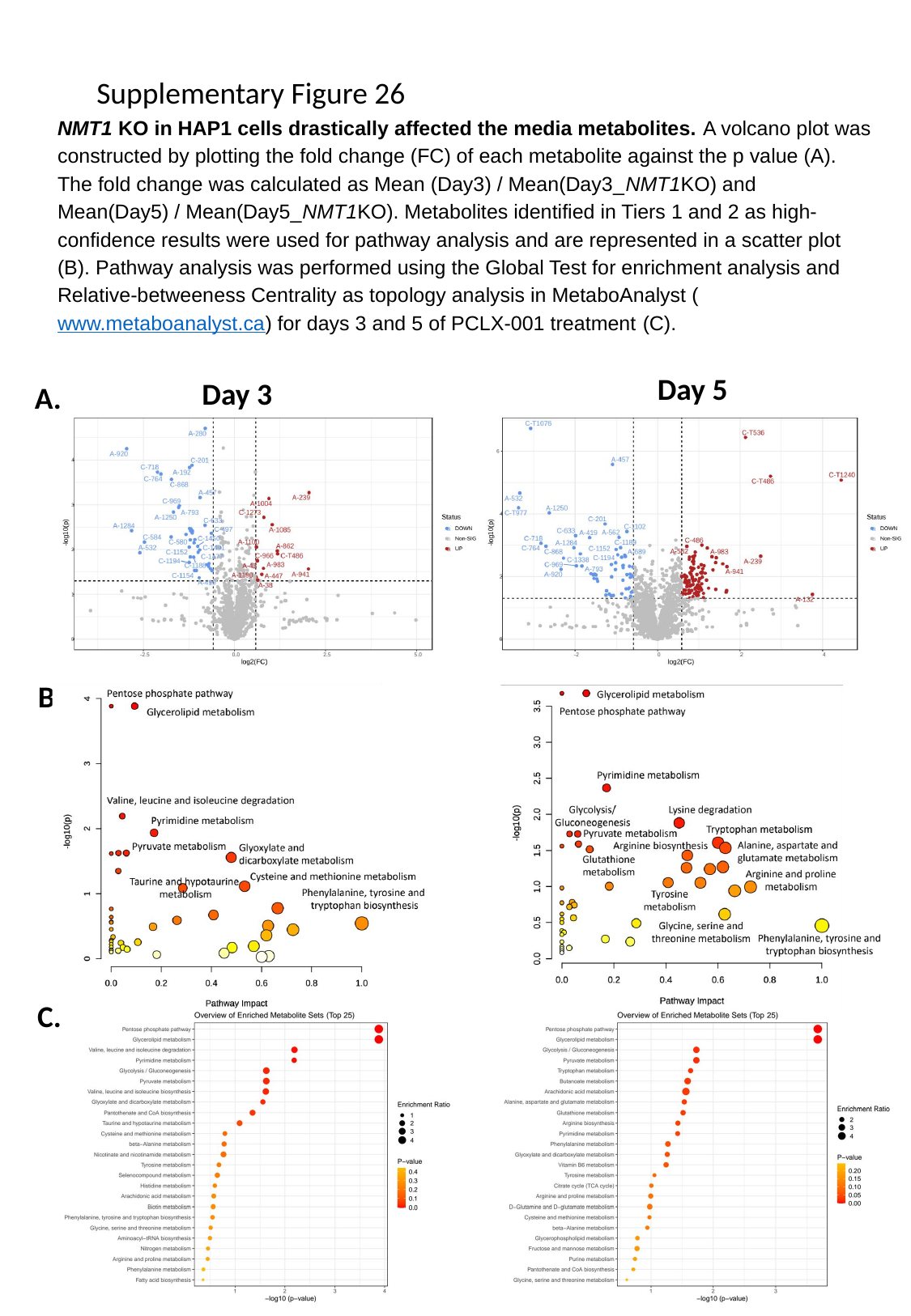

Supplementary Figure 26
NMT1 KO in HAP1 cells drastically affected the media metabolites. A volcano plot was constructed by plotting the fold change (FC) of each metabolite against the p value (A). The fold change was calculated as Mean (Day3) / Mean(Day3_NMT1KO) and Mean(Day5) / Mean(Day5_NMT1KO). Metabolites identified in Tiers 1 and 2 as high-confidence results were used for pathway analysis and are represented in a scatter plot (B). Pathway analysis was performed using the Global Test for enrichment analysis and Relative-betweeness Centrality as topology analysis in MetaboAnalyst (www.metaboanalyst.ca) for days 3 and 5 of PCLX-001 treatment (C).
Day 5
Day 3
A.
B.
C.

## Slide 34
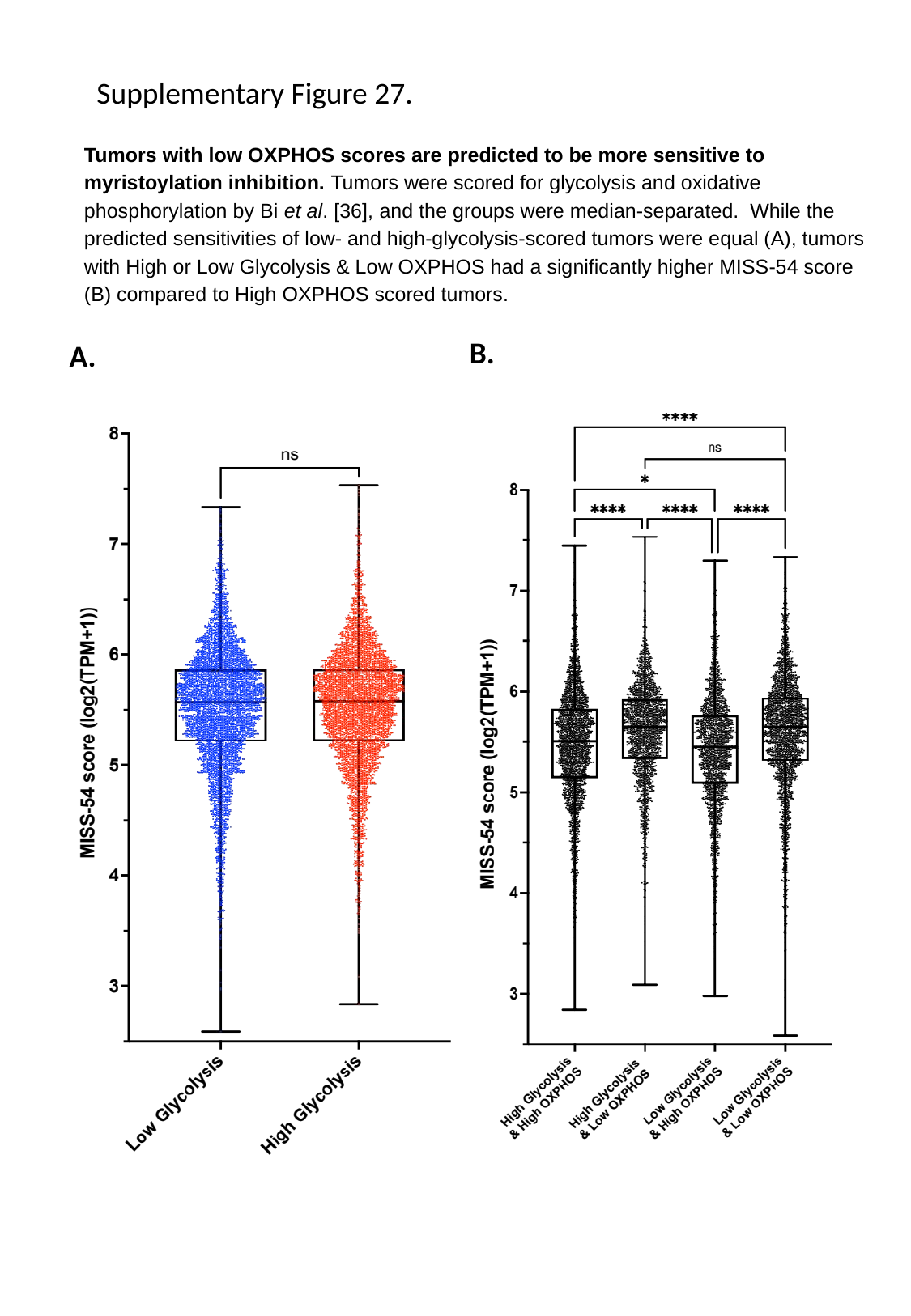

Supplementary Figure 27.
Tumors with low OXPHOS scores are predicted to be more sensitive to myristoylation inhibition. Tumors were scored for glycolysis and oxidative phosphorylation by Bi et al. [36], and the groups were median-separated. While the predicted sensitivities of low- and high-glycolysis-scored tumors were equal (A), tumors with High or Low Glycolysis & Low OXPHOS had a significantly higher MISS-54 score (B) compared to High OXPHOS scored tumors.
B.
A.

## Slide 35
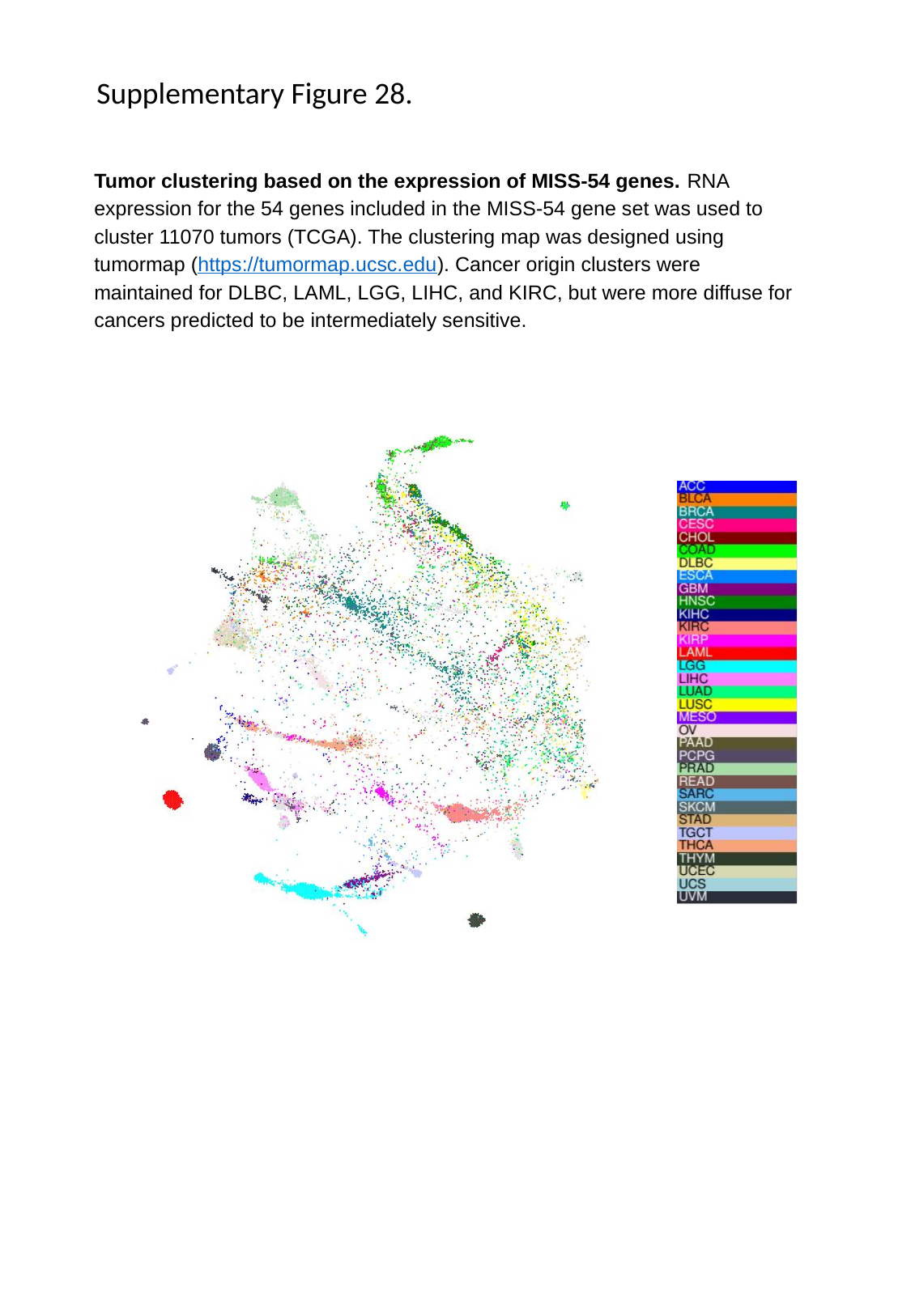

Supplementary Figure 28.
Tumor clustering based on the expression of MISS-54 genes. RNA expression for the 54 genes included in the MISS-54 gene set was used to cluster 11070 tumors (TCGA). The clustering map was designed using tumormap (https://tumormap.ucsc.edu). Cancer origin clusters were maintained for DLBC, LAML, LGG, LIHC, and KIRC, but were more diffuse for cancers predicted to be intermediately sensitive.

## Slide 36
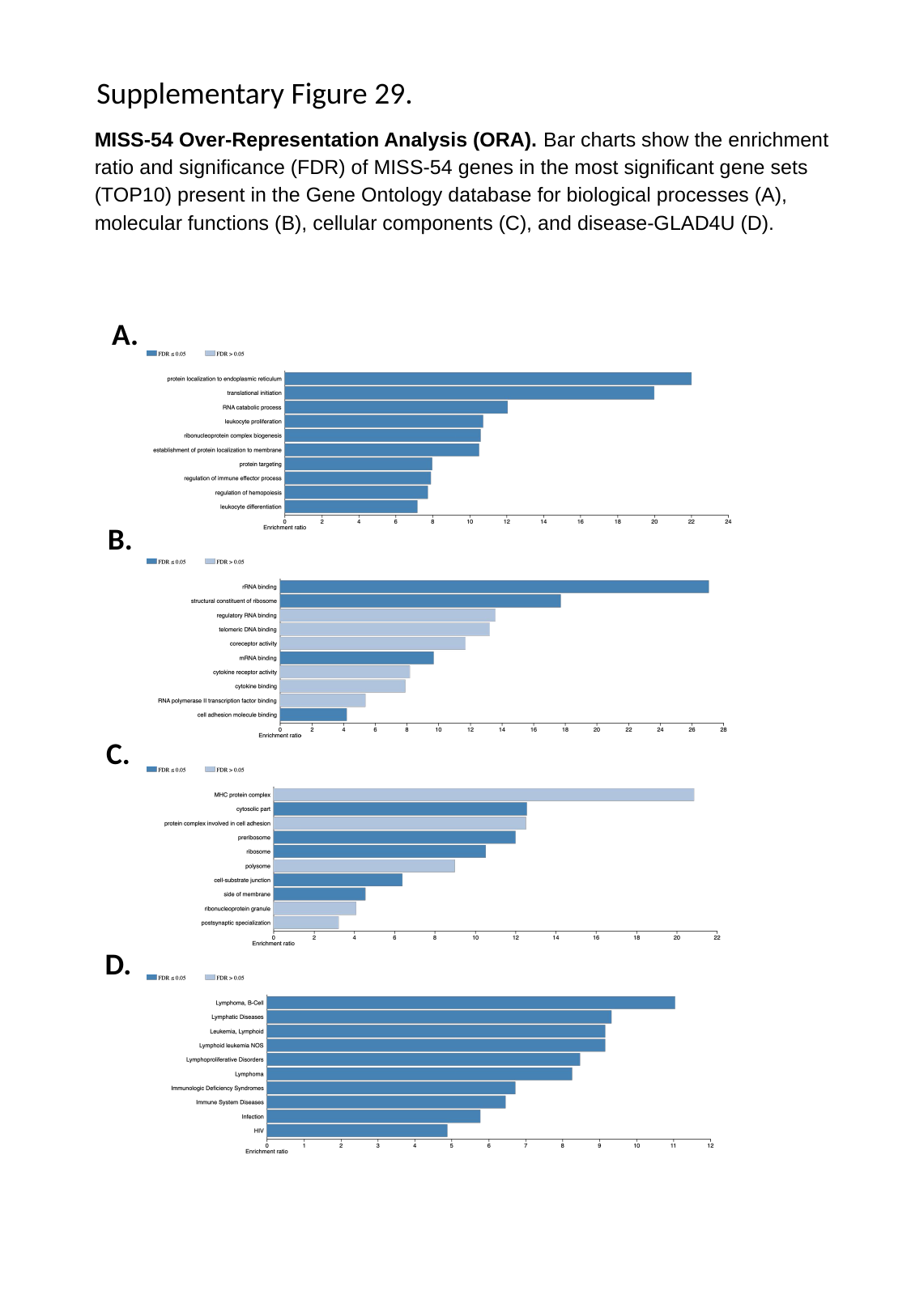

Supplementary Figure 29.
MISS-54 Over-Representation Analysis (ORA). Bar charts show the enrichment ratio and significance (FDR) of MISS-54 genes in the most significant gene sets (TOP10) present in the Gene Ontology database for biological processes (A), molecular functions (B), cellular components (C), and disease-GLAD4U (D).
A.
B.
C.
D.

## Slide 37
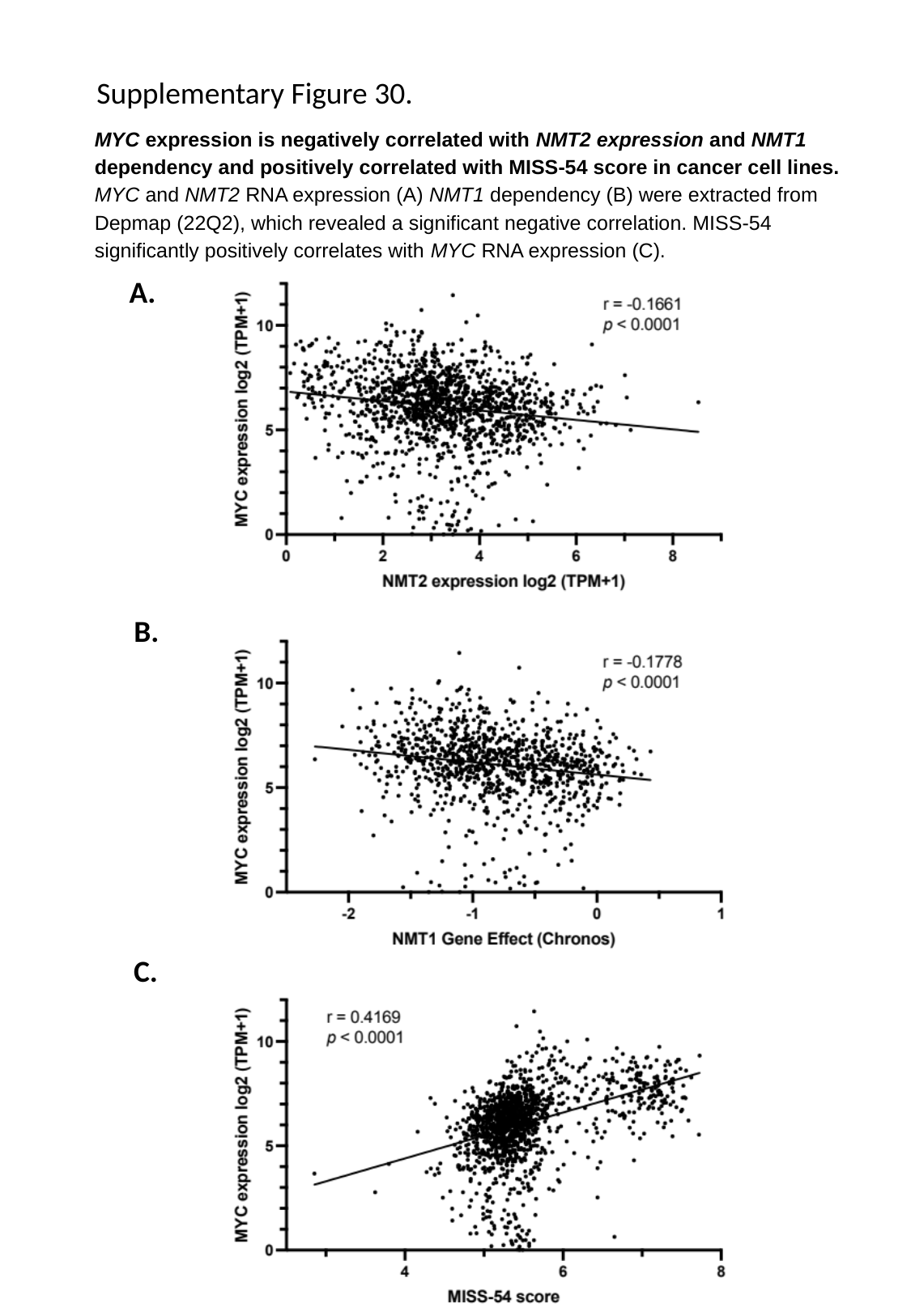

Supplementary Figure 30.
MYC expression is negatively correlated with NMT2 expression and NMT1 dependency and positively correlated with MISS-54 score in cancer cell lines. MYC and NMT2 RNA expression (A) NMT1 dependency (B) were extracted from Depmap (22Q2), which revealed a significant negative correlation. MISS-54 significantly positively correlates with MYC RNA expression (C).
A.
B.
C.
