# Supplementary material for: Multiomics analysis identifies oxidative phosphorylation as a cancer vulnerability arising from myristoylation inhibition
Source: J Transl Med. 2024 May 7;22:431. doi: 10.1186/s12967-024-05150-6 (PMC11075276; doi:10.1186/s12967-024-05150-6)
Supplement: Supplementary file 2 — Additional file 2: Table S1. Univariate and multivariate analyses of high versus low NMT2 mRNA expression as a prognostic marker for progression-free survival in the GSE31312 cohort. High NMT2 indicates an NMT2 mRNA expression level above the median NMT2 level in that cohort, and low NMT2 indicates an NMT2 mRNA expression level below the median in that cohort. HR, hazard ratio; CI, confidence interval; GCB, germinal center B‐cell like; ACB, activated B‐cell like; UC, unclassified. Table S2. Association of NMT2 expression with patient clinicopathological features in DBCL (analyzed from the GSE31312 cohort). Table S3. PCLX-001 and PCLX-002 sensitive cells have multiple origins. The treated cell lines were grouped according to their sensitivity to PCLX-001 and PCLX-002 (IC50). The cell lines were sorted into quartiles representing NMTI-sensitive (0–25%), less sensitive (25–50%), less resistant (50–75%), and resistant (75–100%) cells. The origin of the cells sensitive to PCLX-001 [22] and PCLX-002 [31] is not restricted to hematologic cancers, but could also be found in cells originating from solid tumors. Therefore, MISS-54 is not a signature of hematological cancers. Table S4. List of the 54 genes included in the Myristoylation Inhibition Sensitive Signature (MISS-54). Table S5. TCGA study abbreviations. (https://gdc.cancer.gov/resources-tcga-users/tcga-code-tables/tcga-study-abbreviations) [file 12967_2024_5150_MOESM2_ESM.docx]

**Table S1.** **Univariate and multivariate analyses of high versus low *NMT2* mRNA expression as a prognostic marker for progression-free survival in the GSE31312 cohort.** High *NMT2* indicates an *NMT2* mRNA expression level above the median NMT2 level in that cohort, and low *NMT2* indicates an *NMT2* mRNA expression level below the median in that cohort. HR, hazard ratio; CI, confidence interval; GCB, germinal center B‐cell like; ACB, activated B‐cell like; UC, unclassified.


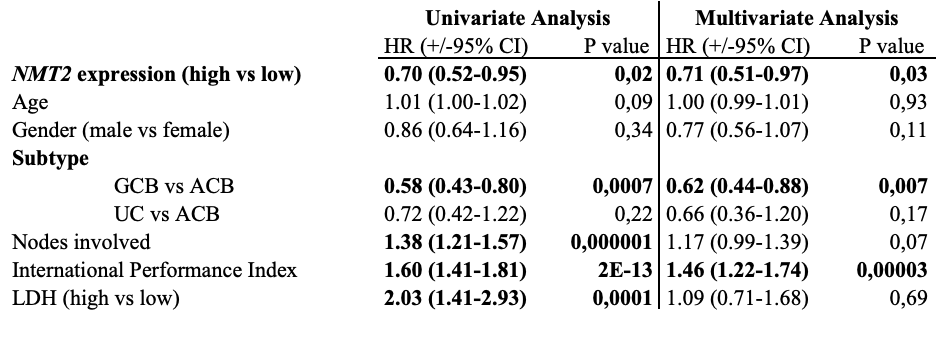


**Table S2. Association of *NMT2* expression with patient clinicopathological features in DBCL (analyzed from the GSE31312 cohort).**

**
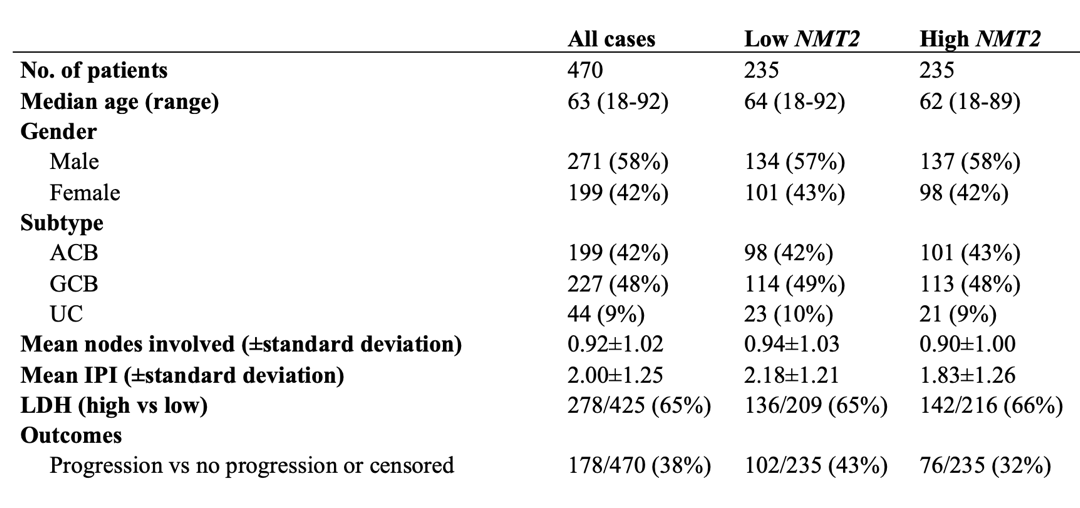
**

**Table S3. PCLX-001 and PCLX-002 sensitive cells have multiple origins. The treated cell lines were grouped according to their sensitivity to PCLX-001 and PCLX-002 (IC50). The cell lines were sorted into quartiles representing NMTI-sensitive (0-25%), less sensitive (25-50%), less resistant (50-75%), and resistant (75-100%) cells. The origin of the cells sensitive to PCLX-001[22] and PCLX-002 [31] is not restricted to hematologic cancers, but could also be found in cells originating from solid tumors. Therefore, MISS-54 is not a signature of hematological cancers.**

**Table S4. List of the 54 genes included in the Myristoylation Inhibition Sensitive Signature (MISS-54).**

| HLA-DQA1 | RPL9 | MAP4K1 | MRTO4 | RPLP0 | FBL |
| --- | --- | --- | --- | --- | --- |
| CD74 | PRKCB | WAS | PPAN | RPL14 | DDX21 |
| CD86 | MAP3K7 | NLRP3 | PPRC1 | RPS3 | ABCE1 |
| CD69 | RPS9 | DYRK3 | HK2 | RPS2 | NAP1L1 |
| ITGA4 | PTPRC | SRGN | SLC25A3 | SERBP1 |  |
| CD38 | ITGB2 | MYC | NPM1 | RPS5 |  |
| IL12RB1 | BCAT1 | RPA2 | GNL3 | RPS6 |  |
| IL2RA | FCGR2B | IMPDH2 | RPL34 | HNRNPA1 |  |
| HCLS1 | EIF3A | LAS1L | RPL6 | RPS10 |  |
| SPI1 | CD79A | FARSA | HNRNPC | RSL1D1 |  |

**Table S5. TCGA study abbreviations**. (<https://gdc.cancer.gov/resources-tcga-users/tcga-code-tables/tcga-study-abbreviations>)

| **LAML** | **Acute Myeloid Leukemia** |
| --- | --- |
| **ACC** | **Adrenocortical carcinoma** |
| **BLCA** | **Bladder Urothelial Carcinoma** |
| **LGG** | **Brain Lower Grade Glioma** |
| **BRCA** | **Breast invasive carcinoma** |
| **CESC** | **Cervical squamous cell carcinoma and endocervical adenocarcinoma** |
| **CHOL** | **Cholangiocarcinoma** |
| **LCML** | **Chronic Myelogenous Leukemia** |
| **COAD** | **Colon adenocarcinoma** |
| **CNTL** | **Controls** |
| **ESCA** | **Esophageal carcinoma** |
| **FPPP** | **FFPE Pilot Phase II** |
| **GBM** | **Glioblastoma multiforme** |
| **HNSC** | **Head and Neck squamous cell carcinoma** |
| **KICH** | **Kidney Chromophobe** |
| **KIRC** | **Kidney renal clear cell carcinoma** |
| **KIRP** | **Kidney renal papillary cell carcinoma** |
| **LIHC** | **Liver hepatocellular carcinoma** |
| **LUAD** | **Lung adenocarcinoma** |
| **LUSC** | **Lung squamous cell carcinoma** |
| **DLBC** | **Lymphoid Neoplasm Diffuse Large B-cell Lymphoma** |
| **MESO** | **Mesothelioma** |
| **MISC** | **Miscellaneous** |
| **OV** | **Ovarian serous cystadenocarcinoma** |
| **PAAD** | **Pancreatic adenocarcinoma** |
| **PCPG** | **Pheochromocytoma and Paraganglioma** |
| **PRAD** | **Prostate adenocarcinoma** |
| **READ** | **Rectum adenocarcinoma** |
| **SARC** | **Sarcoma** |
| **SKCM** | **Skin Cutaneous Melanoma** |
| **STAD** | **Stomach adenocarcinoma** |
| **TGCT** | **Testicular Germ Cell Tumors** |
| **THYM** | **Thymoma** |
| **THCA** | **Thyroid carcinoma** |
| **UCS** | **Uterine Carcinosarcoma** |
| **UCEC** | **Uterine Corpus Endometrial Carcinoma** |
| **UVM** | **Uveal Melanoma** |
